# Aberrant chromatin landscape following loss of the H3.3 chaperone Daxx in haematopoietic precursors leads to Pu.1-mediated neutrophilia and inflammation

**DOI:** 10.1038/s41556-021-00774-y

**Published:** 2021-12-07

**Authors:** Julia P. Gerber, Jenny Russ, Vijay Chandrasekar, Nina Offermann, Hang-Mao Lee, Sarah Spear, Nicola Guzzi, Simona Maida, Sundararaghavan Pattabiraman, Ruoyu Zhang, Amir H. Kayvanjoo, Preeta Datta, Jagath Kasturiarachchi, Teresa Sposito, Natalia Izotova, Kristian Händler, Peter D. Adams, Teresa Marafioti, Tariq Enver, Jörg Wenzel, Marc Beyer, Elvira Mass, Cristian Bellodi, Joachim L. Schultze, Melania Capasso, Rachael Nimmo, Paolo Salomoni

**Affiliations:** 1 German Center for Neurodegenerative Diseases (DZNE), Bonn, Germany; 2 Department of Cancer Biology, UCL Cancer Institute, London, UK; 3 Barts Cancer Institute, Queen Mary University of London, London, UK; 4 Division of Molecular Hematology, Department of Laboratory Medicine, Lund Stem Cell Center, Faculty of Medicine, Lund University, Lund, Sweden; 5 Life and Medical Sciences (LIMES) Institute, Developmental Biology of the Immune System, University of Bonn, Bonn, Germany; 6 Platform for Single Cell Genomics and Epigenomics (PRECISE) at the German Center for Neurodegenerative Diseases and the University of Bonn, Bonn, Germany; 7 Sanford Burnham Prebys Medical Discovery Institute, La Jolla, USA; 8 Department of Pathology, University College London, London, UK; 9 Department of Dermatology and Allergy, University Medical Center, Bonn, Germany; 10 Genomics and Immunoregulation, LIMES Institute, University of Bonn, Bonn, Germany

**Keywords:** Chronic inflammation, Haematopoietic stem cells

## Abstract

Defective silencing of retrotransposable elements has been linked to inflammageing, cancer and autoimmune diseases. However, the underlying mechanisms are only partially understood. Here we implicate the histone H3.3 chaperone Daxx, a retrotransposable element repressor inactivated in myeloid leukaemia and other neoplasms, in protection from inflammatory disease. Loss of Daxx alters the chromatin landscape, H3.3 distribution and histone marks of haematopoietic progenitors, leading to engagement of a Pu.1-dependent transcriptional programme for myelopoiesis at the expense of B-cell differentiation. This causes neutrophilia and inflammation, predisposing mice to develop an autoinflammatory skin disease. While these molecular and phenotypic perturbations are in part reverted in animals lacking both Pu.1 and Daxx, haematopoietic progenitors in these mice show unique chromatin and transcriptome alterations, suggesting an interaction between these two pathways. Overall, our findings implicate retrotransposable element silencing in haematopoiesis and suggest a cross-talk between the H3.3 loading machinery and the pioneer transcription factor Pu.1.

## Abstract (web summary)

Gerber, Russ et al. show that the H3.3 chaperone Daxx, which represses endogenous retroviral and retrotransposable elements, acts as an epigenetic barrier to control haematopoietic progenitor plasticity and protect against PU.1-mediated inflammation.

## Main

Repeat elements, including endogenous retroviral elements (ERVs), retrotransposable elements (RTEs) and telomeric repeats, represent more than half of the human genome. ERVs and RTEs (ERVs/RTEs) have emerged as important regulators of key DNA- and RNA-based cellular functions, such as assembly of transcriptional networks, splicing and mutagenesis^1–3^. Aberrant opening and/or expression of ERVs/RTEs has been linked to inflammageing, cancer and autoimmunity^4–8^. Given the pleiotropic roles of ERVs/RTEs and their potentially detrimental effects on cell and tissue homeostasis, several mechanisms are in place to regulate their accessibility and expression. In embryonic stem cells, DNA methylation and incorporation of the histone 3.3 (H3.3) variant via the Death domain-associated protein (Daxx)–Alpha-thalassaemia X-linked mental retardation (Atrx) complex restricts accessibility to selected RTEs, such as intracisternal particles (IAPs)^9,10^. Loss of ERV/RTE silencing may activate their enhancer function in embryonic stem cells^11^. Notably, loss-of-function mutations in DNA methyltransferases, DAXX and ATRX are found in human cancer, including myeloid malignancies^12–16^. However, their impact on tissue homeostasis, inflammation and oncogenesis remains only partially understood.

## Daxx regulates the HSC chromatin landscape and transcriptome

**Fig. 1:**
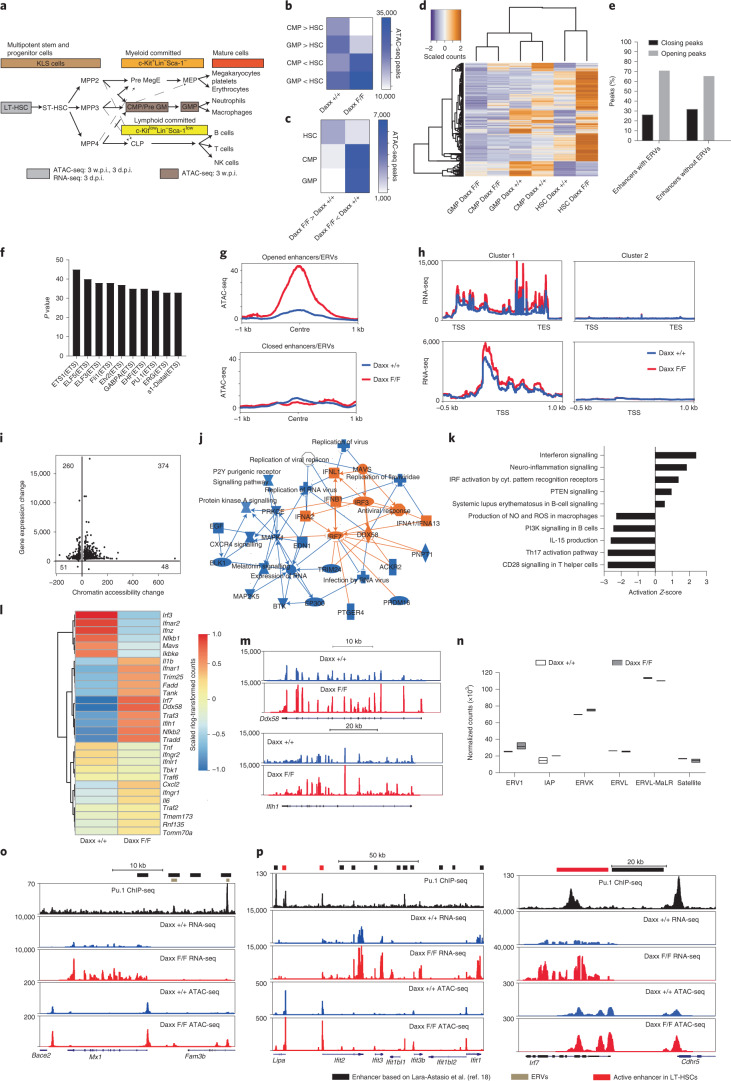
Daxx loss alters the chromatin landscape and transcription in LT-HSCs. **a**, Overview scheme of haematopoiesis. ST-HSC, short-term haematopoietic stem cells; MegE, megakaryocyte-erythrocyte; MEP, megakaryocyte-erythrocyte progenitor; GM, granulocyte-monocyte. **b**, Heatmap of the number of distal peaks that were open or closed in CMPs (open, 8,753 in Daxx-KO and 18,194 in Daxx-WT cells; closed, 28,724 in Daxx-KO and 18,465 in Daxx-WT cells) and GMPs (open, 12,713 in Daxx-KO and 22,833 in Daxx-WT cells; closed, 33,374 in Daxx-KO and 23,703 in Daxx-WT cells) compared with HSCs. **c**, Heatmap of enhancer-overlapping distal peaks that were open or closed in Daxx-KO compared with WT cells. **b**,**c**, >, open peaks; and <, closed peaks. **d**, Heatmap showing scaled read counts within the enhancer-overlapping distal peaks that were open in Daxx-KO LT-HSCs compared with WT cells. **e**, Total number of peaks of LT-HSCs that closed or opened in known enhancers following Daxx KO. **f**, Top-ten transcription factor motifs enriched in ERV-overlapping enhancer peaks open in Daxx-KO LT-HSCs. **g**, ATAC-seq coverage around the centre of enhancers overlapping ERVs. **h**, RNA-seq coverage across gene bodies (left) and around the TSS (right) of genes closest to opened enhancers overlapping ERVs. TES, transcription end site. **i**, Change in accessibility at enhancers overlapping ERVs versus changes in gene expression of the closest gene. Numbers show the total number of enhancer-gene pairs per quadrant. **j**, Summary plot of IPA analysis for the RNA-seq of LT-HSCs. Predicted activation of protein or biofunction is indicated in orange and predicted inhibition of the displayed protein or biofunction in blue. **k**, Top-five activated and repressed IPA canonical pathways. Data are the activation *Z*-scores from IPA Fisher’s exact tests with multiple testing-adjusted *P* *<* 0.05. Activation *Z*-score > 2, increased activation; activation *Z*-score < −2, increased inhibition. cyt., cytoplasmic. **l**, Heatmap showing expression of interferon-responsive genes and dsRNA-recognition machinery in LT-HSCs. **m**, Genome browser tracks of the *Ddx58* (top) and *Ifih1* (bottom) genes. Transcript structure and position are shown below. **n**, Counts per million of repeat-element families in the LT-HSC RNA-seq data. Boxplots show the minimum and maximum values (box boundaries) and the mean (horizontal line). **o**, Genome browser track of the *Mx1* gene regulatory region. Transcripts within the region are shown below. **p**, Genome browser tracks of the *Irf7* (right) and *Ifit* (left) gene clusters. Transcripts within the clusters are shown below. HSC/LT-HSC, long-term haematopoietic stem cells. **a**–**f**, *n* = 2 independent biological samples analysed at 3 w.p.i. **g**–**p**, *n* = 2 (*Daxx*^+/+^ and *Daxx*^+/F^) and 3 (Daxx KO) independent biological samples collected at 3 d.p.i. Daxx F/F, Daxx KO and Daxx +/+, Daxx WT. Numerical source data are provided. Source data

**Extended Data Fig. 1:**
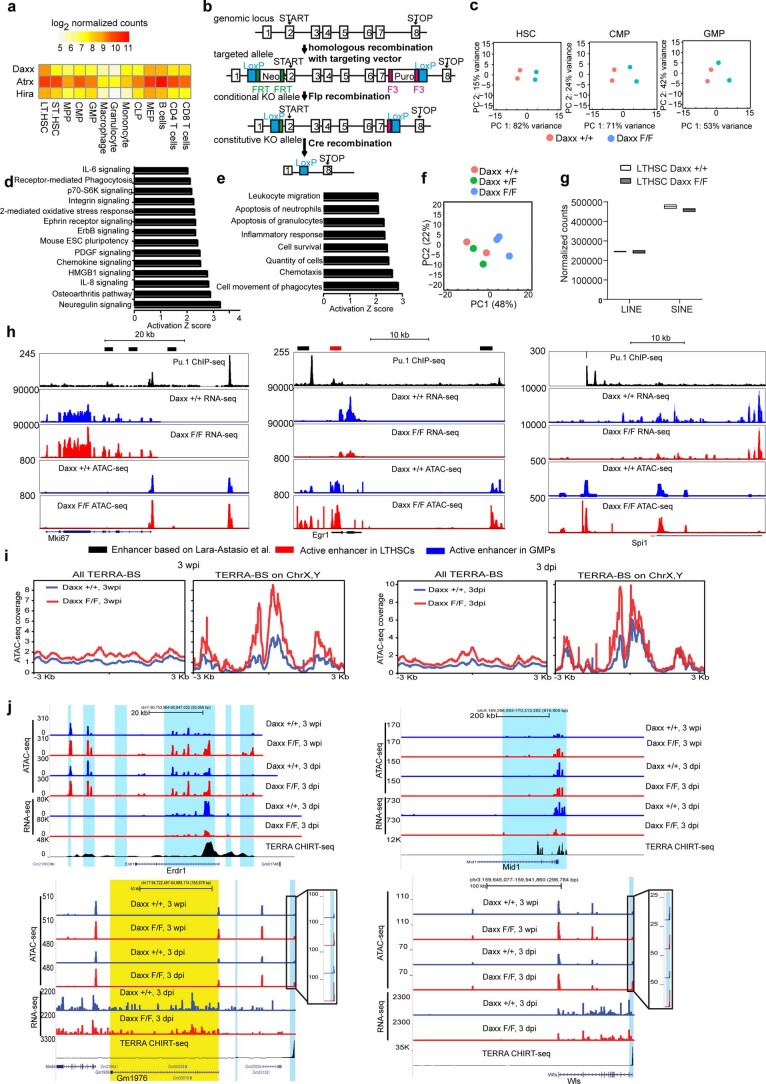
Expression of H3.3 chaperones in haematopoietic cells, creating Daxx F/F mice and analysis of LT-HSC ATAC-seq and RNA-seq data. **a**, Heatmap of Daxx, Atrx and Hira expression levels (data from^18^). **b**, *Daxx* genomic locus, *Daxx* floxed locus following homologous recombination, conditional KO allele following deletion of the FRT-flanked or F3-flanked Neomycin (NeoR) and Puromycin (PuroR) resistance gene; Cre-mediated deletion of Daxx exons 2-7. **c**, PCA for ATAC-seq data (3 w.p.i.; top-500 most variable peaks): displayed principal components (PC) 1 versus 2. **d**, Enriched IPA immune system-related canonical pathways for ERV-overlapping enhancer peaks that open upon Daxx KO in LT-HSCs (3 w.p.i.). **e**, Enriched IPA immune system-related biofunctions for ERV-overlapping enhancer peaks that open upon Daxx KO in LT-HSCs (3 w.p.i.). **f**, PCA plot for LT-HSC RNA-seq data (3 d.p.i.). **g**, Counts per million of LINE and SINE elements in LT-HSCs (3 d.p.i.). **h**, Genome browser tracks of the regulatory region of *Spi1*, *Mki67* and *Egr1* (3 d.p.i.). **i**, ATAC-seq coverage around TERRA-BS across all chromosomes or across sex chromosomes at 3 w.p.i. and 3 d.p.i. **j**, Genome browser tracks for wild-type (blue) and Daxx KO (red) LT-HSCs (ATAC-seq: 3 w.p.i. and 3 d.p.i.; RNA-seq: 3 d.p.i.) around *Erdr1, Mid1, Gm1976* and *Wls*; TERRA CHIRT-seq based on published data^23^. Light-blue regions are called TERRA binding sites and yellow region highlights the *Gm1976* gene. For *Gm1976* and *Wls* boxes show ATAC-seq coverage at the major TERRA binding site. LT.HSC, long-term haematopoietic stem cells; ST.HSC, short-term haematopoietic stem cells; MPP, multipotent progenitor; CMP, common myeloid progenitor; GMP, granulocyte-monocyte progenitor; CLP, common lymphoid progenitor; MEP, megakaryocyte erythroid progenitor. *n* = 2 independent biological samples analysed 3 weeks post induction with tamoxifen (**c**−**e, i, j**). Analysis of gene expression and chromatin accessibility of LT-HSCs from *n* = 2 Daxx + /+ and Daxx + /F as well as *n* = 3 Daxx F/F samples collected 3 days post final induction with tamoxifen (**f-j**). Data are Activation Z-score from IPA Fisher’s exact tests with multiple testing adjusted p-values < 0.05 (**d, e**). Activation Z-score > 2 suggests increased activation of shown biofunctions. Numerical source data provided in Source data. Source data

Expression of Daxx, Atrx and the other H3.3 chaperone Hira^17^ during haematopoiesis is enriched in long-term haematopoietic stem cells (LT-HSCs), megakaryocyte-erythrocyte progenitors and B cells (Extended Data Fig. 1a; based on^18^). To assess the impact of Daxx loss on the chromatin landscape of haematopoietic stem and progenitor cells (HSPCs), we crossed a conditional Daxx-knockout (KO) line with *Rosa26CreERT2* mice (Extended Data Fig. 1b). Sorted LT-HSCs, common myeloid progenitors (CMPs) and granulocyte-monocyte progenitors (GMPs) from wild-type (WT; *Daxx*^+/+^;*RosaCre*^ERT2+/–^) and Daxx-KO (*Daxx*^F/F^;*RosaCre*^ERT2+/–^) mice were processed 3 weeks post tamoxifen treatment/induction (w.p.i.) for transposase-accessible chromatin sequencing (ATAC-seq; Fig. 1a). Principal component analysis (PCA) showed separate clustering of WT and Daxx-KO samples (Extended Data Fig. 1c). While the number of distal regions opening or closing in CMPs or GMPs compared with LT-HSCs were similar in the WT mice, substantially fewer distal regions were open in the Daxx-KO animals (Fig. 1b); most remained closed, suggesting Daxx-KO CMPs and GMPs became more restricted in their gene expression.

By overlapping accessible regions with known haematopoietic enhancers, we found more enhancers opening in KO LT-HSCs (2,509) compared with CMPs and GMPs (1,040 and 716, respectively; Fig. 1c). Based on unsupervised clustering (Fig. 1d), one set of enhancers showed increased read counts in KO LT-HSCs compared with WT LT-HSCs but reduced counts in CMPs and GMPs. Another set displayed similar counts in WT CMPs and GMPs as well as KO LT-HSCs, suggesting that chromatin features of Daxx-KO LT-HSCs may resemble myeloid-committed progenitors. The KO LT-HSCs displayed increased general opening of enhancers, including those overlapping with ERVs, in accordance with a Daxx-repressive role (Fig. 1e). ERV-overlapping enhancers were enriched in transcription factor motifs for the master regulators of haematopoiesis Pu.1 and Ets1 (Fig. 1f). Neighbouring genes displayed enrichment in immune-cell function and pathways associated with myeloid-committed progenitors (Extended Data Fig. 1d,e).

More acute effects of Daxx loss were assessed by collecting mice at 3 d post induction (d.p.i.). Like at 3 w.p.i., most changes occurred at distal regions. However, more sites displayed decreased (8,421) than increased accessibility (3,247). Despite a similar number of known haematopoietic enhancers being opened or closed (1,020 versus 1,022), more enhancers overlapping with ERVs opened (818 versus 617), with more pronounced differences at opened ERV-overlapping enhancers (Fig. 1g). RNA sequencing (RNA-seq; Extended Data Fig. 1f and Supplementary Table 1) showed increased expression of genes close to opened enhancers and ERV-overlapping enhancers (Fig. 1h,i). However, genes proximal to enhancers with reduced accessibility also showed increased expression, an effect potentially due to enhancer occupancy by transcription factors (Fig. 1i).

Ingenuity pathway analysis (IPA) and Gene Ontology suggested engagement of the double-stranded RNA (dsRNA)-recognition machinery and activation of interferon (IFN)-stimulated genes (ISGs; Fig. 1j–m and Supplementary Table 2). Key components of these pathways were indeed upregulated (for example, *Irf3*, *Irf7*, *Mavs* and *Ddx58*; Fig. 1l,m) along with selected ERV subtypes, including the Daxx targets ERV1 and IAPs, but not non-ERV RTEs (Fig. 1n and Extended Data Fig. 1g). Enriched cell cycle-related pathways, along with upregulation of the proliferation marker *Mki67* and downregulation of the quiescence gene *Egr1*, pointed at increased cell-cycle entry (Extended Data Fig. 1h and Supplementary Tables 1,2). Notably, upregulated genes such as *Mx1*, the *Ifit* cluster, *Irf7* and *Mki67* showed increased accessibility at ERV-overlapping enhancers and at Pu.1-binding sites (based on ^19^; Fig. 1o,p and Extended Data Fig. 1h), suggesting a role for Pu.1 in their regulation. *Spi1* (coding for Pu.1) regulatory elements showed increased opening but its expression was unaltered (Extended Data Fig. 1h).

In agreement with the reported role for Atrx in the regulation of the telomeric repeat-containing RNA (TERRA)^10,20–23^, TERRA-binding sites (TERRA-BS)^24^ were more open across all chromosomes in Daxx-KO LT-HSCs, including those at *Mid1* and *Erdr1*, a potential HSC regulator and repressor of inflammatory skin disease^25–27^ (Extended Data Fig. 1i,j). Chromatin opening correlated with their downregulation, as was also observed at the autosomal *Wls* locus (Extended Data Fig. 1j). TERRA knockdown also reduced expression of these loci, whereas Atrx loss increased their expression^23^, suggesting that Daxx and Atrx may antagonize each other for TERRA regulation.

Overall, our data suggest that Daxx loss in LT-HSCs alters the chromatin landscape at ERV-overlapping enhancers and TERRA-BS, with potential implications for induction of an IFN type I response and cell-cycle entry.

## Daxx loss skews haematopoiesis towards myeloid differentiation

**Fig. 2:**
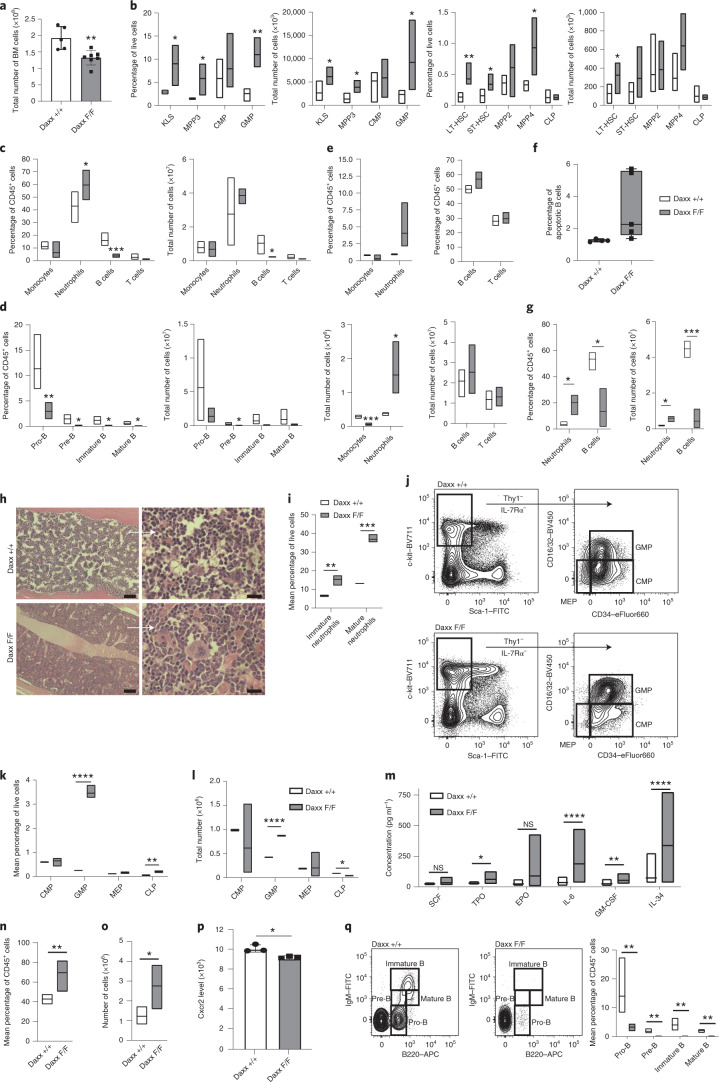
Acute and chronic Daxx loss lead to perturbations of haematopoiesis. **a**, Total number of cells (*n* = 5 WT and 7 Daxx-KO mice). **b**, Frequency and total cell number of stem, multipotent and progenitor populations (*n* = 4 WT and 5 Daxx-KO mice). **c**, Frequency and total cell number of mature lymphoid and myeloid cells(*n* = 6 WT and 7 Daxx-KO mice). **d**, Frequency and total cell numbers of B cell-progenitor populations (*n* = 4 WT and 5 Daxx-KO mice). **e**, Frequency and total counts of mature lymphoid and myeloid cells (*n* = 4 mice per genotype). **f**, Frequencies of apoptotic B cells (*n* = 4 WT and 5 Daxx-KO mice). **g**, Frequency and total cell numbers of B cells and neutrophils at 2 w.p.i. (*n* = 3 mice per genotype). **h**, Representative images of haematoxilin and eosin (H&E) staining of bones (*n* = 2 independent experiments). Scale bars, 100 µm (left) and 20 µm (right; higher-magnification images). **i**, Frequencies of immature and mature neutrophils (Gr1^+^CD11b^+^ cells; *n* = 3 mice per genotype). **j**, Examples of the flow cytometry analysis of myeloid progenitor cells. **k**,**l**, Frequency (**k**) and total count (**l**) of haematopoietic progenitors (*n* = 3 mice per genotype). **m**, Concentration of different cytokines in plasma (*n* = 6 mice per genotype; analysis of variance and corresponding non-parametric Conover’s test). TPO, thrombopoietin. **n**, Frequencies of neutrophils (*n* = 6 mice per genotype; non-parametric Mann–Whitney test). **o**, Number of neutrophils (*n* = 5 mice per genotype; non-parametric Mann–Whitney test). **p**, Cxcr2 levels measured in mature neutrophils (*n* = 3 mice per genotype). MFU, mean fluorescent units. **q**, Examples of flow cytometry analysis of B cell progenitors and frequencies of B cells and progenitors (right; *n* = 6 mice per genotype; non-parametric Mann–Whitney test). **a**–**g**, *RosaCreERT2* mice. **h**–**q**, *Mx1Cre* mice. **a**–**d**,**i**–**l**,**n**–**q**, BM. **e**–**g**, Spleen. **a**–**g**,**i**,**k**,**l**,**p**, Student’s *t*-test. **b**–**g**,**i**,**k**–**o**, Boxplots show the minimum and maximum values (box boundaries) and the mean (horizontal line). **a**,**p**, Data are the mean ± s.d. *P < 0.05, **P < 0.01, ***P < 0.001, ****P < 0.0001 and NS, not significant. Daxx F/F, Daxx KO and Daxx +/+, Daxx WT. Exact *P* values and numerical source data are provided. Source data

We next investigated the effect of Daxx loss on haematopoiesis. The overall cell numbers in the KO bone marrow (BM) were significantly reduced at 3 d.p.i. (Fig. 2a), whereas the frequencies and numbers of LT-HSCs, c-Kit^+^Lineage (Lin)^–^Sca-1^+^ (KLS) cells and myeloid-restricted multipotent progenitor (MPP) 3 cells (Fig. 2b) were increased. Both GMPs and lymphoid-biased MPP4 cells showed increased frequencies. The neutrophil frequency in the BM was augmented, whereas the frequency and number of B220^+^ cells were reduced (Fig. 2c). The B-cell defect encompassed pro-B, pre-B, and immature and mature B cells (Fig. 2d). The neutrophil frequency and number in the spleen were increased, whereas the monocyte number was reduced. B cells were unaffected and remained viable (Fig. 2e,f). At 2 w.p.i., the frequency and number of neutrophils were higher, whereas those of B cells were lower (Fig. 2g). Thus, Daxx loss causes early expansion of stem and progenitor cells, followed by increased BM and peripheral accumulation of neutrophils at the expense of B cells.

**Extended Data Fig. 2:**
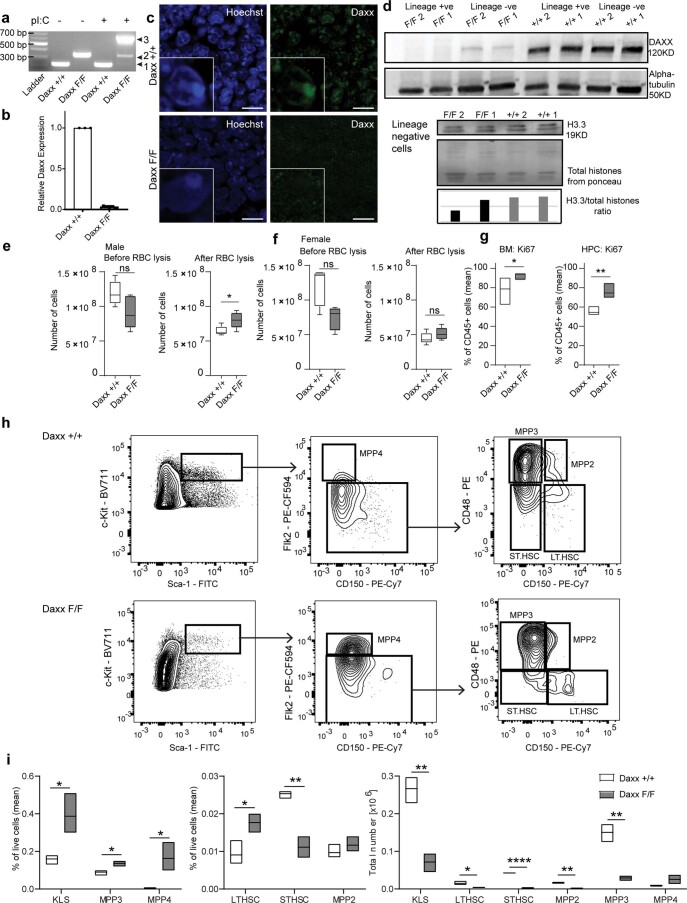
Daxx-deficient BM displays increased proliferation and altered stem/progenitor cell frequencies and numbers. **a**, Genotyping of *Daxx*^*+/+*^*;Mx1Cre*^*+/-*^ and *Daxx*^*F/F*^*;Mx1Cre*^*+/-*^ mice untreated (-) or treated with pI:pC (+). Arrows indicate the wild-type, floxed and recombined alleles (*n* = 5 independent experiments). **b**, Daxx mRNA levels upon pI:pC treatment in bone marrow. Shown is relative expression of Daxx mRNA over Tbp mRNA levels (*n* = 3 mice per genotype). **c**, IF of Daxx in bone marrow, scale bar = 10 µm (*n* = 4 mice per genotype, two independent experiments). Inlay image zoomed on representative nuclei. **d**, Western blot of Daxx, alpha-tubulin and H3.3 in Lineage-negative and Lineage-positive bone marrow cells (*n* = 2 mice per genotype). **e**, Bone marrow cellularity counts before and after RBC lysis in male mice (*n* = 5 mice per gender, non-parametric Mann–Whitney test, box-and-whiskers plot: min to max (whiskers), 25^th^ and 75^th^ percentile and median). **f**, Bone marrow cellularity counts before and after RBC lysis in female mice (*n* = 5 mice per gender, non-parametric Mann–Whitney test, box-and-whiskers plot: min to max (whiskers), 25^th^ and 75^th^ percentile and median). **g**, Flow cytometry analysis of Ki-67^+^ cells in BM and HPCs (*n* = 5 mice per genotype, repeated in two independent experiments, non-parametric Mann–Whitney test). **h,i**, Flow cytometry analysis of HSC and MPP populations (*n* = 3 mice per genotype, repeated in two independent experiments, Student’s t-test). BM, bone marrow; HPC, haematopoietic progenitor cells; HSC, haematopoietic stem cell; MPP, multipotent progenitors. Data in box plots are mean and min to max. ns, not significant; * *P* < 0.05, ** *P* < 0.01, *** *P* < 0.001, **** *P* < 0.0001. Exact p-values and numerical source data can be found in the accompanying source data. Unprocessed blots provided in Source data. Source data

**Extended Data Fig. 3:**
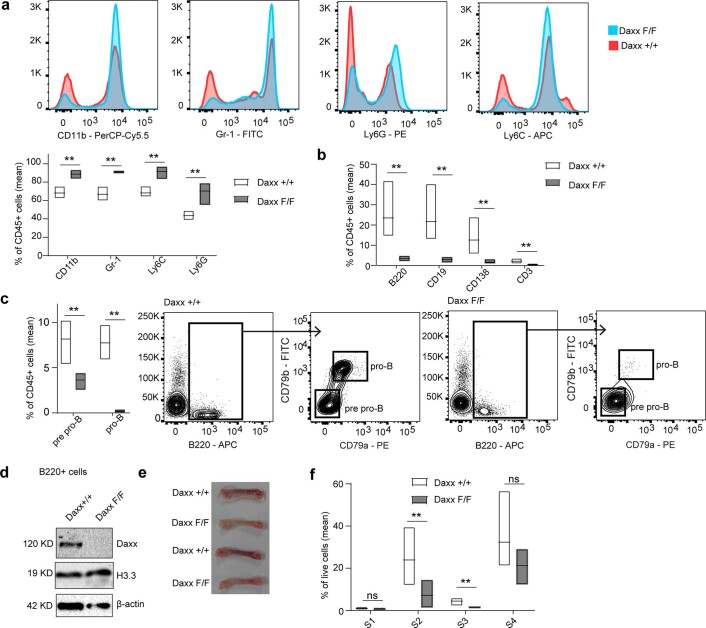
Daxx deficiency results in myeloid/lymphoid imbalance in the bone marrow. **a**, Flow cytometry analysis of myeloid cell surface markers (*n* = 6 mice per genotype, repeated in three independent experiments). **b**, Flow cytometry analysis of lymphoid cell surface markers (*n* = 6 mice per genotype, repeated in three independent experiments). **c**, Frequencies of B cell progenitors (*n* = 6 mice per genotype, repeated in three independent experiments. **d**, Western blot of Daxx, H3.3 and corresponding β-actin control in isolated B220^+^ cells from bone marrow (*n* = 3 mice per genotype, repeated in two independent experiments). **e**, Bones isolated from two *Daxx*^*+/+*^*;Mx1Cre* and *Daxx*^*F/F*^*;Mx1Cre* mice. **f**, Frequencies of erythroblast populations (*n* = 6 mice per genotype, repeated in three independent experiments. Data in box plots are mean and min to max. ns, not significant; * *P* < 0.05,d ** *P* < 0.01, *** *P* < 0.001, **** *P* < 0.0001, Wilcoxon rank test. Exact p-values and numerical source data can be found in the accompanying source data. Source image file provided in Source data. Source data

To study the long-term effects of Daxx loss on haematopoiesis, we employed haematopoiesis-specific Cre lines. Daxx deletion during prenatal haematopoiesis using the *Csf1rCre* line failed to produce viable Daxx-KO pups (not shown), similar to a germline Daxx-KO line^28^. Haematopoiesis-specific and polyinosinic:polycytidylic acid (pI:pC)-inducible Daxx deletion in adult mice (using the *Mx1Cre* line^29^; Extended Data Fig. 2a–d) resulted in an increase in mature and immature neutrophils in the BM (Fig. 2h,i). Daxx-deficient mice displayed a trend towards lower BM cell counts but the number of white blood cells was higher in males after red-blood-cell (RBC) lysis (Extended Data Fig. 2e,f). These changes correlated with a higher frequency of Ki67^+^ cells in the BM as well as in HSPCs (Extended Data Fig. 2g). Similar to the *RosaCre*^ERT2^ model, the GMP frequency and number were increased (Fig. 2j,k). Although the frequency of common lymphoid progenitors (CLPs) was augmented, their total number was reduced (Fig. 2k,l); similar observations were made for LT-HSCs, and KLS, MPP3 and MPP4 cells (Extended Data Fig. 2h,i). Cytokines promoting myeloid differentiation, such as granulocyte-macrophage colony-stimulating factor (GM-CSF), interleukin (IL)-6, thrombopoietin and IL-34, were increased in plasma (Fig. 2m), correlating with higher frequencies of CD11b^+^, Gr-1^hi^ and Ly6G^hi^ myeloid cells (Extended Data Fig. 3a). While Ly6C^hi^ inflammatory monocytes were nearly absent (Extended Data Fig. 3a), neutrophils displayed increased number and frequency as well as diminished expression of the maturation marker Cxcr2 (Fig. 2n–p). The B-lymphocyte frequencies were reduced, with a marked drop from the pre pro-B cell stage onward (Extended Data Fig. 3b,c and Fig. 2q). The levels of Daxx protein were reduced in B cells with Daxx KO (Extended Data Fig. 3d), suggesting that a small number of recombined B cells are produced. Finally, the paler appearance of the bones of the Daxx-KO animals correlated with reduced levels of RBCs and erythroblast subpopulations, suggesting an impairment in BM erythropoiesis (Extended Data Fig. 3e,f).

Overall, Daxx loss markedly caused perturbations of haematopoietic differentiation compatible with a myeloid bias towards neutrophil production.

## Daxx-KO mice develop neutrophilia and inflammation

**Fig. 3:**
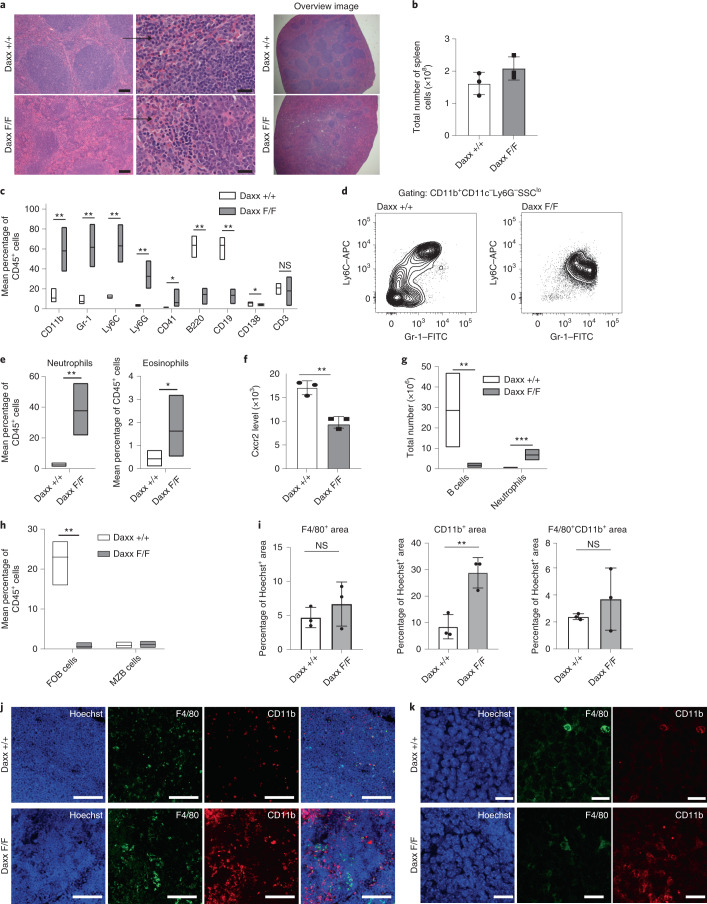
Daxx-deficient spleens display marked structural changes linked to an increase of neutrophils and reduction of B cells. **a**, Representative images of H&E-stained spleen sections (*n* = 3 independent experiments). Scale bars, 100 µm (left) and 20 µm (middle; higher-magnification images). Overview images of the section are shown (right). **b**, Total number of spleen cells (*n* = 3 mice per genotype). **c**, Flow cytometry analysis of cell surface markers (*n* = 6 mice per genotype). **d**, Flow cytometry plots showing macrophage- and monocyte-like populations. **e**, Frequencies of neutrophils (left) and eosinophils (right; *n* = 5 mice per genotype). **f**, Cxcr2 levels in mature neutrophils of the spleen (*n* = 3 mice per genotype). MFU, mean fluorescent units. **g**, Total B-cell and neutrophil counts in the spleen (*n* = 5 mice per genotype). **h**, Frequencies of follicular and marginal-zone B cells (FOB and MZB, respectively; *n* = 6 mice per genotype). **i**, Levels of F4/80 (left), CD11b (middle) and combined F4/80 and CD11b (right) fluorescence (*n* = 3 mice per genotype). **j**, Immunofluorescence images of spleen sections (*n* = 3 mice per genotype). Scale bars, 100 µm. **k**, Magnified views of the images in **j**. Scale bars, 15 µm. **c**,**e**,**g**,**h**, Boxplots show the minimum and maximum values (box boundaries) and the mean (horizontal line). **b**,**f**,**i**, Data are the mean ± s.d. *P < 0.05, **P < 0.01 and NS, not significant; Student’s *t*-test (**b**,**f**,**g**,**i**) or non-parametric Wilcoxon rank test (**c**,**e**,**h**). Daxx F/F, Daxx KO and Daxx +/+, Daxx WT. Exact *P* values and numerical source data are provided. Source data

**Extended Data Fig. 4:**
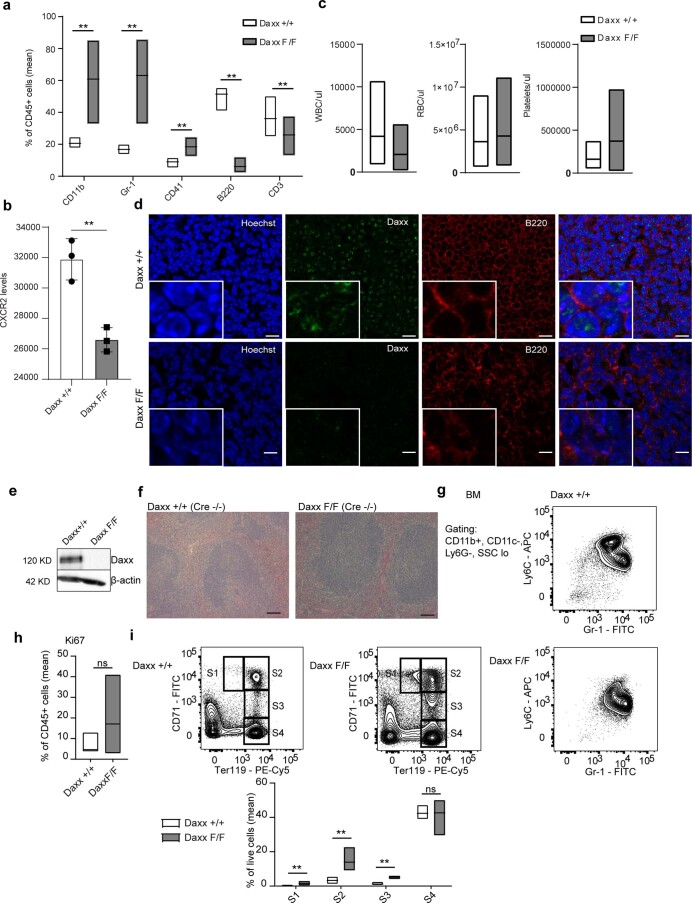
Loss of Daxx leads to neutrophilia and B-cell lymphopenia in the periphery. **a**, Flow cytometry analysis of peripheral blood collected 4 weeks post induction (*n* = 6 mice per genotype, repeated in three independent experiments). **b**, Cxcr2 levels in mature neutrophils of the blood (*n* = 3 mice per genotype, Student’s t-test). **c**, Quantification of WBC, RBC and platelets using Sysmex (*n* = 3 mice per genotype). **d**, IF images of spleen stained for Daxx and B220 (*n* = 2 mice per genotype, repeated in two independent experiments). Scale bar indicates 10 µm. **e**, Western Blot of Daxx protein in spleen (*n* = 3 mice per genotype, repeated in three independent experiments). **f**, H&E stain of spleen sections in Cre-negative animals, scale bar = 100 µm (*n* = 2 mice per genotype). **g**, Representative flow cytometry plots showing Gr-1^+^/Ly6C^+^ populations in bone marrow upon CD11b^+^, CD11c^-^, Ly6G^−^, SSC^lo^ gating. **h**, Frequencies of Ki67^+^ cells in spleen (*n* = 5 mice per genotype, repeated in two independent experiments). **i**, Frequencies of erythroblast populations in spleen (*n* = 6 mice per genotype, repeated in three independent experiments). Data in box plots are mean and min to max. ns, not significant; * *P* < 0.05, ** *P* < 0.01, *** *P* < 0.001, **** *P* < 0.0001, Wilcoxon rank test if not otherwise noted. Exact p-values and numerical source data can be found in the accompanying source data. Source image file provided in Source data. Source data

Myeloid markers were increased in the peripheral blood (PB) of Daxx-KO mice, whereas the B-cell frequency decreased (Extended Data Fig. 4a). The levels of Cxcr2 were reduced in PB neutrophils, indicating a more immature status also outside of the BM (Extended Data Fig. 4b). A trend towards a reduction in the white-blood-cell count was observed (Extended Data Fig. 4c). Compared with controls, Daxx loss (Extended Data Fig. 4d–f) led to marked reduction in white pulp (Fig. 3a). The total number of spleen cells was unchanged, despite an increased frequency of myeloid cells (Fig. 3b,c). The CD41^+^ megakaryocyte frequency was higher (Extended Data Fig. 4a), although the number of platelets in the PB was only marginally increased (Extended Data Fig. 4c). Correlating with the reduction in white pulp, B cells were significantly reduced (Fig. 3c,g); the decrease affected follicular but not marginal B cells and to a minor degree plasma cells (Fig. 3c,h). Spleen macrophages (Ly6C^lo/−^Gr-1^lo/−^) were reduced, whereas monocytes were shifted towards a Ly6C^intm^Gr-1^hi^ population (Fig. 3d and Extended Data Fig. 4g). We observed an increase in the neutrophil number and frequency, a higher eosinophil frequency, decreased Cxcr2 levels (Fig. 3e,f) and a trend towards an increased Ki67^+^ cell frequency (Extended Data Fig. 4h). Expansion of CD11b^+^ cells was observed but the F4/80^+^ monocytes/macrophages were unaltered (Fig. 3i–k). In contrast to BM, erythroblast subpopulations were increased (Extended Data Fig. 4i), suggesting induction of extramedullary erythropoiesis, in accordance with normal RBC counts in the PB.

**Fig. 4:**
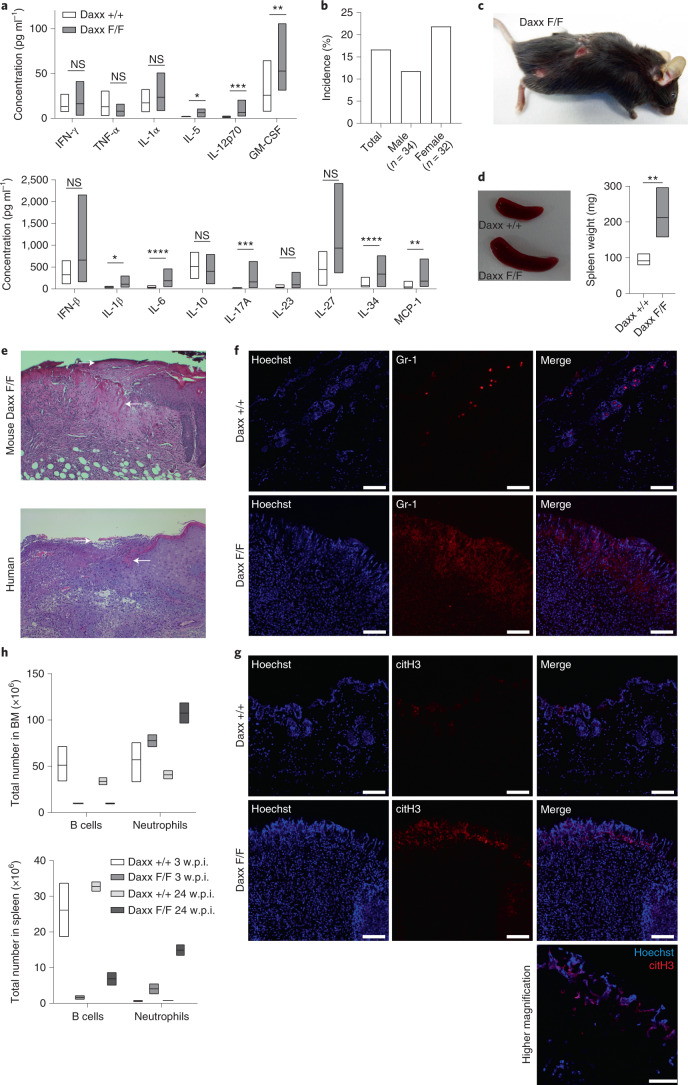
Chronic Daxx loss leads to increased inflammatory cytokines and pyoderma gangrenosum, an autoinflammatory neutrophilic disease. **a**, Concentrations of cytokines in plasma (*n* = 6 mice per genotype). **b**, Incidence of skin lesions in the mice in the total group and stratified by sex. Data are the mean. **c**, *Daxx*^F/F^;*Mx1Cre*^+/−^ mouse with skin lesions. **d**, Representative image of the spleens of control and Daxx-KO mice with skin lesions (left), and the spleen weight of mice with skin lesions (right; *n* = 6 mice per genotype). **e**, Skin lesion of a *Daxx*^F/F^;*Mx1Cre*^+/−^ mouse (top; *n* = 3 biological replicates) compared with a human skin biopsy of a patient with pyoderma gangrenosum (bottom). Representative images of H&E-stained sections; the arrows indicate the site of the ulcer and fibrin layer. **f**, Immunofluorescence staining of Gr-1^+^ cells in sections of skin lesions (*n* = 2 independent experiments). Scale bars, 100 µm. **g**, Immunofluorescence staining of sections of skin lesions with citH3 (*n* = 2 independent experiments). The higher-magnification image (bottom) shows NETosis in a *Daxx*^F/F^;*Mx1Cre*^+/−^ skin lesion. Scale bars, 100 µm and 20 µm (higher-magnification image). **h**, Number of B cells and neutrophils in the BM (top) and spleen (bottom) at 3 and 24 w.p.i. (*n* = 2 mice per genotype, except for WT at 3 w.p.i., where *n* = 4 mice). **a**,**h**, Boxplots show the minimum and maximum values (box boundaries) and the mean (horizontal line). *P < 0.05, **P < 0.01, ***P < 0.001, ****P < 0.0001 and NS, not significant; non-parametric Wilcoxon rank test. Daxx F/F, Daxx KO and Daxx +/+, Daxx WT. Exact *P* values and numerical source data are provided. Source data

In agreement with reports linking neutrophilia and inflammation^30–33^, we observed elevated levels of several key cytokines involved in myelopoiesis and inflammation (Fig. 4a), such as GM-CSF^34^ and IL-17A, the latter of which is linked to skin diseases^35,36^. Interestingly, approximately 15% of the *Daxx*^F/F^;*Mx1Cre*^+/−^ mice developed skin lesions along with splenomegaly (Fig. 4b–e). These lesions resembled human pyoderma gangrenosum, a cutaneous neutrophilic autoinflammatory disease of unknown aetiology (Fig. 4e)^37^ partly associated with myeloproliferative diseases. Similar to human pyoderma gangrenosum, the mouse lesions were characterized by thicker skin and sterile inflammation (Fig. 4e), with infiltration of neutrophils and neutrophil extracellular traps (Fig. 4f,g). The rather long latency of skin lesions correlated with a progressive increase in neutrophilia over time (Fig. 4h).

These findings suggest that progressive neutrophilia leads to systemic inflammation in Daxx-deficient mice.

## Limited role of Hira in adult haematopoiesis

**Extended Data Fig. 5:**
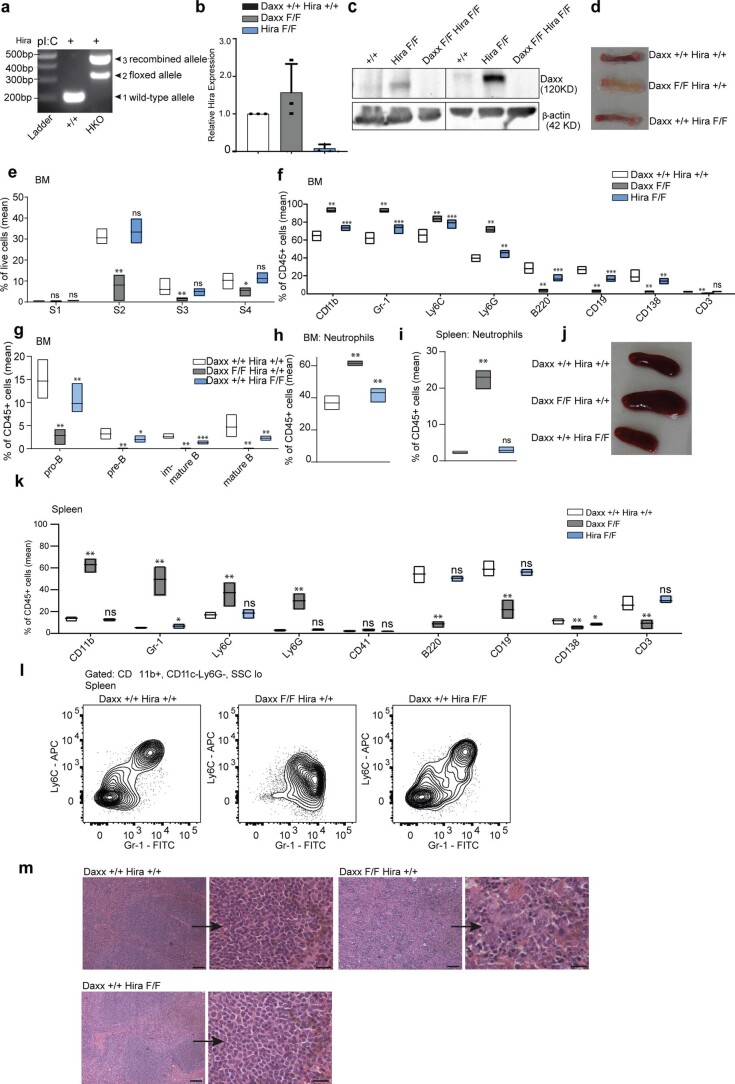
Loss of the H3.3 chaperone Hira has marginal effects on haematopoiesis. **a**, Representative images of genotyping results using primers to detect WT, floxed and recombined bands of Hira in bone marrow cells (*n* = 3 independent experiments). **b**, Hira mRNA levels upon pI:pC treatment in bone marrow cells. Shown is relative expression of Hira mRNA over Tbp mRNA levels (*n* = 3 mice per genotype). **c**, Representative Western blots for Daxx and β-Actin in total bone marrow of Daxx ^+/+^, Hira ^F/F^ and Daxx/Hira double KO Mx1Cre mice (*n* = 3 mice per genotype, repeated in two independent experiments). **d**, Bones of WT, Daxx KO and Hira KO mice. **e**, Frequencies of erythroblast populations in bone marrow (WT mice *n* = 9; Daxx KO *n* = 3; Hira KO *n* = 6). **f**, Flow cytometry analysis of mature cell markers in bone marrow (WT mice *n* = 9; Daxx KO *n* = 3; Hira KO *n* = 6). **g**, Flow cytometry analysis of B cells in bone marrow (WT mice n = 9; Daxx KO n = 3; Hira KO n = 6). **h**, Frequencies of neutrophils in bone marrow (WT mice *n* = 9; DKO *n* = 3; HKO *n* = 6). **i**, Frequencies of neutrophils in spleen (WT mice n = 9; Daxx KO n = 3; Hira KO *n* = 6). **j**, Spleens isolated from WT, Daxx KO and Hira KO mice. **k**, Flow cytometry analysis of mature cells in spleen (WT mice *n* = 9; Daxx KO *n* = 3; Hira KO *n* = 6). **l**, Flow cytometry plots gated on monocyte- and macrophage-like populations in spleen. **m**, H&E stain of spleen sections (*n* = 3 mice per genotype), scale bar = 100 µm. Higher magnification, scale bar = 20 µm. Data produced 3 weeks post pI:pC treatment. Data in box plots are mean and min to max. ns, not significant; * *P* < 0.05, ** *P* < 0.01, *** *P* < 0.001, **** *P* < 0.0001, Wilcoxon rank test. Exact p-values and numerical source data can be found in the accompanying source data. Source image file provided in Source data. Source data

We next assessed the contribution of Histone cell-cycle regulator (Hira), which deposits H3.3 at euchromatin and facultative heterochromatin^38^. In pI:pC-treated *Hira*^F/F^;*Mx1Cre*^+/−^ mice (Extended Data Fig. 5a–c), no changes in erythroid marker frequencies or bone appearance were observed (Extended Data Fig. 5d,e). The frequencies of myeloid and lymphoid markers in the BM were significantly affected but markedly less than that observed in the Daxx-KO mice (Extended Data Fig. 5f,g). Although the frequency of neutrophils was significantly increased in the BM of the Hira-KO mice, it was unaffected in the spleen (Extended Data Fig. 5h,i). Unlike Daxx-deficient mice, the spleens of the Hira-KO mice were not enlarged (Extended Data Fig. 5j) and showed no or only minor changes in the myeloid, erythroid and lymphoid marker frequencies as well as organ structure (Extended Data Fig. 5k–m). No skin lesions were observed after extended monitoring. The marginal effects of Hira loss on haematopoiesis may be due to its lesser role and/or compensatory effects by Daxx given that the Daxx levels were higher in Hira-deficient cells (Extended Data Fig. 5c). The stronger phenotype described in a previous report based on Hira prenatal deletion^39^ could be caused by a more prominent Hira role during embryonic haematopoiesis.

## Haematopoietic defects of Daxx deficiency are cell-intrinsic

**Extended Data Fig. 6:**
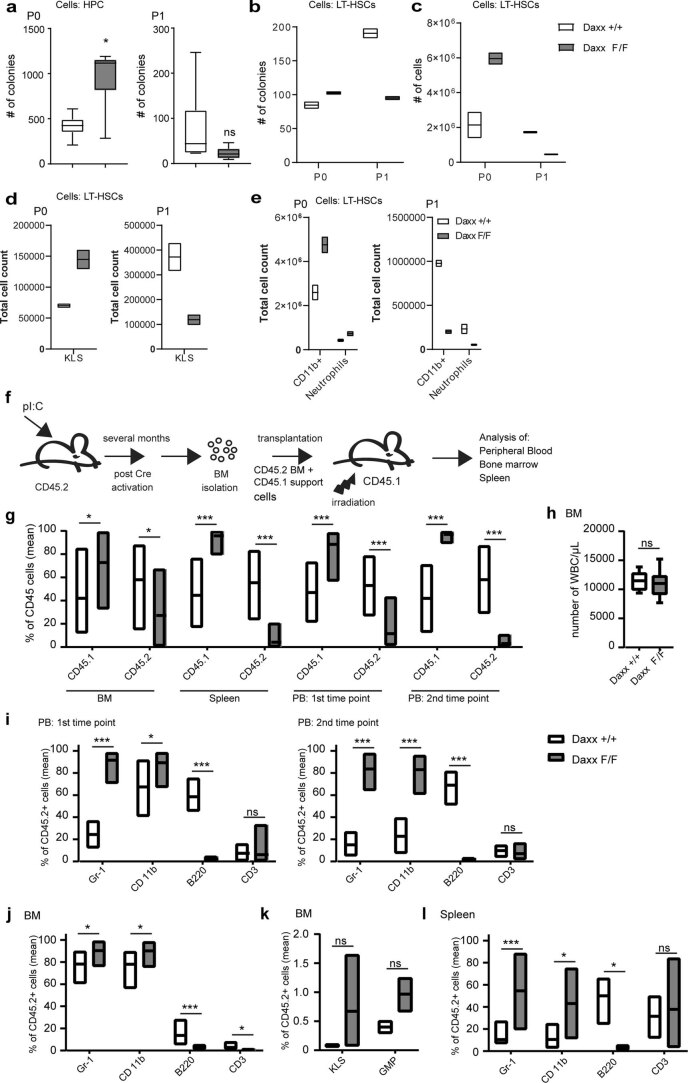
The haematopoietic phenotype of Daxx deletion is due to a cell-intrinsic defect. **a**, Number of colonies after first plating (P0) and first passage (P1) using haematopoietic progenitor cells (*n* = 5 mice per genotype, repeated in three independent experiments, box-and-whiskers plot: min to max, 25^th^ to 75^th^ percentile and median). **b**, Number of colonies after first plating (P0) and first passage (P1) using LT-HSCs (*n* = 2 mice per genotype). **c**, Number of cells after first plating (P0) and first passage (P1) using LT-HSCs (*n* = 2 mice per genotype). **d**, Number of KLS cells and **e**, number of CD11b + cells and neutrophil-like cells (Ly6C + ,Ly6G + ) after first plating (P0) of LT-HSCs and first passage (P1; *n* = 2 mice per genotype). **f**, Schematic representation of the experimental set-up to generate bone marrow chimeras. **g**, Frequencies of CD45.1^+^ and CD45.2^+^ cells in transplanted mice (*n* = 8 mice per genotype). **h**, WBC counts in total bone marrow (*n* = 8 mice per genotype, box-and-whiskers plot: min to max, 25^th^ to 75^th^ percentile and median). **i**, Flow cytometry analysis in peripheral blood of transplanted mice at two different time points: 1^st^: 4-5 weeks post transplantation; 2^nd^ pooled data from two independent experiments collected at 8 or 12 weeks post transplantation (*n* = 8 mice per genotype). **j**, Frequencies of mature CD45.2^+^ blood cells in bone marrow of recipient mice (*n* = 8 mice per genotype, repeated in three independent experiments). **k**, Frequencies of CD45.2^+^ KLS and GMPs in bone marrow (*n* = 4 mice per genotype, repeated in two independent experiments). **l**, Flow cytometry analysis of CD45.2^+^ mature blood cells in spleen of recipient mice (*n* = 8 mice per genotype, repeated in three independent experiments). BM, bone marrow; PB, peripheral blood; KLS, c-Kit^pos^, lineage^neg^, Sca-1^pos^; GMP, granulocyte-monocyte progenitor; P0, first plating; P1, first passage. Data in box plots are mean and min to max. ns, not significant; * *P* < 0.05, ** *P* < 0.01, *** *P* < 0.001, **** *P* < 0.0001, Wilcoxon rank test. Exact p-values and numerical source data can be found in the accompanying source data. Source data

To determine the nature of the phenotypic changes described earlier, we performed colony-forming unit (CFU) assays using either Lin^−^ progenitors (HPCs) or LT-HSCs from untreated *Daxx*^F/F^;*Mx1Cre*^+/−^ and *Daxx*^+/+^;*Mx1Cre*^+/−^ mice, which underwent spontaneous recombination in vitro, probably due to activation of IFN signalling (not shown). An increase in colonies was observed following Daxx loss in HPCs (Extended Data Fig. 6a) at passage 0 (P0). Conversely, at P1 Daxx-deficient cells gave rise to fewer colonies, suggesting that their colony-forming potential was reduced. The CFU assays with LT-HSCs showed a similar phenotype following Daxx loss (Extended Data Fig. 6b,c), with an increased number of KLS cells at P0 and reduction at P1 (Extended Data Fig. 6d). Similar changes were observed in the number of CD11b^+^ cells and neutrophils (Extended Data Fig. 6e).

Finally, transplantation of BM cells isolated from pI:pC-treated mice into congenic recipients (Extended Data Fig. 6f) suggested a reduced reconstitution capacity of Daxx-KO stem/progenitor cells, resulting in a low frequency of CD45.2^+^ cells in the periphery (Extended Data Fig. 6g). Despite similar white-blood-cell counts (Extended Data Fig. 6h), KO transplants displayed altered frequencies of myeloid and B cells in the BM, PB and spleen, and of KLS cells and GMPs in the BM, as observed in the donor mice (Extended Data Fig. 6i–l).

Overall, these data suggest that the aberrant blood-cell composition observed following Daxx loss is due to a cell-intrinsic defect.

## Pu.1-driven myeloid signature following Daxx loss

**Fig. 5:**
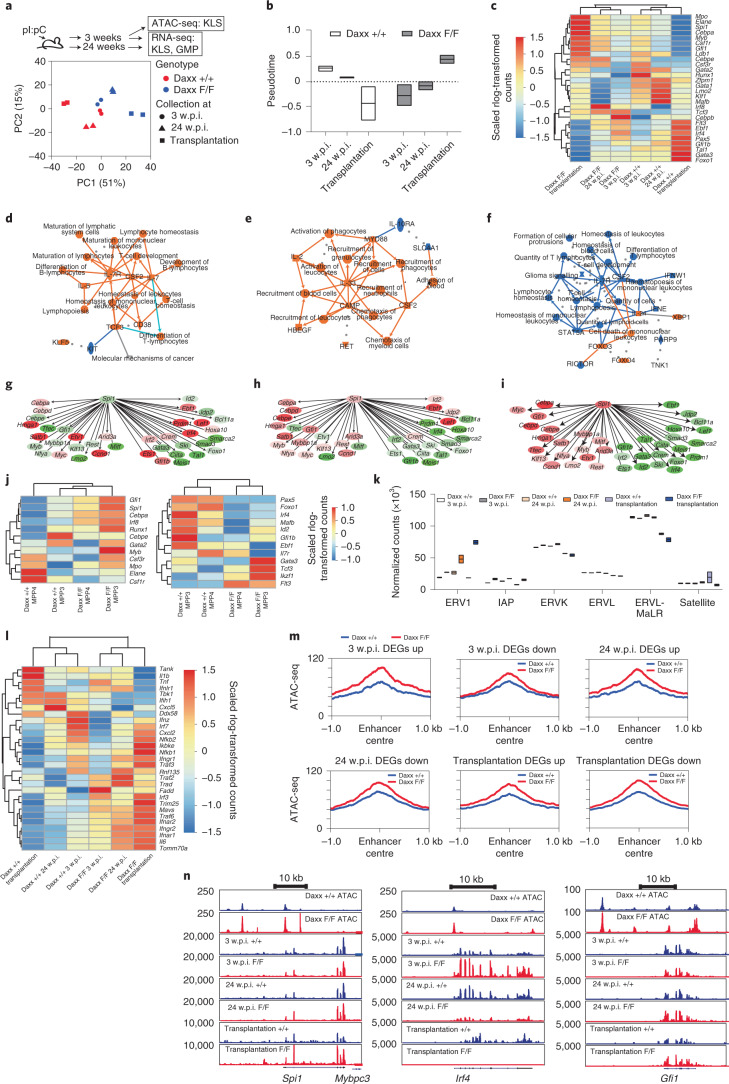
Daxx loss affects the transcriptome of haematopoietic progenitors in the context of both steady-state and stress haematopoiesis. RNA-seq analysis of *Daxx*^+/+^;*Mx1Cre*^+/−^ and *Daxx*^F/F^;*Mx1Cre*^+/−^ KLS cells isolated at 3 and 24 w.p.i. or from transplanted animals. **a**, Experimental scheme for cells isolated from pI:pC-treated mice (top) and PCA of the top-500 most variable genes (bottom). **b**, Mean pseudotime for each sample group (*n* = 2 mice per genotype). **c**, Heatmap showing scaled expression of selected transcription factors and regulators involved in blood-cell differentiation (*n* = 2 mice per genotype). **d**, Summary plot of IPA analysis of DEGs at 3 w.p.i. **e**, Summary plot of IPA analysis of DEGs at 24 w.p.i. **f**, Summary plot of IPA analysis of DEGs in KLS cells from transplanted animals. **d**–**f**, Predicted activation of protein or biofunction is indicated in orange and predicted inhibition of the displayed protein or biofunction in dark and light blue, respectively. Grey dotted lines are machine learning-based inferred connections. **g**, Expression changes, at 3 w.p.i., of the transcription factor genes regulated by Pu.1. **h**, Expression changes, at 24 w.p.i., of transcription factor genes regulated by Pu.1. **i**, Expression changes of the transcription factor genes regulated by Pu.1 in KLS cells isolated from transplanted mice. **g**–**i**, Upregulated genes are shown in red and downregulated genes in green. A stronger colour intensity indicates higher absolute log_2_-transformed fold changes. **j**, Heatmaps showing myeloid- or lymphoid-driving transcription factors for MPP3 and MPP4 cells. **k**, Normalized read counts of ERV/RTE subtypes and satellite repeats (*n* = 2 mice per genotype). **l**, Heatmap showing expression of interferon-responsive genes and dsRNA-recognition machinery. **m**, ATAC-seq read coverage at enhancers overlapping ERVs close to genes that are up- or downregulated in KLS cells collected at 3 and 24 w.p.i. or from transplanted animals. **n**, Genome browser tracks of the regulatory regions of *Spi1*, *Irf4* and *Gfi1* showing ATAC-seq and RNA-seq coverage. Transcripts of genes of the depicted region are shown below. **b**,**k**, Boxplots show the minimum and maximum values (box boundaries) and the mean (horizontal line). Daxx F/F, Daxx KO and Daxx +/+, Daxx WT. Numerical source data provided. Source data

**Extended Data Fig. 7:**
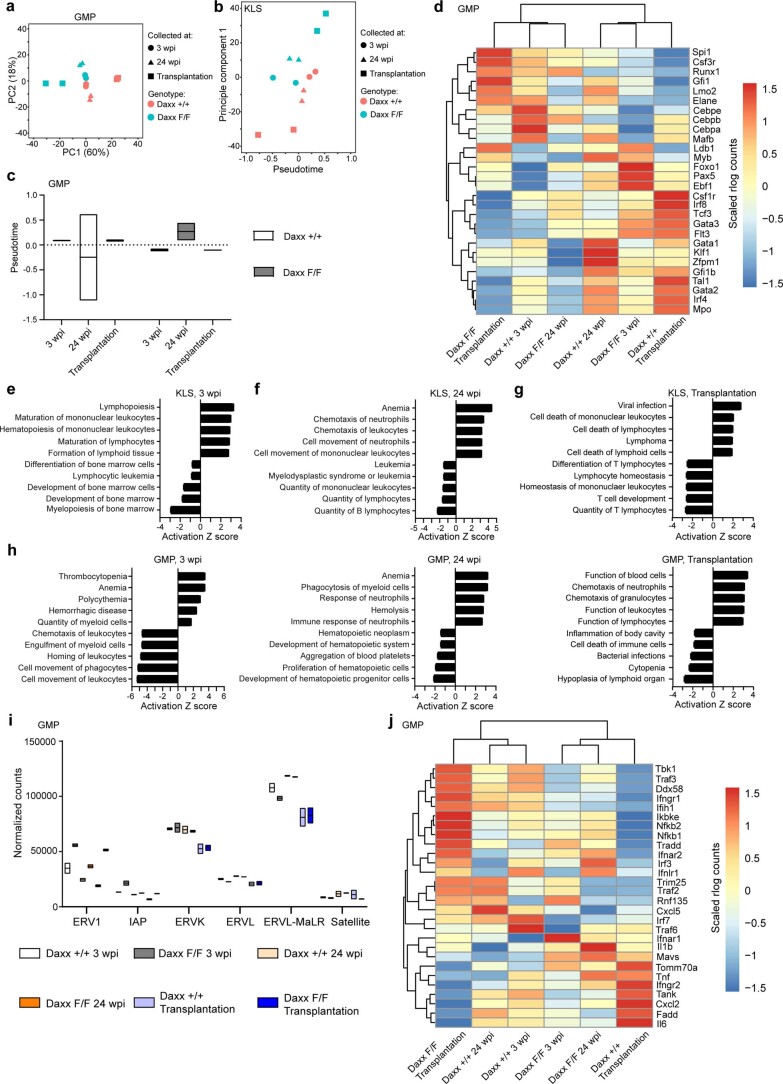
Gene expression changes in Daxx-deficient haematopoietic progenitors. **a**, PCA of the top-500 most variable genes for GMPs. **b**, Trajectory of gene expression of KLS collected at 3 w.p.i., 24 w.p.i. or from transplanted animals. **c**, Boxplot showing mean pseudotime for each sample group for GMPs (*n* = 2 mice per genotype). **d**, Heatmap showing scaled expression of selected transcription factors and regulators involved in blood cell differentiation (*n* = 2 mice per genotype). **e**, Top 5 haematologic biofunctions and diseases altered at 3 w.p.i. in KLS Daxx KO cells. **f**, Top 5 haematologic biofunctions and diseases altered at 24 w.p.i. in KLS Daxx KO cells. **g**, Top 5 haematologic biofunctions and diseases altered in KLS Daxx KO cells isolated from transplanted animals. **h**, Top 5 haematologic biofunctions and diseases altered in GMP Daxx KO cells collected at 3 w.p.i., 24 w.p.i. and from transplanted animals. **i**, Boxplots of mean normalized read counts of ERV subtypes and satellite repeats (*n* = 2 mice per genotype) in GMP cells. **j**, Heatmap showing scaled expression of interferon response genes and the dsRNA recognition machinery for GMPs. Data in box plots are min to max and mean. Numerical source data can be found in the accompanying source data. Source data

We performed transcriptome analyses of KLS and GMP subpopulations isolated from BM chimaeras (see the ‘Haematopoietic defects of Daxx deficiency are cell-intrinsic’ section; stress haematopoiesis) as well as from pI:pC-treated mice collected at 3 and 24 w.p.i. (Fig. 5a and Supplementary Tables 3–8; steady-state haematopoiesis). A PCA analysis revealed clustering according to genotype (Fig. 5a and Extended Data Fig. 7a) and that differences increased with time and following stress. When plotting the samples along pseudotime, Daxx-KO KLS cells followed a different trajectory, whereas KO GMPs did not (Fig. 5b and Extended Data Fig. 7b,c). Unsupervised clustering of selected master regulators and haematopoiesis markers revealed clustering according to genotype and condition as well as increased expression with time of myeloid-associated genes such as *Mpo*, *Elane*, *Spi1* and *Cebpa*. This was accompanied by a reduction in the expression levels of lymphoid genes (*Flt3*, *Ebf1*, *Irf4*, *Pax5* and *Gfi1b*) in the KO KLS cells (Fig. 5c) but not in GMPs (Extended Data Fig. 7d). Accordingly, by comparing cells at 3 and 24 w.p.i. we found that Daxx-KO KLS cells switch from pathways compatible with increased lymphopoiesis to increased myelopoiesis and reduced B-cell generation (Fig. 5d,e and Extended Data Fig. 7e,f). This effect was even more pronounced in transplantation settings (Fig. 5f and Extended Data Fig. 7g). Furthermore, an increasing number of Pu.1-target genes driving myeloid fate were upregulated over time and following stress, accompanied by reduced expression of lymphoid Pu.1-target genes (Fig. 5g–i). These data are in accordance with neutrophilia becoming more pronounced with time (Fig. 4h), which was also confirmed by pathway analysis for Daxx-KO GMP cells (Extended Data Fig. 7h).

Transcriptomics of lymphoid- and myeloid-primed multipotent progenitors (MPP4 and MPP3, respectively) combined with unsupervised clustering of myeloid marker gene and transcription factor expression showed that Daxx-KO MPP4 cells cluster together with WT MPP3 (Fig. 5j, top). A similar analysis based on lymphoid genes showed Daxx-KO MPP4 clustering with MPP3 cells, suggesting that Daxx-KO lymphoid-biased MPP4 have shifted towards a more myeloid-biased gene expression (Fig. 5j, bottom).

**Extended Data Fig. 8:**
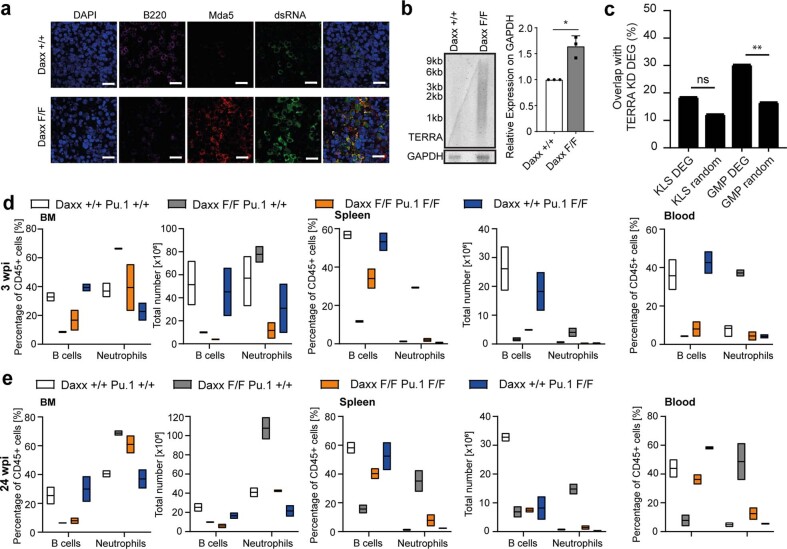
Changes in the RNA-sensing machinery and TERRA expression following Daxx loss. **a**, IF images of spleens stained for B220, Mda5 and dsRNA (*n* = 2 independent experiments), scale bar = 20 µm. **b**, Northern blot autoradiograph showing TERRA levels and quantification analysis (*n* = 3 mice per genotype, unpaired t-test with Welch’s correction). **c**, Barplot showing the overlap of differentially expressed genes in TERRA knockdown cells with DEG in our KLS and GMP Daxx KO datasets (transplantation) compared to random genes (Fisher’s Exact Test; **p-value < 0.01, ns, not significant). **d**, Quantification of B cells and neutrophils in bone marrow, spleen and peripheral blood at 3 weeks post induction (*n* = 2 mice per genotype). **e**, Quantification of B cells and neutrophils in bone marrow, spleen and peripheral blood at 24 weeks post induction (*n* = 2 mice per genotype). Data in box plots are min to max and mean. Exact p-values and numerical source data can be found in the accompanying source data. Source data

Deregulation of ERVs/RTEs in Daxx-KO KLS cells and GMPs became more pronounced with time and following stress, especially that of ERV1 and IAPs (Fig. 5k and Extended Data Fig. 7i). These changes correlated with induction of ISGs and RNA-sensing factors in both Daxx-KO KLS and GMPs, mostly at 24 w.p.i., and in transplanted cells (Fig. 5l and Extended Data Fig. 7j). Spleen staining showed increased levels of the retinoic acid-inducible gene I-pathway (RIG-I) component melanoma differentiation-associated gene 5 (Mda5) and double-stranded RNA (dsRNA; Extended Data Fig. 8a). Furthermore, TERRA long-noncoding RNA (lncRNA) was upregulated in Daxx-KO BM cells (Extended Data Fig. 8b) and there was significant overlap between differentially expressed genes (DEGs) from TERRA knockdown in embryonic stem cells^23^ with DEGs in Daxx-KO versus WT GMP datasets (Extended Data Fig. 8c).

Finally, ERV-overlapping enhancers in proximity of both up- and downregulated genes were more accessible in Daxx-KO KLS cells at 3 w.p.i. (Fig. 5m,n). For instance, *Spi1* and *Gfi1* (which together drive neutrophilic differentiation^40^) displayed increased enhancer accessibility at 3 w.p.i. but were upregulated only in transplantation settings. In contrast, *Irf4* expression (which cooperates with Pu.1 for B-cell differentiation^41^) was higher at 3 w.p.i. but lower at 24 w.p.i. and in transplantation settings.

## Daxx loss alters H3.3, Pu.1 and histone-mark distribution

**Fig. 6:**
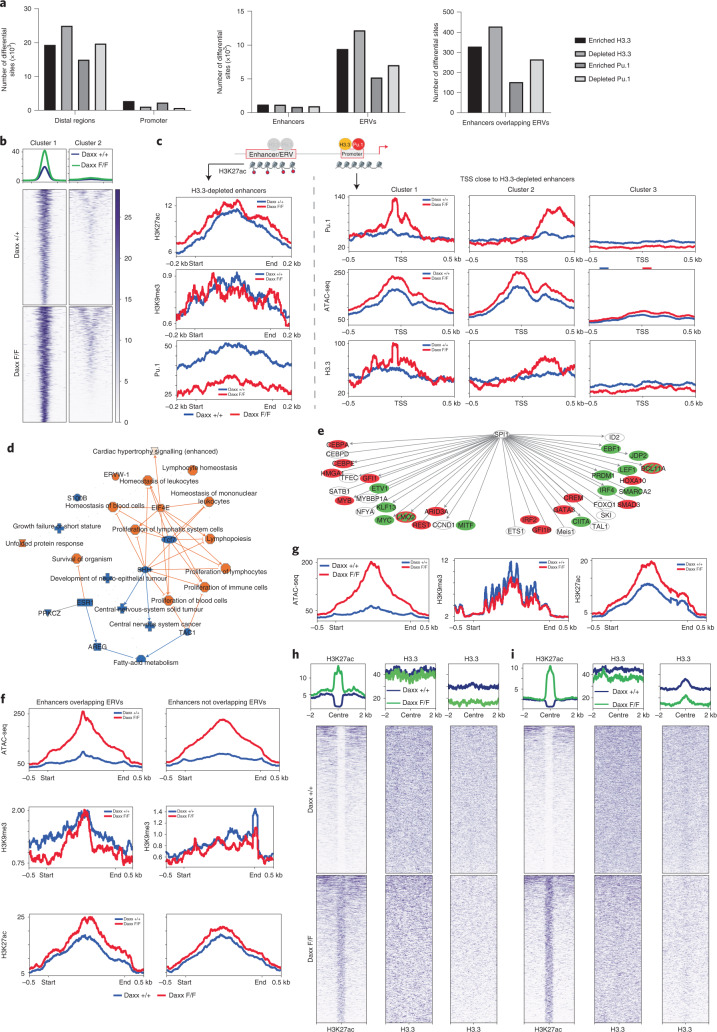
Daxx-deficient progenitors display alterations in H3.3 and Pu.1 genome-wide distribution as well as epigenetic marks. **a**, Overview of altered H3.3 distribution and Pu.1 binding in Daxx-KO HSPCs determined by CUT&Tag assays. **b**, Heatmaps of ATAC-seq read distribution around the centre of H3.3-depleted enhancers. **c**, H3K27ac, H3K9me3 and Pu.1 CUT&Tag read distribution across H3.3-depleted enhancers (left) as well as Pu.1, ATAC-seq and H3.3 coverage around the TSS of genes close to H3.3-depleted enhancers (right). Pu.1 coverage was stratified into three clusters by *k*-means and ATAC-seq read distribution was plotted for the same clusters of TSS. **d**, Summary plot of IPA analysis for genes close to distal regions with altered Pu.1 binding. Predicted activation of the protein or biofunction is indicated in orange and predicted inhibition of the displayed protein or biofunction in blue. **e**, Graphical depiction of Pu.1-binding changes at distal or proximal sites close to transcription factors regulated by Pu.1. The regions with increased Pu.1 binding are shown in red and regions with decreased Pu.1 binding in green. **f**, Enrichment plots for ATAC-seq, H3K9me3 CUT&Tag and H3K27ac CUT&Tag at enhancers overlapping and not overlapping ERVs with increased accessibility. **g**, Enrichment plots for ATAC-seq, H3K9me3 CUT&Tag and H3K27ac CUT&Tag at ERVs with increased accessibility. **h**, Heatmaps of enhancers with increased H3K27ac showing cluster analysis with H3.3. **i**, Heatmaps of ERVs with increased H3K27ac showing cluster analysis with H3.3. **h**,**i**, The legend for the heatmaps is the same as **b** and middle graphs show cluster of enhancers or ERVs/RTEs with no difference in H3.3 while right graphs show those with reduced H3.3 loading. Daxx F/F, Daxx KO and Daxx +/+, Daxx WT. Numerical source data are provided. Source data

We next performed low-input chromatin immunoprecipitation combined with sequencing using Cleavage Under Targets and Tagmentation (CUT&Tag) for H3.3, Pu.1, H3 lysine 27 acetylation (H3K27ac) and H3K9 trimethylation (H3K9me3) on WT and Daxx-KO HSPCs at 3 w.p.i. Daxx loss led to significant changes in H3.3 distribution and Pu.1 binding (Fig. 6a). Reduction in both H3.3 and Pu.1 at distal regions, particularly at ERVs, was accompanied by enrichment of both proteins at promoters (Fig. 6a), suggesting that their respective chromatin associations may be linked. H3.3-depleted enhancers were characterized by increased chromatin accessibility and H3K27ac but decreased Pu.1 levels (Fig. 6b,c). In turn, nearby genes showed increased Pu.1 binding around and downstream of the transcription start site (TSS), coinciding with increased chromatin accessibility and H3.3 enrichment (Fig. 6c). Pathway analysis for genes close to distal regions with altered Pu.1 binding showed enrichment of immune-cell categories (Fig. 6d). In addition, altered Pu.1 binding at Pu.1-regulated transcription factors (Fig. 6e) corresponded to changes in their gene expression (Fig. 5g).

Enhancers overlapping with ERVs/RTEs showed stronger depletion of H3K9me3 and increase in H3K27ac than other enhancers (Fig. 6f), despite a similar increase in chromatin accessibility. Generally, ERVs/RTEs showing increased accessibility were enriched in H3K27ac but displayed minor changes in H3K9me3 (Fig. 6g). Finally, among enhancers (Fig. 6h) and ERVs/RTEs gaining H3K27ac (Fig. 6i), one subset displayed clear reduction in H3.3 following Daxx loss.

Together, these findings suggest that Daxx loss alters chromatin features and Pu.1 distribution at enhancers, including those overlapping with ERVs.

## Phenotypic rescue and unique changes after Daxx and Pu.1 DKO

**Fig. 7:**
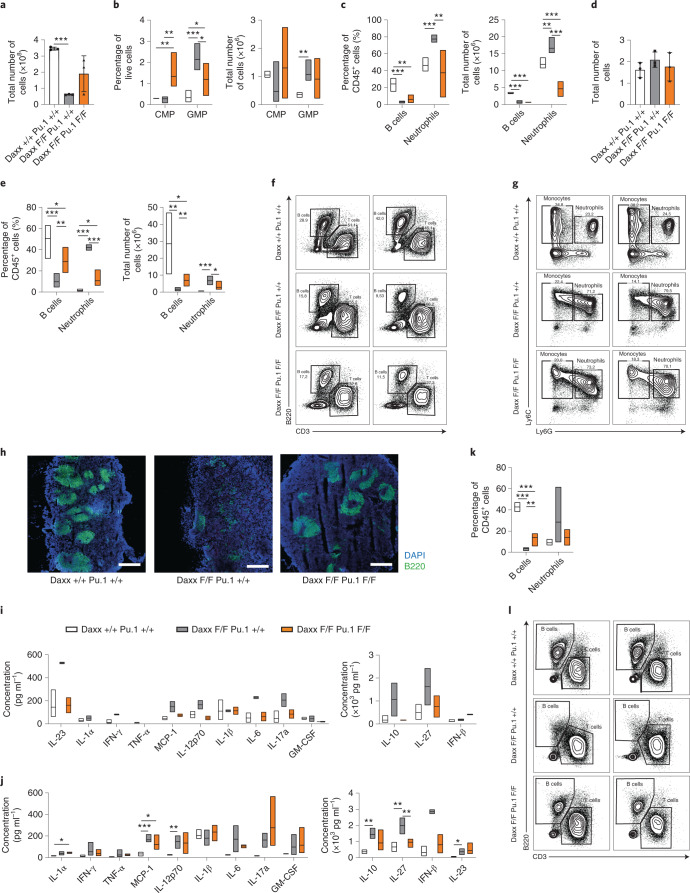
Concomitant loss of Daxx and Pu.1 partially restores lymphopoiesis while suppressing peripheral accumulation of neutrophils. **a**, Total BM cell counts (*n* = 3 mice per genotype) in four leg bones per animal. **b**, Percentage (left) and number (right) of CMPs and GMPs in the BM (*n* = 5 mice per genotype). **c**, Percentage (left) and number of B cells and neutrophils in the BM (*n* = 5 mice per genotype). **d**, Total spleen cell counts (*n* = 3 mice per genotype). **e**, Percentage (left) and number (right) of B cells and neutrophils in the spleen (*n* = 5 mice per genotype). **f**, Flow cytometry plots showing B and T cell-like populations in the spleen. **g**, Flow cytometry analysis showing neutrophil- and monocyte-like populations in the spleen. **f**,**g**, The percentages of cells in the gated regions of the plots are indicated. **h**, Representative immunofluorescence overview images of spleens stained with 4,6-diamidino-2-phenylindole (DAPI) and B220 (*n* = 2 independent experiments). Scale bars, 500 µm. **i**, Inflammatory cytokine levels in plasma at 3 w.p.i. (*n* = 5 WT and 2 Daxx-KO and DKO mice). **j**, Inflammatory cytokine levels in plasma at 8 w.p.i. (*n* = 5 mice per genotype). **k**, Percentage of B cells and neutrophils in the PB (*n* = 5 mice per genotype). **l**, Flow cytometry plots showing B and T cell-like populations in PB (*n* = 5 mice per genotype). **a**,**d**, Data are the mean ± s.d. **b**,**c**,**e**,**k**–**j**, Boxplots show the minimum and maximum values (box boundaries) and the mean (horizontal line). *P < 0.05, **P < 0.01, ***P < 0.001 and NS, not significant; Student’s *t*-test. Daxx F/F, Daxx KO and Daxx +/+, Daxx WT; Pu.1 F/F, Pu.1-KO and Pu.1 +/+, Pu.1 WT. Exact *P* values and numerical source data are provided. Source data

We next investigated whether the phenotypic alterations caused by Daxx loss were dependent on Pu.1 by using a *Spi1*^F/F^ line^42^. The BM cellularity was not significantly different in Daxx-KO mice compared with Daxx and Pu.1 double-KO (DKO) mice, although the latter displayed a tendency towards higher cellularity (Fig. 7a). At 8 w.p.i., the BM of the DKO mice displayed significant reduction in the GMP and neutrophil frequencies and numbers compared with the Daxx-KO (Fig. 7b,c), whereas the B220^+^ cells in the BM were still compromised (Fig. 7c). Conversely, in DKO spleens, the B-cell and neutrophil frequencies and numbers were normalized (Fig. 7d–g) along with spleen architecture and selected cytokines (at 3 and 8 w.p.i.; Fig. 7h–j). The B-cell frequency was also partially rescued in the DKO PB (Fig. 7k,l). Neutrophil and B-cell numbers and frequencies in the BM, spleen and PB were similar to WT following the loss of Pu.1 alone (Extended Data Fig. 8d,e).

**Fig. 8:**
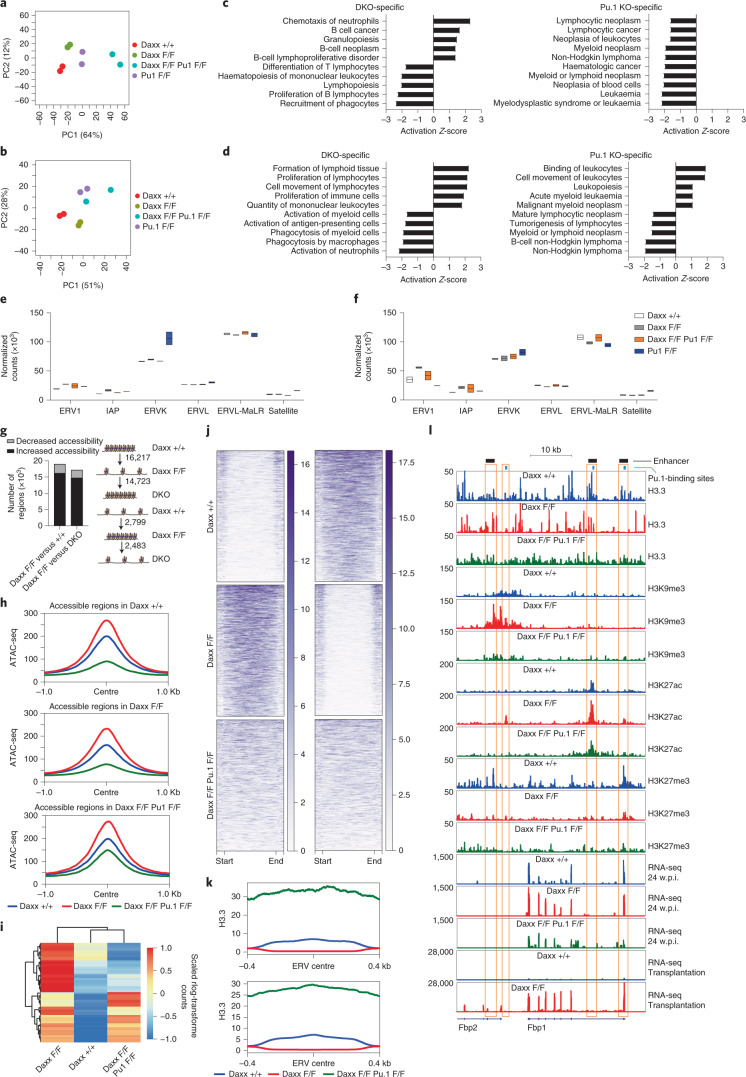
Partial rescue of the biological phenotype is associated with specific perturbations at the transcriptional and chromatin levels. **a**, PCA of the top-500 most variable genes in KLS cells collected at 3 w.p.i. **b**, PCA of the top-500 most variable genes in GMP cells collected at 3 w.p.i. **c**, Top-five activated or inhibited haematological functions and diseases associated with KLS cells with Daxx and Pu.1 DKO- (left) and Pu.1-KO-specific (right) gene expression changes. **d**, Top-five activated or inhibited haematological functions and diseases associated with GMP cells with DKO- (left) and Pu.1-KO-specific (right) gene expression changes. **c**,**d**, Data are the activation *Z*-score from IPA Fisher’s exact tests with multiple testing-adjusted *P* *<* 0.05. An activation *Z*-score > 2 suggests increased activation of the indicated biofunctions and an activation *Z*-score < −2 suggests increased inhibition. **e**, Normalized read counts of ERV/RTE subtypes and satellite repeats (*n* = 2 mice per genotype) in KLS cells. **f**, Normalized read counts of ERV/RTE subtypes and satellite repeats (*n* = 2 mice per genotype) in GMP cells. **e**,**f**, Boxplots show the minimum and maximum values (box boundaries) and the mean (horizontal line). **g**, Number of regions that were opened or closed in Daxx-KO versus WT (left) and the number of those regions that reverted back to WT condition in DKO KLS cells (right). **h**, ATAC-seq read distribution around the centre of IDR-reproducible peaks identified in WT (top), Daxx-KO (middle) and DKO (bottom) KLS cells. **i**, Heatmap of scaled normalized RNA-seq read counts for genes upregulated in Daxx single-KO KLS and increased accessibility in nearby enhancers. **j**, Heatmaps of H3K9me3 enrichment over distal regions segregated in regions gaining or losing H3K9me3. **k**, H3.3 enrichment plots at enhancers (top) and ERVs (bottom). **l**, Genome browser coverage plot of the *Fbp1* and *Fbp2* locus. Daxx F/F, Daxx KO and Daxx +/+, Daxx WT; Pu.1 F/F, Pu.1-KO and Pu.1 +/+, Pu.1 WT. Numerical source data are provided. Source data

**Extended Data Fig. 9:**
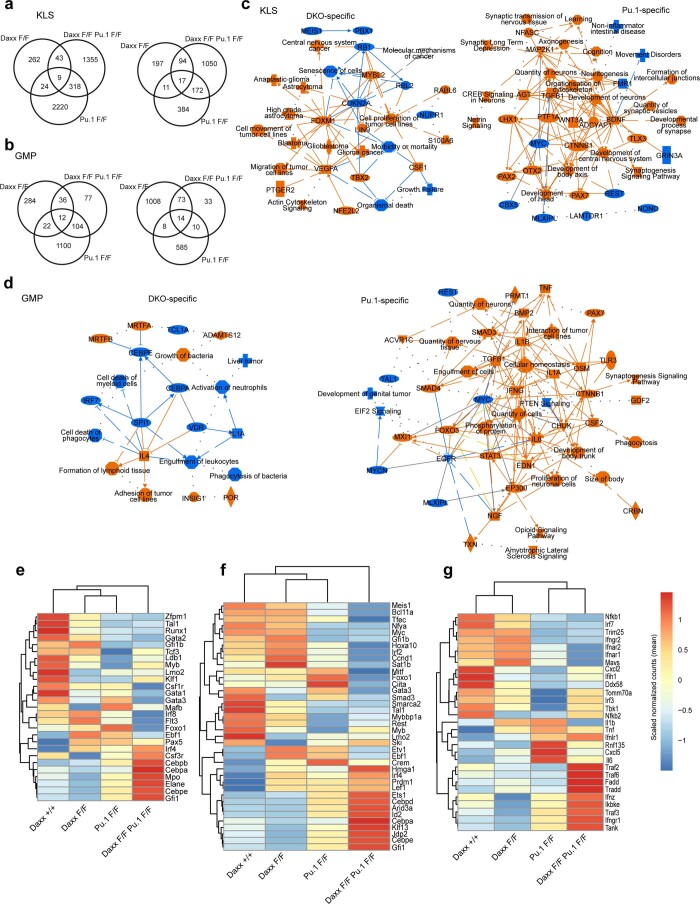
RNA-seq data from wild-type, *Daxx* single KO, *Daxx Pu.1* double KO and *Pu.1* single KO KLS and GMP cells collected at 3 w.p.i. **a**, Venn diagrams of differentially upregulated and downregulated genes in comparison to wild-type KLS. **b**, Venn diagrams of differentially upregulated and downregulated genes in comparison to wild-type GMP cells. **c**, IPA graphical summary for KLS Daxx Pu.1 double knock-out specific and Pu.1 single KO specific gene expression changes. **d**, IPA graphical summary for GMP Daxx Pu.1 double knock-out specific and Pu.1 single KO specific gene expression changes. **e**, Heatmap showing scaled expression of selected transcription factors and regulators involved in blood cell differentiation (*n* = 2 mice per genotype). **f**, Heatmap showing scaled expression of transcription factors regulated by Pu.1 (*n* = 2 mice per genotype). **g**, Heatmap showing scaled expression of interferon response genes and the dsRNA recognition machinery (*n* = 2 mice per genotype). Numerical source data provided in Source data. Source data

To link the phenotypic rescue to normalization of molecular perturbations, we studied changes in transcription, chromatin landscape and histone marks in single-KO and DKO mice (Supplementary Tables 9 and 10). The PCA analysis of RNA-seq data showed that each genotype clustered separately (Fig. 8a,b). Although DEGs from different comparisons (Daxx-KO versus WT, DKO versus WT and Pu.1-KO versus WT) partially overlapped, many genotype-specific changes also occurred, particularly in KLS cells (Extended Data Fig. 9a,b). Pathway analysis based on DKO-specific and Pu.1-KO-specific DEGs in KLS cells and GMPs identified distinct biological functions and diseases, including some associated with lymphoid neoplasms (Fig. 8c,d and Extended Data Fig. 9c,d). Clustering of expression for haematopoietic transcription factors and markers, Pu.1-target genes, ISGs and the dsRNA-sensing machinery revealed both common and genotype-specific changes between Pu.1-KO and DKO cells (Extended Data Fig. 9e–g). Altered expression of selected ERV/RTE subtypes in Daxx-KO KLS cells and GMPs were in part reverted in the DKO cells (Fig. 8e,f). Finally, Pu.1-KO KLS cells and GMPs showed upregulation of some ERV/RTE subtypes and satellite repeats, suggesting a role for Pu.1 in their regulation.

**Extended Data Fig. 10:**
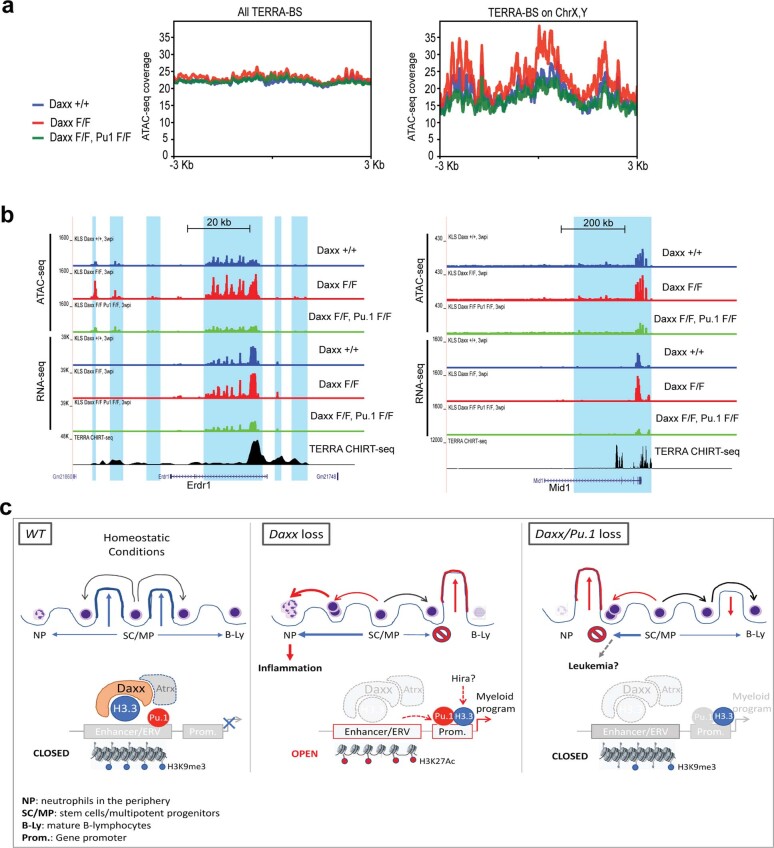
Changes in gene expression are associated with increased accessibility at TERRA-BS in sex chromosomes. **a**, ATAC-seq coverage around TERRA-BS across all chromosomes or across sex chromosomes at 3 w.p.i. **b**, Genome browser tracks for wild-type (blue), Daxx KO (red) and Daxx/Pu.1 KO (green) KLS cells at 3 w.p.i. around *Erdr1* and *Mid1*; TERRA CHIRT-seq based on published data^23^. Light-blue regions are called TERRA-BS. **c**, Proposed model: Daxx contributes to epigenetic barriers restricting fate/identity in haematopoietic stem cells and progenitors thus contributing to balanced differentiation output. Upon Daxx loss, stem cells enter differentiation and produce both myeloid and lymphoid-biased progenitors. However, while myeloid differentiation towards neutrophils is enhanced leading to inflammation, generation of mature B cells is strongly impaired. By inhibiting the pioneer TF Pu.1, the block of B-cell differentiation is partly relieved, while peripheral accumulation of neutrophils is hampered. Given that Pu.1/Daxx DKO progenitors display unique chromatin and transcriptome features and that both Daxx and Pu.1. inactivation/repression are linked to myeloid leukaemia, we hypothesize that loss of both genes may make progenitor cells susceptible to neoplastic transformation.

On investigation of the chromatin changes, we found that 90.8% of distal regions that opened following Daxx loss became closed in DKO cells (Fig. 8g). Conversely, the vast majority of distal regions found closed in the Daxx-KO KLS cells opened in DKO KLS cells. In addition, we found DKO-specific chromatin changes: 15,587 distal regions that were open in WT and Daxx-KO KLS cells became closed in DKO cells, while 3,922 distal sites that opened in DKO cells were closed in the other genotypes. When we compared open regions in WT, Daxx-KO and DKO KLS cells, we found that DKO cells generally displayed the lowest accessibility (Fig. 8h). Many of the genes that were significantly upregulated in the Daxx-KO KLS cells at 3 w.p.i. and resided near enhancers with increased accessibility were downregulated in the DKO cells (Fig. 8i). Finally, opening of TERRA-BS at sex chromosomes in Daxx-KO KLS cells was reverted in DKO cells (Extended Data Fig. 10a). Closing of chromatin at the TERRA-BS in the vicinity of the *Mid1* and *Erdr1* genes in the DKO cells correlated with rescued gene expression compared with the Daxx-KO cells (Extended Data Fig. 10b).

H3K9me3 alterations found in Daxx-KO HSPCs were reverted to some extent in DKO cells (Fig. 8j). In addition, we found that there were more distal regions in DKO HSPCs that gained H3K9me3 (7,862) than those with reduced H3K9me3 (1,147) compared with WT or Daxx-KO HSPCs. We also observed that the overall levels of H3.3 were markedly increased in DKO cells at enhancers and ERVs compared with both the WT and Daxx-KO cells (Fig. 8k).

Finally, we zoomed in on the locus encoding *Fbp1* and *Fbp2* (Fig. 8l), two critical regulators of gluconeogenesis that affect HSPC repopulation capacity via inhibition of glycolysis under the control of Setdb1 (ref. ^43^). *Fbp1* was upregulated in Daxx-KO KLS cells, whereas *Fbp2* remained silenced apart from transplantation settings. *Fbp1* silencing was restored in DKO cells, suggesting that Pu.1 is required for its induction. This is in accordance with three main regions bound by Pu.1 at this locus (as reported in^19^), two of which coincide with haematopoietic enhancers^18^. H3K27ac enrichment at these regions in Daxx-KO cells was reverted in DKO cells. Although H3.3 was reduced at the proximal enhancer in Daxx-KO cells, its levels were restored in DKO cells, suggesting involvement of another chaperone, such as Hira. A similar trend applied to H3K9me3 at this enhancer. The large block of H3K9me3 over the *Fbp2* gene and its regulatory regions, which was previously linked to Fbp2 upregulation in HSPCs^43^ following Setdb1 loss, was further enriched in Daxx-KO cells but not DKO cells. Finally, H3K27me3 was substantially reduced across the entire locus in the Daxx-KO and DKO cells, especially at the most proximal enhancer of *Fbp1*, suggesting that this mark may cooperate with Pu.1 loss for *Fbp1* silencing.

## Discussion

Together, our findings implicate the H3.3 chaperone and ERV/RTE repressor Daxx in the regulation of haematopoiesis and protection from inflammation. We propose that Daxx acts as an epigenetic barrier controlling cell plasticity in HSPCs in part via RTE control, in turn ensuring balanced cell differentiation (Extended Data Fig. 10c). Recent work in zebrafish implicates RTE-driven engagement of RNA-sensing in HSPC emergence in haematopoiesis^44^. In this respect, we showed that ERV/RTE induction correlates with the IFN type I-like response in Daxx-deficient LT-HSCs. IFN type I signalling is normally repressed in HSCs, as it may promote cell death^45^, but when activated in quiescent HSCs it causes cell-cycle entry^46^. Following acute Daxx loss, the number of LT-HSCs and other progenitor types did indeed increase. As it is unlikely that stem/progenitor cells are able to produce IFNs, ISG induction could be IFN-independent as reported in viral-infection models^47–50^. This could explain the moderate ISG induction in Daxx-KO LT-HSCs. Furthermore, it is possible that ERV/RTE derepression following Daxx loss may impair B-cell differentiation, as observed in mice lacking the ERV/RTE-silencing Setdb1–Kap1 complex, a Daxx interactor^51–55^.

Our study points to a link between Daxx and the pioneer transcription factor Pu.1. Pu.1 engagement does not seem to be an immediate effect of Daxx loss in HSCs. We instead observed progressive activation of a Pu.1 myeloid programme in haematopoietic progenitors with time and following stress, along with aggravation of neutrophilia. This mirrors the response to viral infections, where HSC activation is followed by a return to quiescence and an accumulation of myeloid-biased HSCs^56^, a phenomenon that has also been observed in ageing^57^. Thus, Pu.1 may contribute to the myeloid bias within a subset of Daxx-deficient HSCs that have returned to quiescence.

Concomitant Daxx and Pu.1 loss reverts some phenotypic perturbations found in Daxx-KO mice, including a partial recovery of B-cell differentiation and spleen architecture. This would be in agreement with the reported role of Pu.1 in inhibition of terminal B-cell and plasma-cell differentiation^58^. At the molecular level, the increase in enhancer accessibility that typifies Daxx-KO KLS cells is reverted by over 90% in DKO cells. Closing of chromatin in DKO cells at TERRA-BS correlated with rescued gene expression of neighbouring genes, suggesting that perturbations at TERRA-BS and its target genes, such as *Erdr1*, may contribute to the phenotypes caused by Daxx loss^26^. However, DKO progenitors also show unique chromatin and transcriptome alterations that are not found in single Pu.1- or Daxx-KO mice, suggesting a genetic interaction between the two pathways. In support of this hypothesis, Daxx loss causes reduction in the levels of both Pu.1 and H3.3 at enhancers, correlating with their increased enrichment at neighbouring genes. These findings suggest that Pu.1 and H3.3 may influence their reciprocal genome distribution.

Finally, dysfunction of intrinsic immunity mechanisms devoted to ERV/RTE silencing may predispose to inflammation and neoplastic transformation during ageing^59–62^. While transposition-driven mutagenesis could lead to the accumulation of potentially leukaemogenic mutations in HSCs and promote clonal haematopoiesis^63^, the resulting anti-viral response may suppress the expansion of mutated HSCs^64^. In this respect, neutrophilia and inflammation triggered by Daxx loss might be tumour-suppressive. Further genetic alterations (de novo or pre-existing) such as downregulation of Pu.1 may allow Daxx-deficient myeloid or lymphoid progenitors to escape differentiation and undergo neoplastic transformation, in accordance with the reported leukaemogenic effect of Pu.1 downregulation^65^. It is therefore possible that DKO mice may become leukaemic at old age.

Overall, this work implicates mechanisms governing accessibility to repeat elements in homeostasis of the haematopoietic system via cross-talk with pioneer transcription factors such as Pu.1. Perturbations of these mechanisms may contribute to inflammageing and predispose to leukaemogenesis.

## Methods

### Patient sample information

We included H&E staining of skin from patients with pyoderma gangrenosum in our study, which were performed by J.W. at the University of Bonn. The histological picture of the pyoderma gangrenosum lesions was taken from a skin sample of a patient with pyoderma gangrenosum during a normal diagnostic procedure. This routine procedure includes the preparation of H&E samples. Based on German law, it is possible to take pictures from these skin samples (the patient identity is unknown and no additional investigations were performed with these samples). As a result, a ‘patient consent’ is not needed. However, the patient did give informed consent to perform the skin biopsy during the diagnostic procedure. This is also in accordance with the Helsinki Ethical guidelines. J.W. has ethical *approval* from the University of Bonn, which in principle allows the use of skin material taken for diagnostic proposes in later research (BN090/04).

### Experimental model details

#### Mouse models and treatments

The C57BL/6N Daxx conditional-KO (*Daxx*^F/F^) mice were generated by Taconic Artemis. The targeting vector contained a neomycin (*NeoR*) gene surrounded by flipase sequences (*FRT*) and a puromycin (*PuroR*) gene surrounded by F3 sites, which were removed by in vivo Flp-mediated recombination. *Daxx* exons 2–7 were flanked by LoxP sites. C57BL/6N Daxx mice were crossed with C57BL/6J *Csf1rCre* (JAX, 029206), C57BL/6J *Mx1Cre* (JAX, 003556) or C57BL/6J *Rosa26CreERT2* (JAX, 008463) mice. The C57BL/6N Hira conditional-KO (Hira^F/F^) mice were provided by P. Adams^66^ and crossed with C57BL/6J *Mx1Cre* mice. The C57BL/6J Pu.1 conditional-KO (*Pu.1*^F/F^) mice were provided by E. Mass (JAX, 006922) and crossed with C57BL/6 *Daxx*^F/F^;*Mx1Cre* mice. C57BL/6J CD45.1 mice were used (JAX, 002014) for the BM chimaera experiments. Genotyping of the C57BL/6N Daxx mice was performed using an Extract-N-amp tissue PCR kit (Sigma-Aldrich) with the primers DAXX_33 (5′-AGATCCTGTCTCTCCTGTCTATCC-3′) and DAXX_34 (5′-CACTGGGTAGACTAGACTGTGGC-3′). To check for recombination induced by Cre recombinase, we used the primers DAXX_29 (5′-GGAGGGAGTCGAAGAGTTGG-3′), DAXX_30 (5′-TGCGTTTCCTGTCTTTCGG-3′) and DAXX_recombined (5′-GCTCACGCCTTTAGTCCGAA-3′). C57BL/6N *Hira* mice were genotyped using the primers 2292_27 (5′-AATGGTGCTTGCTTTTGTGG-3′) and 2292_28 (5′-CCTGCTACCTTATTCTCCAGTCC-3′), and the primer 2293_30 (5′-GCATTACTTAATCCCCAGATGC-3′) was added for the analysis of recombination efficiency. Genotyping of the C57BL/6 *Pu.1* mice was performed with the primers PU1_Flox_FW (5′-CTTCACTGCCCATTCATTGGCTCATCA-3′) and PU1_Flox_Rev (5′-GCTGGGGACAAGGTTTGATAAGGGAA-3′), and the primer PU1_MUT (5′-CAACCGGATCTAGACTCGAGGA-3′) was added to determine the recombination efficiency. Cre mice were genotyped according to protocols released by The Jackson Laboratory. The mice were housed under specific pathogen-free conditions at the central animal facility of the University College London and German Center for Neurodegenerative Diseases. The mice were bred and subjected to listed procedures according to protocols approved by the English (Home Office) and German authorities (Landesamt für Natur, Umwelt und Verbraucherschutz Nordrhein-Westfalen). Activation of Cre in *Daxx*;*RosaCre*^ERT2^ mice was induced by administration of 80 mg kg^−1^ tamoxifen in corn oil (Sigma-Aldrich) via oral gavage on five consecutive days (ATAC-seq was performed at 3 w.p.i.) or by administration of 100 mg kg^−1^ tamoxifen in corn oil via intraperitoneal injection (five consecutive days, break of two days, two consecutive days; genomic and phenotypic analyses were performed at 3 d.p.i. and 2 w.p.i.). For the induction of Cre activation in Daxx-KO, Hira-KO and Daxx and Pu.1 DKO, Mx1Cre mice received three intraperitoneal injections of 300 µg pI:pC (Sigma-Aldrich) administered every other day. The mice were usually treated at 5–11 weeks of age. Mice of both sexes were used in this study. The *n* value in the figure legends reflects the number of mice analysed in each experiment.

#### BM chimaera mice

For BM transplantation studies, C57BL/6J CD45.1 mice received a lethal irradiation dose of 10 Gray before transplantation of 2.5 × 10^6^ CD45.2 *Daxx*^+/+^;*Mx1Cre* or *Daxx*^F/F^;*Mx1Cre* BM cells together with 1 × 10^5^ CD45.1 BM support cells. Peripheral blood samples were isolated from the tail vein 4–5 (first time point) and 8–12 weeks (second time point) post transplantation. The mice were euthanized by CO_2_ exposure 12–17 weeks post transplantation and analysed as described earlier. Populations of KLS and GMP cells were sorted from recipient BM as described in the ‘RNA-seq’ section.

### Method details

#### Haematopoietic-cell and organ preparation

Peripheral blood samples were collected from the tail vein or by heart puncture. For the isolation of haematopoietic organs, the mice were euthanized by CO_2_ exposure. Unless otherwise indicated, the mice were killed two months post treatment. Bone marrow was isolated from the femur and tibia of the hind legs. All isolated organs were directly processed for analysis, frozen for cryopreservation or fixed in neutral buffered formalin (Sigma-Aldrich) for subsequent paraffin embedding. HPCs were isolated from BM using an EasySep mouse hematopoietic progenitor cell isolation kit (STEMCELL Technologies) according to the manufacturer’s instructions. If required, RBCs were lysed for 10 min in ammonium chloride (STEMCELL Technologies) and washed twice before downstream analysis.

#### Analysis of BM cellularity

Mice were killed two months after induction, except when indicated otherwise. BM cells were isolated from all four hind-leg bones and collected in equal volumes of PBS. The number of cells was determined using a TC20 automated cell counter (Bio-Rad). The RBCs were subsequently lysed for 10 min in ammonium chloride (STEMCELL Technologies), washed twice and collected in equal volumes of PBS. The number of cells in the samples was calculated again following after RBC lysis. If indicated, analyses were performed separately for male and female mice to control for differences in body size and weight.

#### Flow cytometry and cell sorting

For flow cytometry analysis, cells were isolated from the specified organs. Dead cells were stained by propidium iodide (Sigma-Aldrich) or Aqua Zombie fixable viability dye (BioLegend) according to the manufacturer’s protocol. Fluorochrome-labelled antibodies, as indicated in Supplementary Table 6, were added to the cell suspension for 30 min on ice. The CD16 and CD32 receptors were blocked with CD16 and CD32 antibodies unless the cells were specifically stained for CD16/32. Intracellular staining was performed according to the manual of the Intracellular fixation and permeabilization buffer set (eBioscience). Lin^−^ antibody panels contained antibodies to B220, Gr-1, CD11b, TER-119 and CD3e. For ATAC-seq, the Lin^−^ antibody panel included antibodies to B220, CD19, Gr-1, CD11b, CD11c, NK1.1, TER-119 and CD3e. For RNA-seq, the Lin^−^ antibody panel included antibodies to B220, Gr-1, CD11b, TER-119, CD8, CD4 and CD3e. For staining with multiple brilliant violet dyes, the BD Horizon brilliant stain buffer was used according to the manufacturer’s instructions. For the assessment of apoptotic B cells, cells isolated from the spleen were first stained with antibodies to CD45, CD11b and B220, followed by staining with an CellEvent caspase-3/7 green flow cytometry assay kit (Invitrogen) according to the manufacturer’s instructions. The cells were washed and filtered through a 70 µm filter before flow cytometric analysis using a BD LSRFortessa, BD FACSymphony, BD FACSCelesta, BD FACSAriaIII or Beckman Coulter Gallios machine. Cell sorting was performed on a BD FACSAriaIII or BD FACSAria Fusion machine using a 70 µm or 100 µm nozzle. Data were analysed using the FlowJo software. Neutrophils were gated as CD11b^+^Ly6G^+^Ly6C^intm^Gr-1^hi^ and eosinophils as CD11b^+^Ly6C^intm^SSC^hi^Ly6G^–^Gr-1^+^.

#### CFU assay with re-plating

For the CFU assays, HPCs were isolated from BM as described in the ‘Haematopoietic-cell and organ preparation’ section. The HPCs were plated (15,000 cells per 35 mm dish) in MethoCult M3231 medium supplemented with 100 ng ml^−1^ SCF, 10 ng ml^−1^ GM-CSF, 10 ng ml^−1^ IL-3 and 10 ng ml^−1^ IL-6 (all STEMCELL Technologies) to promote myeloid-cell formation. The cells were cultivated for 1 week at 37 °C. Next, the colonies were counted under a microscope and 15,000 cells were re-plated under the same conditions. After another week of cultivation at 37 °C, the colonies were again counted under a microscope.

For the CFU assays with LT-HSCs, Lin^−^ cells were enriched using a MojoSort mouse hematopoietic progenitor cell isolation kit (BioLegend). LT-HSCs were then sorted using a BD FACSAriaIII cell sorter with antibodies to Lin (B220, CD19, Gr-1, CD11b, CD11c, NK1.1, TER-119 and CD3e), Sca-1, c-Kit, CD150 and CD48. The LT-HSCs were collected using the following gating strategy: (1) c-Kit^+^Lin^−^, (2) c-Kit^+^Sca-1^+^ and (3) CD48^−^CD150^+^. To generate myeloid colonies, 400 LT-HSCs were sorted into 270 µl IMDM medium (STEMCELL Technologies) supplemented with 2% FBS, 1% Pen–Strep and 1% l-glutamine, with the addition of the following growth factors: SCF (100 ng ml^−1^), GM-CSF (10 ng ml^−1^), IL-3 (10 ng ml^−1^) and IL-6 (10 ng ml^−1^). All growth factors and cytokines were purchased from Preprotech. After the addition of 30 µl Pen–Strep, the cell suspension was added to 3 ml MethoCult M3231 (STEMCELL Technologies) and 2 × 1.1 ml was seeded into 35 mm dishes. The colonies were counted on day 7. On day 10, the cells were harvested and 3 × 10^5^ cells were re-plated. Residual cells were analysed by flow cytometry. Seven days after re-plating, the new colonies were counted and on day 10, the cells were harvested and analysed by flow cytometry.

#### RNA isolation and quantitative PCR with reverse transcription

Total RNA was isolated from BM cells using an RNeasy mini kit (Qiagen) according to the manufacturer’s instructions. The RNA concentration was determined using a ND1000 Spectrophotometer (NanoDrop), followed by reverse transcription using a High-capacity cDNA reverse transcription kit (Applied Biosystems). Quantitative real-time PCR was performed on a 7500 Fast real-time PCR system (Applied Biosystems) using the Fast SYBR Green master mix (Applied Biosystems) with the primers mDaxx sense (5′-GATGACTATAGGCCAGGCGT-3′), mDaxx antisense (5′-TCGTCTCTTCTGTCTCTCGC-3′), mHira sense (5′-GTTGTCATTTGGAATGCCGTGA-3′), mHira antisense (5′-CAGCGTCCTCCATACCTTCA-3′), mTBP sense (5′-AGCTCTGGAATTGTACCGCAG-3′) and mTBP antisense (5′-GACTGCAGCAAATCGCTTGGG-3′). The relative abundance of the specific transcripts was normalized to TATA box binding protein (TBP) messenger RNA and calculated using the $2^{{{-\Delta\Delta}{c}}_{\rm{t}}}$ method.

#### Western blotting

Cells were washed in PBS and lysed in RIPA buffer with cOmplete protease inhibitor cocktail (Roche). The lysed samples were sonicated for 10 s and the protein concentrations were determined using a Pierce protein assay kit (Thermo Fisher) according to the manufacturer’s instructions. The proteins were separated by SDS–PAGE and blotted on a nitrocellulose membrane. Blocking was performed for 1 h in 5% non-fat dry milk (PBS-T) or 4% BSA (TBS-T) at room temperature and the membranes were incubated overnight with primary antibodies at 4 °C. The primary antibodies were detected by horseradish peroxidase-conjugated secondary antibodies and subsequently visualized using the ChemiDoc XRS+ imaging system (Bio-Rad). The antibodies used are specified in Supplementary Table 12. Normalization of protein levels was performed by densitometry (ImageLab software, Bio-Rad).

#### Immunofluorescence and H&E staining

For immunofluorescence imaging, cells were spun down using a Cytospin 4 (Thermo Fisher Scientific) cytocentrifuge at 600 r.p.m. for 5 min. Frozen sections were cut to 7–15 µm sections using a CryoStar NX70 (Thermo Fisher Scientific) cryostat. The cells and tissue sections were fixed in 4% paraformaldehyde for 20 min, washed three times in PBS-T (PBS with 0.1% Triton X-100) and permeabilized for 15 min using 0.3% Triton X-100. Blocking was performed for 1 h in 5% goat serum and the samples were incubated with primary antibodies overnight at 4 °C. The antibodies are listed in Supplementary Table 12. After washing in PBS-T, the samples were incubated with secondary antibodies for 90 min at room temperature, washed 3× in PBS-T and the nuclei were stained for 10 min in 1 µg ml^−1^ Hoechst 33342 (Thermo Fisher Scientific), followed by additional wash steps. The cells and tissue sections were mounted using Roti-Mount Aqua (ROTH) medium. Images were obtained on a Zeiss LSM700 or LSM800 laser scanning microscope. For the H&E staining, organs were isolated from mice, fixed in 10% neutral buffered formalin (Sigma-Aldrich) and subsequently processed for paraffin embedding. Tissue sections were cut to 5–15 µm using a CUT5062 microtome (SLEE medical). The sections were washed twice for 5 min in xylene, followed by a 5 min incubation in a 90:10 xylene:ethanol mix. Subsequently, rehydration steps were performed in absolute ethanol (2×), 95% ethanol, 80% ethanol and 70% ethanol, 5 min each, followed by a brief rinse in water. The samples were incubated with haematoxilin (Life Technologies) for 2 min, followed by rinsing in water and 0.25% ammonia water. The slides were incubated for 1 min in 95% ethanol, followed by a 2 min stain in alcoholic eosin Y (Sigma-Aldrich). Dehydration was performed in 95% ethanol and absolute ethanol (3×; 1 min each), followed by incubation in xylene (3×; 1 min each). The sections were mounted with DPX (Sigma-Aldrich). Images were taken on a Zeiss Epi-Scope microscope.

#### Cytokine analysis

Blood samples were obtained from mice and spun at 1,000*g* for 10 min at 4 °C to collect the plasma. Cytokine concentrations were determined using a LEGENDplex mouse inflammation panel (BioLegend, 740446) and mouse HSC panel (BioLegend, 740677) according to the manufacturer’s recommendations. Analysis was performed on a BD FACSymphony machine and using the LEGENDplex data analysis software (BioLegend).

#### Northern blotting

RNA was isolated from BM cells using a Direct-zol RNA miniprep plus kit (Zymo Research) according to the manufacturer’s protocol. Subsequently, DNA was digested using a Turbo DNA-free kit (Thermo Fisher) following the manufacturer’s protocol. The RNA was recovered and concentrated using an RNA clean and concentrator-5 kit (Zymo Research). Northern blot analysis was performed according to standard procedures. Briefly, 5 µg of total RNA was run on a 1% denaturing agarose gel. The RNA was transferred to a Hybond-N+ membrane (GE Healthcare) by capillary transfer and fixed by ultraviolet-light crosslinking. The membrane was pre-hybridized at 42 °C for 20 min and then hybridized in PerfectHyb plus hybridization buffer (Sigma) containing 1 × 10^6^ cpm ml^−1^ of ^32^P-labelled TERRA DNA probe 5′-(TAACCC)5-3′ and 0.1 mg ml^−1^ herring sperm DNA (Thermo Fisher). Hybridization was carried out overnight at 42 °C. The following day, the membrane was washed once in low stringency buffer (2×SSC and 0.1% SDS) at room temperature for 5 min and twice in high stringency buffer (0.5×SSC and 0.1% SDS) at 42 °C for 20 min. The membranes were exposed to muliautoradiography film at −80 °C. After exposure, the membrane was stripped in boiling stripping buffer (0.1% SDS and 5 mM EDTA) and re-hybridized using a GAPDH probe (5′-GTAGACCCACGACATACTCAGCACCGGCCTCACCCCATT-3′) as a loading control.

#### RNA-seq of cells isolated from transplanted animals

CD45.2^+^ KLS and GMP (Lin^−^c-Kit^+^Sca-1^−^CD16/32^+^CD34^+^) populations were sorted from the BM of transplanted mice. Total RNA was isolated from samples containing between 4,700 and 246,000 cells using TRIzol reagent (Invitrogen) and a miRNeasy micro kit (Qiagen) according to the manufacturer’s protocol. The precipitated RNA was resuspended in RNase-free water and the RNA quantity and quality (RINe) were assessed via the HS RNA analysis screen tape assay on a 4200 TapeStation system (Agilent Technologies). The total RNA was converted into double-stranded complementary DNA libraries as the template for high-throughput sequencing using the Ovation SoLo RNA-seq kit (NuGEN Technologies). Briefly, the DNA was digested and first-strand DNA was synthesized using random hexamers. After second-strand synthesis, the ends were repaired, followed by adaptor ligation and PCR pre-amplification. NuGEN proprietary AnyDeplete probes were added to selectively block fragments originating from ribosomal RNA from amplification in the subsequent PCR reaction. Size-selection and purification of cDNA fragments of approximately 200–500 bp in length was performed using AMPure XP beads (Beckman Coulter). The size distribution of the cDNA libraries was measured using the HS D1000 DNA assay on a 4200 TapeStation system (Agilent Technologies). The cDNA libraries were quantified using a KAPA library quantification kit (Kapa Biosystems). After cluster generation on a cBot (Illumina), the libraries were sequenced in a paired-end 2 × 101 bp run on a HiSeq 1500 system (Illumina) using TruSeq v3 chemistry and de-multiplexed using CASAVA version 1.8.4. The experiment was run in duplicate.

#### RNA-seq of cells collected at steady state

LT-HSCs (Lin^−^c-Kit^+^Sca-1^+^Il-7Ra^−^Flk2^−^CD48^−^CD150^+^), GMPs, and MPP3 (Lin^−^c-Kit^+^Sca-1^+^Il-7Ra^−^Flk2^−^CD48^+^CD150^−^), MPP4 (Lin^−^c-Kit^+^Sca-1^+^Il-7Ra^−^Flk2^+^CD48^+^CD150^−^) and KLS cells were sorted from BM 3 d.p.i. (*RosaCreER* mice: LT-HSCs), 3 w.p.i. (*Mx1Cre* mice: GMPs and KLS, MPP3 and MPP4 cells) or 24 w.p.i. (*Mx1Cre* mice: KLS cells and GMPs). Total RNA was isolated from 2,000–5,000 cells using a NucleoSpin RNA XS plus kit (Macherey-Nagel) according to the manufacturer’s instructions. The RNA quantity and quality (RINe) were assessed using the HS RNA analysis screen tape assay on a 4200 TapeStation system (Agilent Technologies). Total RNA was converted into double-stranded cDNA libraries as a template for high-throughput sequencing using the SMARTer stranded total RNA-seq kit v.2–pico input mammalian kit (TaKaRa Bio) according to the manufacturer’s instructions. Briefly, after first-strand cDNA synthesis with SMARTer oligonucleotides, barcoded adaptors for Illumina sequencing were added via limited-cycle PCR. The PCR products were purified and ribosomal cDNA was then depleted. The remaining cDNA fragments were further amplified by PCR (15 cycles) with primers universal to all libraries. After purifying the resulting PCR products, the size distribution of the cDNA libraries was measured using the HS 5000 DNA assay on a 4200 TapeStation system (Agilent Technologies) and the cDNA concentrations were determined using a Qubit dsDNA HS assay kit (Thermo Fisher Scientific). After cluster generation, the samples were sequenced either as single-end 1 × 100 bp or paired-end 2 × 100 bp on a NovaSeq 6000 system (Illumina). The experiments were run in duplicate or triplicate.

#### ATAC-seq of *RosaCreER* mice at 3 w.p.i

For ATAC-seq, *RosaCreER* Daxx mice were killed 3 weeks after tamoxifen treatment. The BM was isolated from the femur and tibia, followed by RBC lysis and sorting of CMP (Lin^–^CD45.2^+^c-Kit^+^Sca-1^–^CD16/32^–^CD34^+^) and GMP populations following antibody staining. Enrichment of c-Kit^+^ cells using CD117 MicroBeads (Miltenyi Biotec) was performed before sorting LT-HSCs (Lin^–^CD45.2^+^c-Kit^+^Sca-1^+^CD48^–^CD150^+^). Between 8,000 and 50,000 flow-sorted cells were collected in flow buffer (PBS, 2% FBS and 2 mM EDTA) and immediately processed following previously published protocols^67^. Briefly, the sorted cells were spun down at 500*g* for 5 min at 4 °C, washed once in cold PBS and spun down in 50 μl cold lysis buffer (10 mM Tris–HCl pH 7.4, 10 mM NaCl, 3 mM MgCl_2_ and 0.1% IGEPAL CA-630) at 500*g* for 10 min at 4 °C. Immediately thereafter the tagmentation reaction was started by adding Nextera’s Tn5 transposase (TDE1) in reaction buffer. The transposition reaction mix was incubated for 30 min at 37 °C, followed by DNA purification using a MinElute PCR purification kit (Qiagen). ATAC-seq libraries were generated from tagmented DNA by PCR amplification using Nextera index primers (Illumina) according to the manufacturer’s protocol. The ATAC-seq libraries were purified using a PCR purification MinElute kit (Qiagen) and quantified using KAPA library quantification kits (Kapa Biosystems) and a D1000 assay on a Tapestation 4200 (Agilent). The libraries were sequenced in a paired-end 2 × 101 bp run on a HiSeq 1500 system (Illumina) using TruSeq v3 chemistry and de-multiplexed using CASAVA version 1.8.4. In addition, ATAC-seq libraries from CMP and GMP cells were sequenced as a single-read 75 bp, rapid run on the HiSeq 1500 system using HiSeq Rapid v.2 chemistry. The resulting data were also de-multiplexed using CASAVA version 1.8.4. The experiment was run in duplicates.

#### ATAC-seq of 3 d.p.i. LT-HSCs and 3 w.p.i. KLS cells

LT-HSCs (Lin^–^c-Kit^+^Sca-1^+^Il-7Ra^–^Flk2^–^CD48^–^CD150^+^) and KLS cells were sorted from BM 3 d.p.i. (LT-HSCs) or 3 w.p.i. (KLS cells). The LT-HSCs (2,000) and KLS cells (5,000) were directly sorted into 12.5 µl of 2×TD buffer (Illumina) using the FACSAriaIII. Directly after sorting, 0.25 µl of 1% digitonin (Promega), 0.25 µl of 10% Tween-20 (Merck Millipore) and nuclease-free water were added to each sample to obtain a final volume of 23.5 µl. After mixing the samples by vortexing, 1.5 µl of the TDE tagmentation enzyme (Illumina) was added to each sample. The samples were incubated for 30 min at 37 °C with shaking at 1,000 r.p.m. to induce tagmentation, followed by DNA purification using a MinElute reaction clean up kit (Qiagen). ATAC-seq libraries were generated from the tagmented DNA by PCR amplification (13 cycles) using the ‘IDT for Illumina Nextera DNA UD Indexes Set A’ primers (Illumina) and the KAPA HiFi hot start ready mix (Roche). The ATAC-seq libraries were size selected (175–1,300 bp), purified using SPRIselect beads (Beckman Coulter) and quantified using a HS D5000 assay on a Tapestation 4200 (Agilent). The libraries were sequenced in a paired-end 2 × 100 bp run on a NovaSeq 6000 system (Illumina). The experiments were run in duplicate or triplicate.

#### CUT&Tag

HSPCs were isolated by magnetic cell sorting by first enriching Lin^−^ cells using an EasySep mouse hematopoietic progenitor cell isolation kit (STEMCELL Technologies) according to the manufacturer’s instructions and then isolating c-Kit^+^ cells from the resulting cell suspension using mouse CD117 MicroBeads (Miltenyi Biotech). The nuclei were isolated, washed and frozen in wash buffer according to the ‘Bench top CUT&Tag V.3’ protocol (https://ww.protocols.io/view/bench-top-cut-amp-tag-bcuhiwt6)^68^. For the actual CUT&Tag experiment, we followed the CUT&Tag-direct protocol described by Henikoff et al.^69,70^ using the CUTANA pAG-Tn5 enzyme (Epycypher). Briefly, aliquots of 30,000–45,000 native nuclei per reaction were bound to activated Concanavalin A beads. After successive incubations with primary (overnight at 4 °C) and secondary (0.5–1 h) antibodies in wash buffer (20 mM HEPES pH 7.5, 150 mM NaCl, 0.5 mM spermidine and 1×protease inhibitor), the beads were washed and resuspended in pAG-Tn5 (1:20 dilution) in 300-wash buffer (wash buffer containing 300 mM NaCl) for 1 h. The incubations were performed at room temperature, except when otherwise stated, in volumes of 25–50 µl in low-retention PCR tubes. Tagmentation was performed for 1 h in 300-wash buffer supplemented with 10 mM MgCl_2_. Following tagmentation, the beads were washed in 50 µl TAPS buffer (10 mM TAPS pH 8.5 and 0.2 mM EDTA), resuspended in 5 µl SDS release buffer (0.1% SDS and 10 mM TAPS pH 8.5) and incubated for 1 h at 58 °C. The SDS was neutralized with 15 µl of 0.67% Triton X-100, and 4 µl of dual-indexed primers from the ‘IDT for Illumina Nextera DNA UD Indexes Set A’ (Illumina) as well as 25 µl of NEBNext high-fidelity 2×PCR master mix (NEB) were added. Gap filling and 18 cycles of PCR were performed, followed by clean-up with 65 µl of SPRIselect beads (Beckman Coulter). We used antibodies, at a 1:20 dilution, to H3K9me3 (Active Motif), H3K27ac (Epicypher), H3K27me3 (Cell Signaling Technologies), histone H3.3 (Merck Millipore) and Pu.1 (Abcam). The size distribution and concentration of the CUT&Tag libraries were measured using the HS 5000 DNA assay on a 4200 TapeStation system (Agilent Technologies). After cluster generation, the samples were sequenced either as paired-end 2 × 75 bp on a NextSeq 500 system (Illumina) or as paired-end 2 × 50 bp on a NovaSeq 6000 system (Illumina). The experiments were run in duplicate or triplicate.

#### Bioinformatics analysis

A detailed description of the bioinformatics analyses for the ATAC-seq, RNA-seq and CUT&Tag data can be found in Supplementary Note 1.

#### Statistics and reproducibility

Statistical analyses were performed using GraphPad Prism (GraphPad Software) or the R program. Summarized views on data that underlie the statistical tests as well as the exact *P* values are available in the source data. The statistical details for each experiment are also provided in the figure legends. Statistical tests were run as two-sided tests, when appropriate. Most experiments were performed independently at least twice; details are provided in the figure legends. The genomic assay data (Figs. 1,5,6,8 and Extended Data Figs. 1,7–10) are based on two or three biological replicates. The experimental data in Figs. 2g,i,k,p, 3b,f, 4e,f, 7a,d,i and Extended Data Figs. 2d, 4b,c, 8b,d,e are based on biological replicates (*n* is given in the figure legends) that were not repeated independently on a different day.

### Reporting Summary

Further information on research design is available in the Nature Research Reporting Summary linked to this article.

## Online content

Any methods, additional references, Nature Research reporting summaries, source data, extended data, supplementary information, acknowledgements, peer review information; details of author contributions and competing interests; and statements of data and code availability are available at 10.1038/s41556-021-00774-y.

## Supplementary information

Supplementary Information Supplementary Note 1 and Supplementary Codes 1,2.

Reporting Summary

Peer Review Information

Supplementary Table 1 Supplementary Tables 1–12.

## Data Availability

All sequencing data that support the findings of this study can be found at Gene Expression Omnibus under the accession number GSE119309. An overview of the genomics studies run as part of this study can be found in Supplementary Table 11. Previously published sequencing data that were re-analysed here are available under the accession codes GSE60101 and GSE79180 (SRR2062971 and SRR2062968). The GTF and FASTA files used for Bioinformatics analysis (mm10, GENCODE release M14) can be downloaded from GENCODE (https://www.gencodegenes.org/mouse/release_M14.html). All other data supporting the findings of this study are available from the corresponding author on reasonable request. Source data are provided with this paper.

## Source data

Source Data Fig. 1 Statistical source data.
Source Data Fig. 2 Statistical source data.
Source Data Fig. 3 Statistical source data.
Source Data Fig. 4 Statistical source data.
Source Data Fig. 5 Statistical source data.
Source Data Fig. 6 Statistical source data.
Source Data Fig. 7 Statistical source data.
Source Data Fig. 8 Statistical source data.
Source Data Extended Data Fig. 1 Statistical source data.
Source Data Extended Data Fig. 2 Statistical source data.
Source Data Extended Data Fig. 2 Unprocessed western blots and gels.
Source Data Extended Data Fig. 3 Statistical source data.
Source Data Extended Data Fig. 3 Unprocessed western blots and gels.
Source Data Extended Data Fig. 4 Statistical source data.
Source Data Extended Data Fig. 4 Unprocessed Western Blots and gels.
Source Data Extended Data Fig. 5 Statistical source data.
Source Data Extended Data Fig. 5 Unprocessed western blots and gels.
Source Data Extended Data Fig. 6 Statistical source data.
Source Data Extended Data Fig. 7 Statistical source data.
Source Data Extended Data Fig. 8 Statistical source data.
Source Data Extended Data Fig. 9 Statistical source data.

## Code availability

Rscripts exemplifying the counting of paired-end (Supplementary Code 1) and single-end RNA-seq reads (Supplementary Code 1) are available online in Supplementary Note 1. The custom Rscript to generate non-overlapping mouse gene annotations as well as the created gene annotations used for RNA-seq read counting are available from the corresponding author on request.
