# Supplementary material for: Aberrant chromatin landscape following loss of the H3.3 chaperone Daxx in haematopoietic precursors leads to Pu.1-mediated neutrophilia and inflammation
Source: Nat Cell Biol. 2021 Dec 7;23(12):1224–39. doi: 10.1038/s41556-021-00774-y (PMC8683376; doi:10.1038/s41556-021-00774-y)
Supplement: Supplementary file 1 — Supplementary Note 1 and Supplementary Codes 1,2. [file 41556_2021_774_MOESM1_ESM.pdf]

nature portfolio

https://doi.org/10.1038/s41556-021-00774-y

Supplementary information

Aberrant chromatin landscape following loss of the H3.3 chaperone Daxx in haematopoietic precursors leads to Pu.1-mediated neutrophilia and inflammation

In the format provided by the authors and unedited

Supplementary note 1: Bioinformatics analysis

ATAC-seq data analysis

Pre-processing, mapping and filtering of ATAC-seq reads

Sequencing read quality was assessed with FastQC (Version 0.11.5; http://www.bioinformatics.babraham.ac.uk/projects/fastqc). Nextera adapter sequences were removed from reads using Trimmomatic (Version 0.36 with options -phred33, seedMismatches=2, palindromeClipThreshold=30, simpleClipThreshold=10 and option MINLEN:36. Only reads with a minimum length of 36 bp were kept after trimming and were aligned against the mouse genome (mm10, GENCODE release M14) using STAR (Version 2.5.3 with option --alignIntronMax 1 and --alignMatesGapMax 1800). Reads that (1) mapped to more than one locus; (2) mapped to the mitochondrial genome; and (3) were read duplicates were excluded. Filtering was done with samtools (Version 1.4.1) and MarkDuplicates (option REMOVE_DUPLICATES=true) from Picard tools (Version 2.9.2) was run for the deduplication. Next, the resulting bam files were converted to standard bed files using bedtools (Version 2.25.0), where bam files from paired-end libraries were treated as single-end to combine the bed files from the two sequencing runs. Finally, the start position of reads was offset by +4 bp if they mapped to the positive strand and the end position of reads was offset by -5 bp if they mapped to the negative strand. Thereby, the 5'-end of the aligned reads matches the Tn5 transposase cut site.

Peak calling

The shifted bed files were used as input for the ENCODE ATAC-seq analysis pipeline (Vs 0.3.4; https://www.encodeproject.org/atac-seq/; https://github.com/kundajelab/atac_dnase_pipelines). The pipeline uses MACS2 for peak calling and uses Irreproducible Discovery Rate (IDR) to compare consistency of ranks of peaks in individual replicate peak sets to identify a set of high confidence, reproducible set of peaks. The resulting peak files were sorted by coordinate and overlapping peaks were merged with bedtools merge. Peaks were annotated with annotatePeaks.pl script from the HOMER software package (Version 4.9.1) to identify the nearest transcription start site using an mm10 genome annotation file. Peaks within +/-1000 bp from the transcriptional start site (TSS) were defined as proximal peaks, whereas all other peaks were defined as distal peaks.

Ingenuity canonical pathway analysis and transcription factor motif analysis

First, distal ATAC-seq peaks were differentiated into peak sets that were identified as peak only in *Daxx*$^{+/+}$*;RosaCre*^{ER} cells (closed peak set), only in *Daxx*$^{F/F}$*;RosaCre*^{ER} cells (opened peak set), or in both *Daxx*$^{+/+}$*;RosaCre*^{ER} and *Daxx*$^{F/F}$*;RosaCre*^{ER} cells (unchanged peak set) for each cell type

analyzed. Next, the closed and opened peak sets were overlaid with a set of HSC, CMP and GMP-specific enhancer peaks [18] with bedtools intersect to obtain enhancer specific sets that closed or opened upon Daxx KO. In addition, enhancer overlapping accessible regions were analyzed for their overlap (at least 20% of their sequence) with known endogenous retroviral elements (ERVs) extracted from the mm10 repeatmasker file. For the Ingenuity canonical pathway analysis (Qiagen) enhancer specific ERV-overlapping closed and opened peak sets were first annotated with annotatePeaks.pl to identify the closest promoter RefSeq ID. After removal of duplicated IDs, IDs from the closed and opened enhancer specific peak sets for each cell type were combined in one file. To allow for directionality in the analysis and calculation of the Activation Z-score, IDs from the closed peak set were denoted a -1 and IDs from the opened peak set were denoted as +1. The files were uploaded to the Ingenuity platform (Qiagen) and analyzed with default settings. Enriched transcription factor motifs were identified in ERV-overlapping enhancer peaks open in Daxx KO in HSCs using the findMotifsGenome.pl script from HOMER and mm10 as genome.

Differentially accessible regions for integration with CUT&Tag data were called by using diffreps[70] with a window size of 600 bp and p-value cut off < 0.0001. The identified regions were annotated using the region_analysis package based on the Ensembl mm10 gene annotation. Regions were separated into promoter (annotated as “ProximalPromoter” or “Promoter1K”) and distal regions (> +/- 1 kbp from TSS). Next, differential sites were overlaid with a set of HSC, CMP and GMP-specific enhancer peaks [18] with bedtools intersect to obtain enhancer specific sets that closed or opened upon Daxx KO. In addition, enhancer overlapping accessible regions were analyzed for their overlap (at least 20% of their sequence) with known endogenous retroviral elements (ERVs) extracted from the mm10 repeatmasker file.

<u>Association of ATAC-seq data with TERRA binding sites</u>

Raw data of TERRA ChIRT-seq data and the associated input data were downloaded from the Sequencing Read Archive (SRR2062971 and SRR2062968). After removal of adapter sequences with Cutadapt (Version 1.13), paired-end reads were aligned to the mouse reference genome (mm10) using the software STAR (options: --alignIntronMax 1 –alignMatesGapMax 1800 –alignEndsType EndToEnd –seedSearchStartLmax 30). Only properly paired, uniquely mapping reads were kept for further analysis and PCR duplicates were removed with MarkDuplicates from the Picard tools. Peaks were called by MACS2 software (Version 2.1.1.20160309) using normalization to input and filtered by 10-fold enrichment. Shifted alignment files from the corresponding biological replicates for *Daxx*$^{+/+}$*;RosaCre*^{ER} or *Daxx*$^{F/F}$*;RosaCre*^{ER} LT-HSC, CMP, or GMP cells were merged and converted from bed format to bam files using the bedToBam tool from the bedtools software package. Each of the three cell types was analyzed separately. Bam

files were converted to coverage plot files using bamCoverage from deepTools (Version 3.0.2) with a bin size of 1 and option --normalizeUsingRPKM. The resulting normalized coverage tracks were used to compute coverage within +/-3000 bp of the identified TERRA peak centers (computeMatrix from deepTools package with bin size of 1). Finally, profile plots were created using the computed score matrix using the tool plotProfile from the deepTools package.

Bioinformatic analysis of RNA-seq data

Pre-processing, mapping and filtering of RNA-seq data

FastQC was used to assess sequencing read quality. Reads were quality and adapter trimmed using Cutadapt. Only properly paired reads with minimum length of 30 bp were kept for further analysis. Reads were mapped to the mouse genome (mm10, GENCODE release M14) using STAR (with options --outSAMstrandField intronMotif and --outFilterIntronMotifs RemoveNoncanonical) with default parameters and only uniquely mapping reads were selected for further analysis. Ribosomal and mitochondrial reads were removed using modified scripts from the PORT pipeline (https://github.com/itmat/Normalization). Coverage plots for all uniquely mapped reads were computed using bamCoverage from the deepTools package with a bin size of 1 and the option --normalizeUsingRPKM to account for differences in total number of reads of each library. SAM files were converted to BAM files using samtools view and BAM files were sorted by coordinate with samtools sort.

Creation of non-overlapping gene annotations

Annotations were based on the comprehensive gene annotation file of the GENCODE Release M14 (mm10). The downloaded GTF file was loaded into R (Version 3.4.0; R Foundation for Statistical Computing, Vienna, Austria) and converted into a TranscriptDb object with the makeTranscriptDbFromGFF tool in the Bioconductor package GenomicFeatures (Version 1.28.3). From the TranscriptDb object, all annotated Ensembl genes and their exons were obtained using exonsBy (by="gene"). Ensembl gene ids were replaced by official gene symbols using biomaRt (Version 2.32.1). Genes with several Ensembl gene IDs were combined into one record. For each duplicated gene, overlapping exons were combined into single exons. Thus, genes were defined as the sequence between the first base of the first exon and the last base of the last exon. Furthermore, regions shared by overlapping genes were removed as we worked with a non-stranded RNA-seq library and wanted to count only reads mapping to one gene.

Differential gene expression analysis

The mapped and filtered RNA-seq reads were counted using a custom R script including the R packages Rsamtools (Version 1.28.0), GenomicFeatures and GenomicAlignments (Version

1.12.2). Briefly, sorted BAM files were loaded into R using readGAlignmentPairs and the number of reads mapping to genes was computed using findOverlaps (with options type=”within” and ignore.strand=TRUE) and countSubjectHits. Genes were analyzed for differential expression in *Daxx*$^{+/+}$*;Mx1Cre*$^{+/-}$ GMP compared to *Daxx*$^{+/+}$*;Mx1Cre*$^{+/-}$ KLS (differentiation), in *Daxx*$^{F/F}$*;Mx1Cre*$^{+/-}$ KLS compared to *Daxx*$^{+/+}$*;Mx1Cre*$^{+/-}$ KLS (KLS) and in *Daxx*$^{F/F}$*;Mx1Cre*$^{+/-}$ GMP compared to *Daxx*$^{+/+}$*;Mx1Cre*$^{+/-}$ GMP (GMP) using the R package DESeq2 (Version 1.16.1) and identified as differentially expressed if the FDR-adjusted p-value was smaller than 0.05. Differentially expressed genes (DEG) associated with differentiation were analyzed for overlaps with DEG associated with Daxx KO in KLS cells. Principle component analysis plots, Vulcano plots and heatmaps were plotted using R. Transcriptional regulators and marker genes of blood cell differentiation were selected based on literature search. Ingenuity Pathway Analysis (IPA) was used to identify canonical pathways, diseases and functions, as well as upstream differentially expressed transcriptional regulators altered due to Daxx loss. Pathways, diseases and functions were restricted to the top 5 enriched or depleted categories associated with immune cell function (pathways) or the hematologic system (diseases and functions).

Repeat element differential expression analysis

For the repeat element differential expression analysis trimmed and properly paired reads were realigned against the mouse genome (mm10) using STAR with settings that allows mapping of reads at up to 100 different genomic locations (options used: --outFilterMultimapNmax 100 and --winAnchorMultimapNmax 100). Next, reads mapping to the mitochondrial genome were excluded and aligned reads were sorted by read name. To obtain aligned read counts for mouse repeat elements TEtranscripts from the TEToolkit (Version 2.0.2) was used and the required GTF file containing mapping information for repeat elements was downloaded from the Hammell lab website (http://hammelllab.labsites.cshl.edu/software/#TEToolkit). The resulting count matrix was loaded and differentially expressed repeat elements were identified with DESeq2 as described above for differentially expressed genes. Normalized counts for each TE subtype were determined by determining the total number reads for each subtype within each sample, dividing by the total number of TE reads for each sample and multiplying with 1,000,000.

Bioinformatic analysis of CUT&Tag data

Reads were aligned against the mouse genome (mm10, GENCODE release M14) using bowtie2 (Version 4.8.5 with options --end-to-end --very-sensitive --no-unal --no-mixed --no-discordant --phred33 -I 10 and -X 700). Only high quality (low number of mismatches) reads and reads not mapping to the mitochondrial genome were kept for further analysis. Filtering was done with samtools (Version 1.4.1). Coverage plots for all filtered reads were computed using bamCoverage

from the deepTools package with a bin size of 1 and the option --normalizeUsingRPKM to account for differences in total number of reads of each library. Sites differentially enriched in H3.3, H3K27ac, H3K9me3, H3K27me3 or Pu.1 in Daxx KO vs. WT were called by using diffreps[70] with a window size of 600 bp and p-value cut off < 0.0001. Only differential sites with log2 fold change >+/-2.0 were kept for further analysis. The identified regions were annotated using the region_analysis package based on the Ensembl mm10 gene annotation. Regions were separated into promoter (annotated as "ProximalPromoter" or "Promoter1K") and distal regions (> +/- 1 kbp from TSS). Next, differential sites were overlaid with a set of HSC, CMP and GMP-specific enhancer peaks [18] with bedtools intersect to obtain enhancer specific sets. In addition, enhancer overlapping differential regions were analyzed for their overlap (at least 20% of their sequence) with known endogenous retroviral elements (ERVs) extracted from the mm10 repeatmasker file.

18 Lara-Astiaso, D. *et al.* Immunogenetics. Chromatin state dynamics during blood formation. *Science* **345**, 943-949, doi:10.1126/science.1256271 (2014).

70 Shen, L. *et al.* diffReps: detecting differential chromatin modification sites from ChIP-seq data with biological replicates. *PLoS One* **8**, e65598, doi:10.1371/journal.pone.0065598 (2013).

Supplementary Code 1: Rscript for counting paired-end RNA-seq reads

```
#!/usr/bin/env Rscript

#Requires Rsamtools, GenomicFeatures and GenomicAlignments
library("Rsamtools")
library("GenomicFeatures")
library("GenomicAlignments")

#Load sample table to obtain sample names and bam file information
file <- "171027_sampleTableFull.txt"
sampleTable <- read.table(file,header=TRUE,sep="\t")
filenames <- paste0(sampleTable$SampleName, "_tr.f.u.s.bam")

#Load GRanges datasets for which we get counts
load("171027_mm10_Genes_Exons_Introns.rda")

#Create count matrices to store counts into
counts.g <- matrix(nrow=length(genes1),ncol=length(filenames))
colnames(counts.g) <- sampleTable$SampleName
rownames(counts.g) <- names(genes1)
read.stats <- matrix(nrow=4,ncol=length(filenames))
rownames(read.stats) <- c("Total","MappingGenes","MappingWithinGenes","Non-overlapping")
colnames(read.stats) <- sampleTable$SampleName
save(counts.g, read.stats,file="171027_Counts_Statistics.rda")
for(i in 1:length(filenames)) {
	load("171027_Counts_Statistics.rda")
	print(filenames[i])
	bam <- readGAlignmentPairs(filenames[i], use.names=TRUE)
	read.stats[1,i] <- length(bam)
	ov <- findOverlaps(bam,genes1, ignore.strand=TRUE)
	read.stats[2,i] <- length(unique(queryHits(ov)))
	ov.g <- findOverlaps(bam, genes1, type="within",ignore.strand=TRUE)
	read.stats[3,i] <- length(unique(queryHits(ov.g)))
	reads_to_keep <- which(countQueryHits(ov.g)==1L)
	read.stats[4,i] <- length(reads_to_keep)
	ov.g <- ov.g[queryHits(ov.g) %in% reads_to_keep]
	counts.g[,i] <- countSubjectHits(ov.g)
	save(counts.g, read.stats,file="171027_Counts_Statistics.rda")
}

#Save count matrices and statistics
save(counts.g, read.stats,file="171027_Counts_Statistics.rda")
```

Supplementary Code 2: Rscript for counting single-end RNA-seq reads

```
#!/usr/bin/env Rscript

#Requires Rsamtools, GenomicFeatures and GenomicAlignments
library("Rsamtools")
library("GenomicFeatures")
library("GenomicAlignments")

#Load sample table to obtain sample names and bam file information
file <- "SE_sampletable.txt"
sampleTable <- read.table(file,header=TRUE,sep="\t")
filenames <- paste0(sampleTable$SampleName, ".a.f.s.bam")

#Load GRanges datasets for which we get counts
load("/vol002/Jenny/RNAseq/190530_GeneAnnotationForCounting.rda")

#Create count matrices to store counts into
counts.g <- matrix(nrow=length(geneinfo),ncol=length(filenames))
colnames(counts.g) <- sampleTable$SampleName
rownames(counts.g) <- names(geneinfo)
read.stats <- matrix(nrow=4,ncol=length(filenames))
rownames(read.stats) <- c("Total","MappingGenes","MappingWithinGenes","Non-overlapping")
colnames(read.stats) <- sampleTable$SampleName
save(counts.g, read.stats,file="201122_Counts_Statistics.rda")
for(i in 1:length(filenames)) {
	load("201122_Counts_Statistics.rda")
	print(filenames[i])
	bam <- readGAlignments(filenames[i], use.names=TRUE)
	read.stats[1,i] <- length(bam)
	ov <- findOverlaps(bam,geneinfo, ignore.strand=TRUE)
	read.stats[2,i] <- length(unique(queryHits(ov)))
	ov.g <- findOverlaps(bam, geneinfo, type="within",ignore.strand=TRUE)
	read.stats[3,i] <- length(unique(queryHits(ov.g)))
	reads_to_keep <- which(countQueryHits(ov.g)==1L)
	read.stats[4,i] <- length(reads_to_keep)
	ov.g <- ov.g[queryHits(ov.g) %in% reads_to_keep]
	counts.g[,i] <- countSubjectHits(ov.g)
	save(counts.g, read.stats,file="201122_Counts_Statistics.rda")
}

#Save count matrices and statistics
save(counts.g, read.stats,file="201122_Counts_Statistics.rda")
```
