# Supplementary material for: Aberrant chromatin landscape following loss of the H3.3 chaperone Daxx in haematopoietic precursors leads to Pu.1-mediated neutrophilia and inflammation
Source: Nat Cell Biol. 2021 Dec 7;23(12):1224–39. doi: 10.1038/s41556-021-00774-y (PMC8683376; doi:10.1038/s41556-021-00774-y)
Supplement: Supplementary file 2 — Reporting Summary [file 41556_2021_774_MOESM2_ESM.pdf]

nature research

Corresponding author(s): Paolo Salomoni

Last updated by author(s): Aug 31, 2021

Reporting Summary

Nature Research wishes to improve the reproducibility of the work that we publish. This form provides structure for consistency and transparency in reporting. For further information on Nature Research policies, see Authors & Referees and the Editorial Policy Checklist.

Statistics

For all statistical analyses, confirm that the following items are present in the figure legend, table legend, main text, or Methods section.

n/a	Confirmed	
☐	☒	The exact sample size (*n*) for each experimental group/condition, given as a discrete number and unit of measurement
☐	☒	A statement on whether measurements were taken from distinct samples or whether the same sample was measured repeatedly
☐	☒	The statistical test(s) used AND whether they are one- or two-sided<br>*Only common tests should be described solely by name; describe more complex techniques in the Methods section.*
☒	☐	A description of all covariates tested
☐	☒	A description of any assumptions or corrections, such as tests of normality and adjustment for multiple comparisons
☐	☒	A full description of the statistical parameters including central tendency (e.g. means) or other basic estimates (e.g. regression coefficient) AND variation (e.g. standard deviation) or associated estimates of uncertainty (e.g. confidence intervals)
☐	☒	For null hypothesis testing, the test statistic (e.g. *F*, *t*, *r*) with confidence intervals, effect sizes, degrees of freedom and *P* value noted<br>*Give P values as exact values whenever suitable.*
☒	☐	For Bayesian analysis, information on the choice of priors and Markov chain Monte Carlo settings
☒	☐	For hierarchical and complex designs, identification of the appropriate level for tests and full reporting of outcomes
☒	☐	Estimates of effect sizes (e.g. Cohen's *d*, Pearson's *r*), indicating how they were calculated

Our web collection on statistics for biologists contains articles on many of the points above.

Software and code

Policy information about availability of computer code

Data collection	Primary processing of ATAC-seq data was performed with CASAVA (1.8.4), FastQC (0.11.5), Trimmomatic (0.36 with options -phred33, seedMismatches=2, palindromeClipThreshold=30, simpleClipThreshold=10 and option MINLEN:36), STAR (2.5.3 with option --alignIntronMax 1 and --alignMatesGapMax 1800), samtools (1.4.1), MarkDuplicates (option REMOVE_DUPLICATES=true) from Picard tools (2.9.2) and bedtools (2.25.0)<br>Primary processing of RNA-seq data was performed with CASAVA (1.8.4), FastQC (0.11.5), Cutadapt (1.13), STAR (2.5.3), samtools (1.4.1) and the PORT pipeline (https://github.com/itmat/Normalization).<br>CUT&Tag was performed on HSPCs (Lineage-negative, c-Kit-positive) isolated by MACS. Primary processing was done with CASAVA (1.8.4), FastQC (0.11.5), bowtie2 (4.8.5), samtools (1.4.1) and bedtools (2.25.0).<br>A detailed description can be found in the Methods section and all tools and datasets used are listed in the Supplementary Information.
Data analysis	ATAC-seq data was analyzed using the ENCODE ATAC-seq analysis pipeline (0.3.4). Resulting peak files were annotated with the HOMER software package (4.9.1) as described in the Methods section. Genome Browser coverage plots and profile plots were created with deepTools (3.0.2) and Pathway analysis was performed with the Ingenuity Pathway analysis pipeline from Qiagen.<br>Statistical analysis was run in R (3.4.0) and GraphPad Prism (7.03), which were also used for plotting. Gene set enrichment analysis (GSEA) was performed with the GSEA program provided by the Broad Institute as described in the Methods section. Differential transposable element RNA expression was determined by applying the TEToolkit (2.0.2) as described in the Methods section. Differential expression analysis employed the R or Bioconductor packages GenomicFeatures (1.28.3), biomaRt (2.32.1), Rsamtools (1.28.0), GenomicAlignments (1.12.2) and DESeq2 (1.16.1). Genomic regions differentially enriched in H3.3, H3K27ac, H3K9me3, H3K27me3 or Pu.1 were called by using diffreps. Differential regions were annoted using the region_analysis package from diffreps.<br>FACS data was analyzed with FlowJo (10.6.1).

For manuscripts utilizing custom algorithms or software that are central to the research but not yet described in published literature, software must be made available to editors/reviewers. We strongly encourage code deposition in a community repository (e.g. GitHub). See the Nature Research guidelines for submitting code & software for further information.

Data

Policy information about availability of data

All manuscripts must include a data availability statement. This statement should provide the following information, where applicable:

- Accession codes, unique identifiers, or web links for publicly available datasets
- A list of figures that have associated raw data
- A description of any restrictions on data availability

All sequencing data related to the manuscriptthat support the findings of this study can be found at Gene Expression Omnibus under the accession number GSE119309. An overview of the genomics studies run as part of this study can be found in Supplementary Table 11. Previously published sequencing data that were reanalyzed here are available under the accession codes GSE60101 and GSE79180 (SRR2062971 and SRR2062968). GTF and FASTA files used for Bioinformatics analysis (mm10, GENCODE release M14) can be downloaded from GENCODE (https://www.gencodegenes.org/mouse/release_M14.html). Source Data for Figs. 1-8 and Extended Data Figs. 1-8 are available online. All other data supporting the findings of this study are available from the corresponding author on reasonable request.

Field-specific reporting

Please select the one below that is the best fit for your research. If you are not sure, read the appropriate sections before making your selection.

☒ Life sciences ☐ Behavioural & social sciences ☐ Ecological, evolutionary & environmental sciences

For a reference copy of the document with all sections, see nature.com/documents/nr-reporting-summary-flat.pdf

Life sciences study design

All studies must disclose on these points even when the disclosure is negative.

Sample size	Preliminary experiments were run to test for biological outcome. On the basis of the statistical anylsis of this data, the sample size required to achieve statistically significant results was calculated using Power Analysis. Sample sizes are noted in each case.
Data exclusions	Data was partially excluded from the analysis of cytokine concentrations using Legendplex Mouse Inflammation Panel (Biolegend, 740446) and Mouse HSC Panel (Biolegend, 740677). The kits include a standard that is used for reference in the LEGENDplexTM Data Analysis Software. For several cytokines the analyzed concentrations of our samples were either not detectable or outside the analyzable range of the standard curve. In this case, single samples were excluded. Statistical analysis were run only if at least three biological replicates were available. The cytokines included in the kit that did not fall into the analyzable range of the standard curve are not shown as data in the manuscript.
Replication	Experiments run on the same day always included wild-type and knock-out mice. For most experiments (except for ATAC-seq, RNA-seq and CUT&Tag samples) included at least one replicate experiment run on a different date. The attempts of replication were successful and no experiment was excluded. All samples and mice were handled following the same protocols. Due to limitations in animals (numbers approved by authorities and COVID pandemic related restrictions for animal breeding) and complexity of required cell extraction, cell sorting and genomics library preparation, genomics-based experiments were run with biological replicates but without repetition at different dates.
Randomization	Sample allocation was random and mice for experiments were randomly chosen based on availability of the right genotypes.
Blinding	The investigators were not blinded to the mouse group allocation nor when assessing the outcome of experiments to ensure monitoring of mice for occurrence of skin lesions or signs of leukemia (in this case mice needed to be sacrificed). Blinding for analysis was not necessary as total cells are isolated from the different organs and cells are randomly assessed within the flow cytometer. To avoid bias during cell sorting (which are randomly assessed) the same sorting gates are used for all samples run on the same date.

Reporting for specific materials, systems and methods

We require information from authors about some types of materials, experimental systems and methods used in many studies. Here, indicate whether each material, system or method listed is relevant to your study. If you are not sure if a list item applies to your research, read the appropriate section before selecting a response.

Materials & experimental systems

n/a	Involved in the study	
☐	☒	Antibodies
☒	☐	Eukaryotic cell lines
☒	☐	Palaeontology
☐	☒	Animals and other organisms
☐	☒	Human research participants
☒	☐	Clinical data

Methods

n/a	Involved in the study	
☐	☒	ChIP-seq
☐	☒	Flow cytometry
☒	☐	MRI-based neuroimaging

Antibodies

Antibodies used	Antibodies used are described in detail in Supplementary Table 12.
Validation	All antibodies used are commercially available antibodies and detailed validation information of each antibody can be found from the manufacturer, whose information can be found in Supplementary Table 12.

Animals and other organisms

Policy information about studies involving animals; ARRIVE guidelines recommended for reporting animal research

Laboratory animals	C57BL/6N Daxx F/F mice were crossed with C57BL/6J Csf1r-Cre mice (JAX 029206), C57BL/6J Mx1Cre mice (JAX 003556) or C57BL/6J Rosa26CreERT2 mice (JAX 008463). The C57BL/6N Hira conditional KO (HiraF/F) mice were crossed with C57BL/6J Mx1Cre mice (JAX 003556). The C57BL/6J Pu.1 conditional KO (Pu.1F/F; JAX 006922) were crossed with C57BL/6 DaxxF/F;Mx1Cre mice. For BM chimera experiments C57BL/6J CD45.1 mice were used (JAX 002014).<br>Activation of Cre in Daxx;RosaCreER mice was induced by administration of 80 mg/kg tamoxifen in corn oil (Sigma-Aldrich) via oral gavage on five consecutive days or by administration of 100 mg/kg tamoxifen in corn oil via i.p. injection on five consecutive days, two days break, followed by 2 more consecutive days. For induction of Cre activation Daxx KO, Hira KO and Daxx Pu.1 double KO Mx1Cre mice received intraperitoneal injection with 300 µg polyinosinic–polycytidylic acid (pI:C, Sigma-Aldrich) three times every other day. Mice were usually treated between 5-11 weeks of age. Mice of both genders were used in this study. The n value in Figure legends reflects the number of mice analyzed in each experiment.
Wild animals	The study did not involve wild animals.
Field-collected samples	The study did not involved field-collected samples.
Ethics oversight	All mouse experiments were performed in compliance with the UK and German Law for Welfare of Laboratory Animals and were approved by the Home Office in the UK (Project license 70-8240) as well as by the Landesamt fuer Natur, Umwelt und Verbraucherschuty (LANUV) of Nordrhein-Westfalen, Germany (Project license 84-02.04.2016.A486).

Note that full information on the approval of the study protocol must also be provided in the manuscript.

Human research participants

Policy information about studies involving human research participants

Population characteristics	We included H&E stainings of skin from patients with Pyoderma gangraenosum in our study.
Recruitment	The text below is adapted from an email from Prof Wenzel that I have forwarded to the Editorial office.<br><br>The histological picture of the PG lesions has been taken from a skin sample that had been taken from a PG patient within the normal diagnostic procedure. This routine procedure includes the preparation of H&E samples. Following German law is is fine to make pictures from these skin samples (-> it is not possible to recognize the patient and no additional investigations have been done with this sample), so a "patient consent" is not needed. This is also, as far as I know, in accordance with the Helsinki Ethical guidelines. However, the patient has given his informed consent to perform the skin biopsy within the diagnostic procedure.
Ethics oversight	Prof Wenzel has ethical Votum from the University of Bonn that allows to use skin material that had been taken for diagnostic proposes for research afterwards (BN090/04) in principle. but Prof Wenzel doesn´t have a specific consent of this patient to make a picture of his H&E slide, because it was not needed to do this.

Note that full information on the approval of the study protocol must also be provided in the manuscript.

ChIP-seq

Data deposition

☒ Confirm that both raw and final processed data have been deposited in a public database such as GEO.

☒ Confirm that you have deposited or provided access to graph files (e.g. BED files) for the called peaks.

Data access links<br>*May remain private before publication.*	Raw sequencing data was deposited in GEO under accession number GSE119309.
Files in database submission	See Supplementary Table 11
Genome browser session<br>(e.g. UCSC)	

https://genome.ucsc.edu/s/jentiger82/ATACseq_LTHSC_Daxx_ROSACreERmice;
https://genome.ucsc.edu/s/jentiger82/CUT%26Tag_ATACseq_Daxx_Mx1Cre_3wpi;
https://genome.ucsc.edu/s/jentiger82/CUT%26Tag_Daxx_Mx1Cre_8wpi;
https://genome.ucsc.edu/s/jentiger82/CUT%26Tag_Daxx_Mx1Cre_24wpi

Methodology

Replicates	2-3 biological replicates
Sequencing depth	about 10 million paired-end reads per sample
Antibodies	Antibodies against H3.3, Pu.1, H3K9me3, H3K27me3 and H3K27ac. Details can be found in Supplementary Table 12.
Peak calling parameters	Online Methods
Data quality	Online Methods
Software	Online Methods

Flow Cytometry

Plots

Confirm that:

☒ The axis labels state the marker and fluorochrome used (e.g. CD4-FITC).

☒ The axis scales are clearly visible. Include numbers along axes only for bottom left plot of group (a 'group' is an analysis of identical markers).

☒ All plots are contour plots with outliers or pseudocolor plots.

☒ A numerical value for number of cells or percentage (with statistics) is provided.

Methodology

Sample preparation	Peripheral blood samples were collected from the tail vein of living mice. Bone marrow (BM) was isolated from femur and tibia of the hind legs and spleen cells from spleen of euthanized mice. Spleens were homogenized into single cells suspensions through a 70 µm cell strainer. Unless red blood cells (RBC) were the cell population to be analyzed, RBC lysis was performed for 10 min in ammonium chloride and washed twice prior to antibody staining. Haematopoietic progenitor cells (HPC) were isolated from BM using the EasySep™ Mouse Hematopoietic Progenitor Cell Isolation Kit (STEMCELL Technologies) according to manufacturer`s instructions.
Instrument	Flow cytometry analysis was performed on a BD LSR Fortessa, BD FACS Symphony, BD FACS Celesta, BD FACS Aria III or Beckman Coulter Gallios machine.<br>Cell sorting was performed on a BD FACS Aria III or BD FACS Aria Fusion machine using a 70 µm or 100 µm nozzle
Software	Collection of data on BD FACS machines was performed by using BD FACSDIVA software. Collection of data on Beckman Coulter Gallios machines was performed by using Beckman Coulter KALUZA software. Data was analyzed using FlowJo (FlowJo, LLC) software.
Cell population abundance	For RNA sequencing CD45.2+ KLS (Lin-, CD45.2+, c-kit+, Sca1+) and GMP (Lin-, CD45.2+, c-kit+, Sca1-, CD16/32+, CD34+) populations were sorted from the bone marrow of transplanted mice. Haematopoietic stem cells (KLS) are of very low and GMP cells of low abundance in the bone marrow. Minimum 4700 cells were used for the RNA isolation. For ATAC seq sorting of CMP (Lin-, CD45.2+, c-kit+, Sca1-, CD16/32-, CD34+), GMP (Lin-, c-kit+, Sca1-, CD16/32+, CD34+) and long-term HSCs (Lin-, CD45.2+, ckit+,Sca1+, CD48-, CD150+) was performed. HSCs are of very low and GMP /CMP cells of low abundance in the bone marrow. Minimum 8000 cells were processed for ATACseq. To enrich frequency of haematopoietic stem cells in the sorting population, c-kit enrichment was performed prior to sorting of long-term HSCs.<br>To minimize the isolation of other cell populations during sorting, strict gating strategies were set based on FMO controls and unstained samples. Purity check of samples after sorting was performed by running small amounts of the sorted samples through the sorter.
Gating strategy	The typical gating strategy was exclusion of cell debris on FSC/SSC plot, followed by single cell gating on FSC-H/FSC-A plot, followed by exclusion of dead cells using live/dead cells stains propidium iodide or Aqua Zombie fixable viability dye, followed by gating on the interesting cell populations based on the fluorochrome.<br>For setting positive and negative gates, unstained controls, single stained controls or FMO controls were used.

☒ Tick this box to confirm that a figure exemplifying the gating strategy is provided in the Supplementary Information.
