# Supplementary figures and images for: Aberrant chromatin landscape following loss of the H3.3 chaperone Daxx in haematopoietic precursors leads to Pu.1-mediated neutrophilia and inflammation
Source: Nat Cell Biol. 2021 Dec 7;23(12):1224–39. doi: 10.1038/s41556-021-00774-y (PMC8683376; doi:10.1038/s41556-021-00774-y)

Original files used in Extended Data Figure 2a

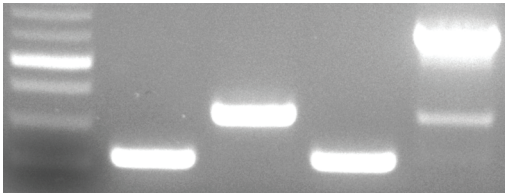

Original files used in Extended Data Figure 2d

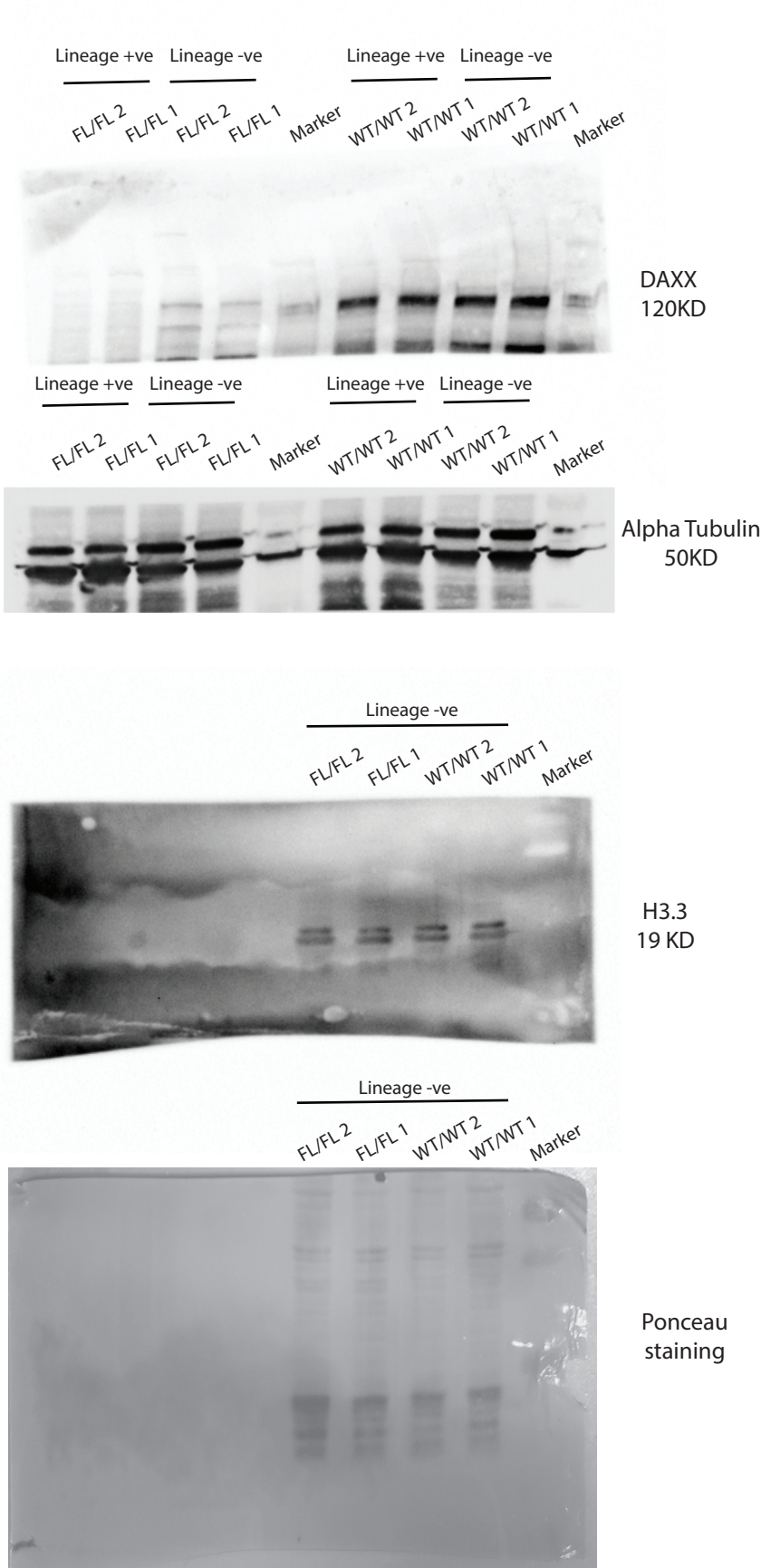

Supplement: Source Data Extended Data Fig. 2 — Unprocessed western blots and gels. [file 41556_2021_774_MOESM15_ESM.pdf]

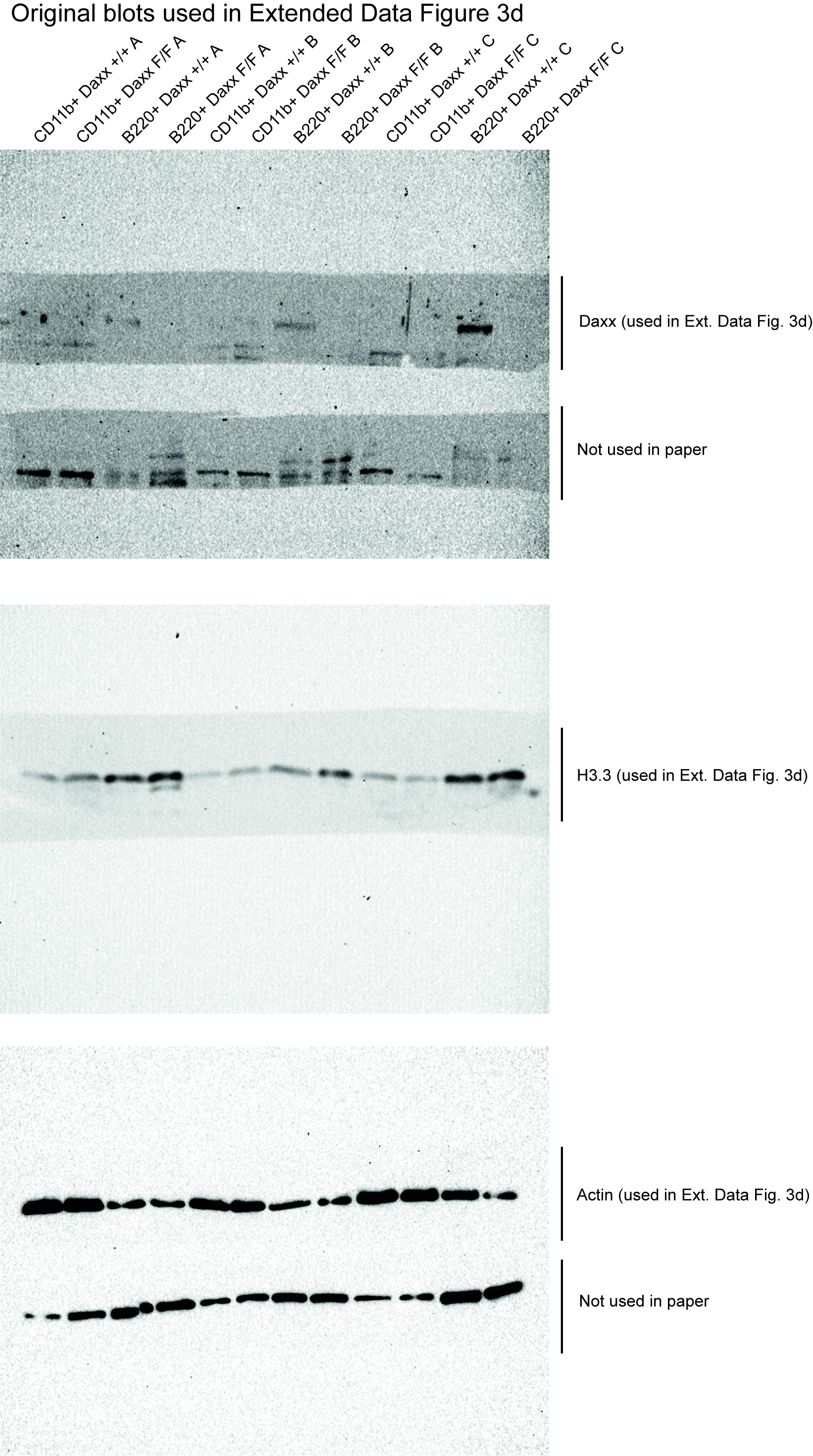

Supplement: Source Data Extended Data Fig. 3 — Unprocessed western blots and gels. [file 41556_2021_774_MOESM17_ESM.tif]

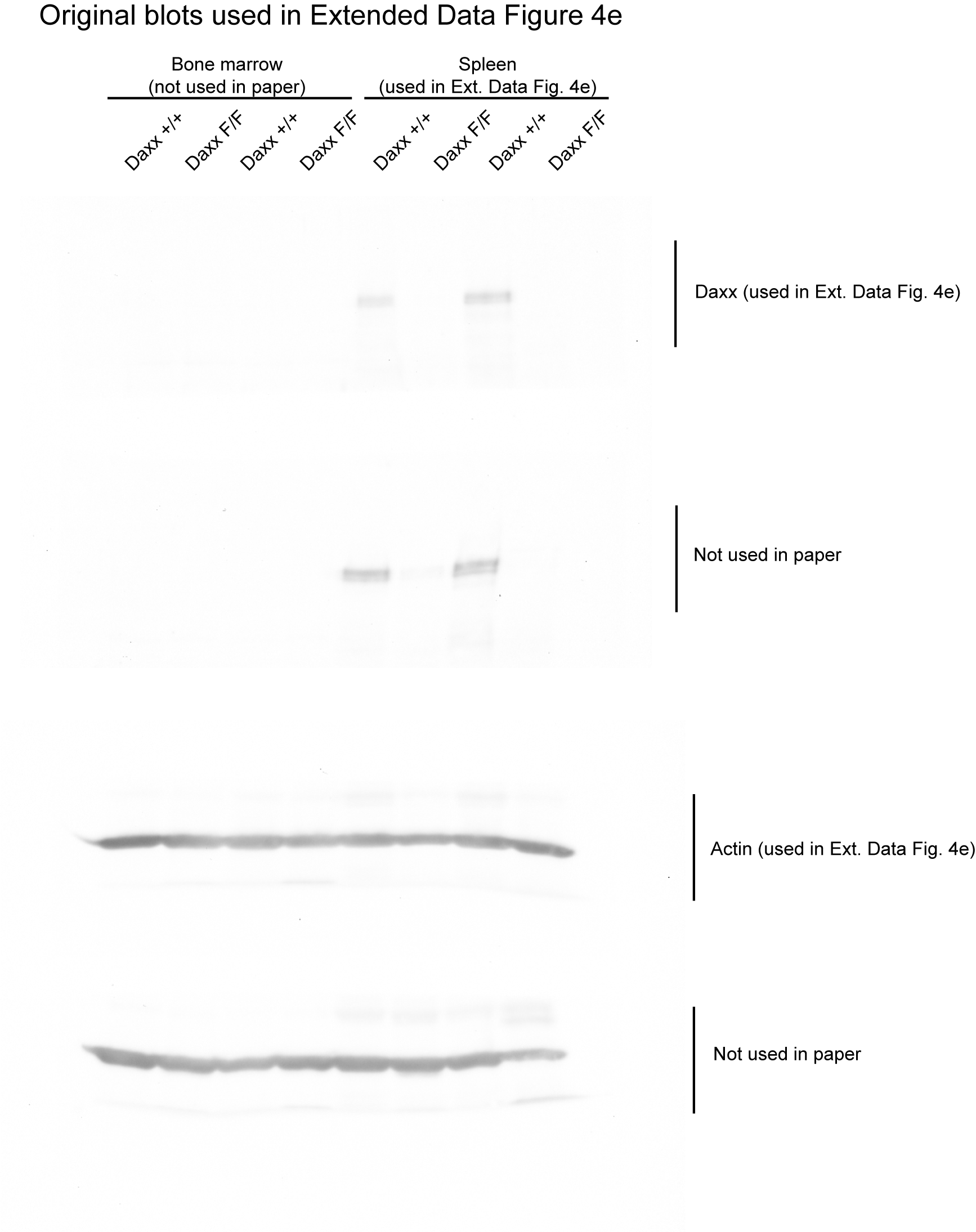

Supplement: Source Data Extended Data Fig. 4 — Unprocessed Western Blots and gels. [file 41556_2021_774_MOESM19_ESM.tif]
