# Supplementary material for: Aberrant chromatin landscape following loss of the H3.3 chaperone Daxx in haematopoietic precursors leads to Pu.1-mediated neutrophilia and inflammation
Source: Nat Cell Biol. 2021 Dec 7;23(12):1224–39. doi: 10.1038/s41556-021-00774-y (PMC8683376; doi:10.1038/s41556-021-00774-y)
Supplement: Source Data Extended Data Fig. 5 — Unprocessed western blots and gels. [file 41556_2021_774_MOESM21_ESM.pdf]

Original file used in Extended Data Figure 5a

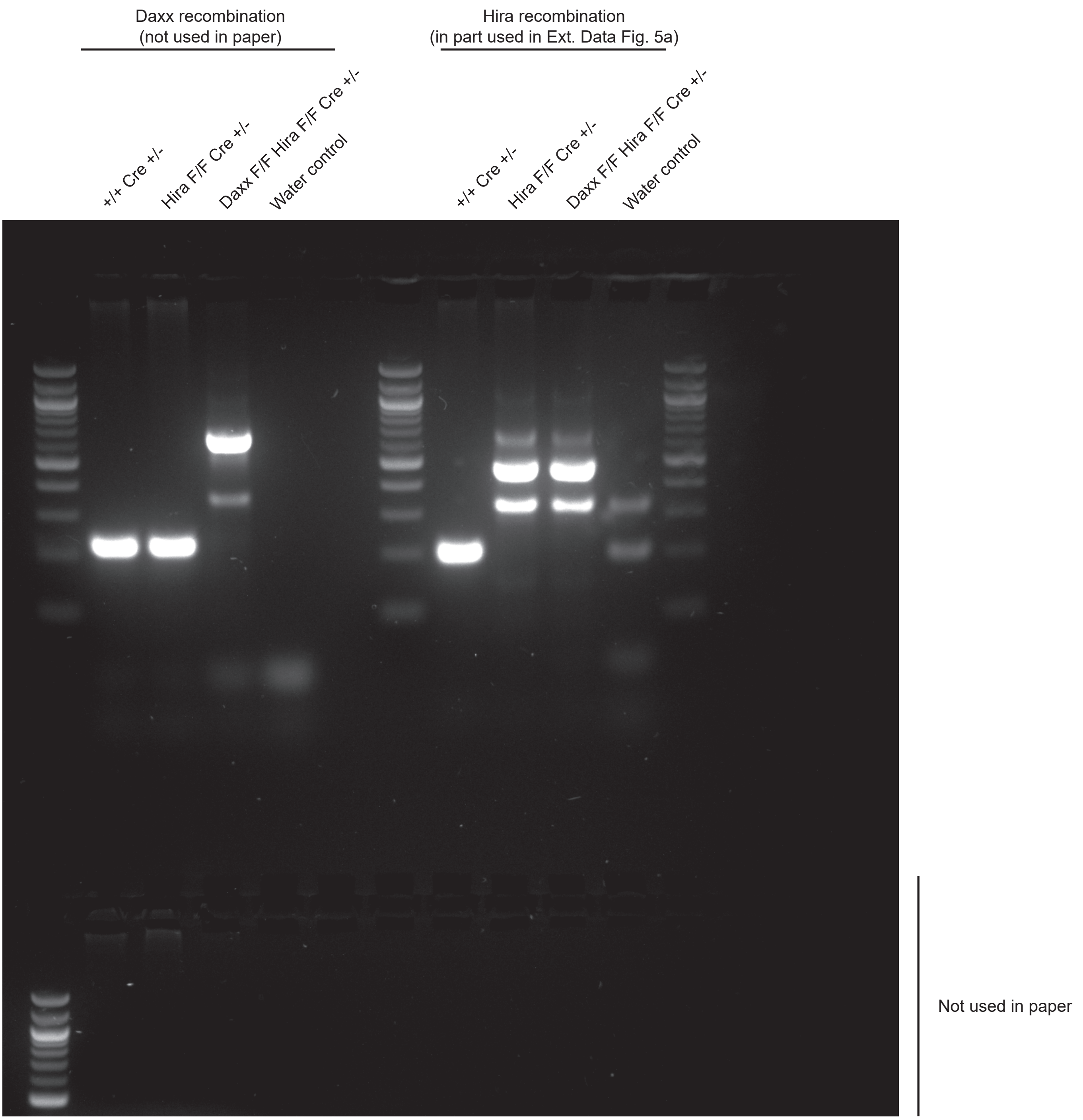

Original blots used in Extended Data Figure 5c

Total bone marrow

Used in Ext. Data Fig. 5c | Not used in paper | Used in Ext. Data Fig. 5c

+/+ Hira F/F Daxx F/F Hira F/F +/+ Hira F/F Daxx F/F Hira F/F +/+ Hira F/F Daxx F/F Hira F/F

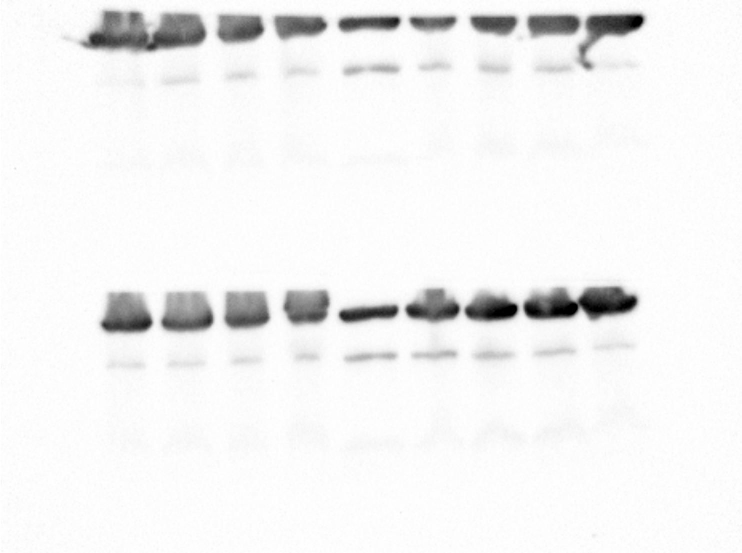

Actin
(used in Ext. Data Fig. 5c)

Not used in paper

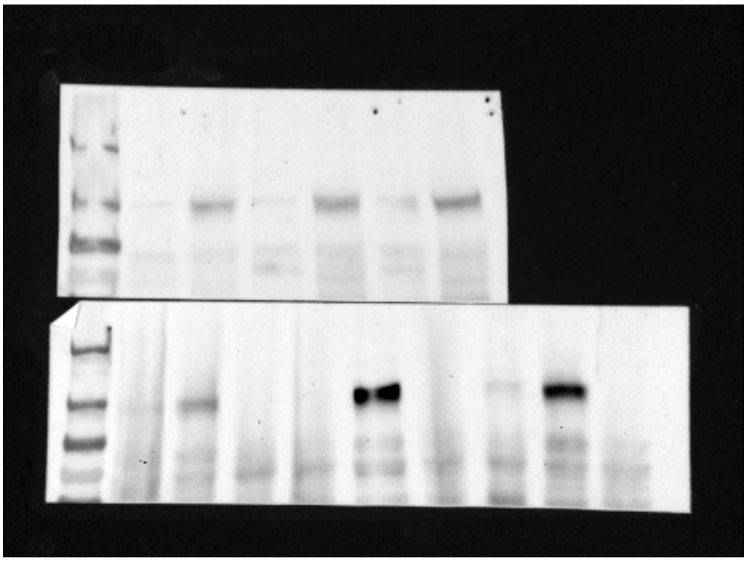

Not used in paper

Daxx
(used in Ext. Data Fig. 5c)

+/+ Hira F/F Daxx F/F Hira F/F +/+ Hira F/F Daxx F/F Hira F/F +/+ Hira F/F Daxx F/F Hira F/F

Used in Ext. Data Fig. 5c | Not used in paper | Used in Ext. Data Fig. 5c
