# Supplementary material for: Aberrant chromatin landscape following loss of the H3.3 chaperone Daxx in haematopoietic precursors leads to Pu.1-mediated neutrophilia and inflammation
Source: Nat Cell Biol. 2021 Dec 7;23(12):1224–39. doi: 10.1038/s41556-021-00774-y (PMC8683376; doi:10.1038/s41556-021-00774-y)
Supplement: Supplementary file 3 — Peer Review Information [file 41556_2021_774_MOESM3_ESM.pdf]

natureresearch

Peer Review Information

Journal: Nature Cell Biology
Manuscript Title: Aberrant chromatin landscape upon loss of the H3.3 chaperone Daxx in hematopoietic precursors leads to Pu.1–mediated neutrophilia and inflammation
Corresponding author name(s): Paolo Salomoni

Reviewer Comments & Decisions:

Decision Letter, initial version:

Dear Paolo,

Your manuscript, "Aberrant chromatin landscape upon loss of the H3.3 chaperone Daxx leads to Pu.1–mediated neutrophilia and inflammation", has now been seen by 3 referees, H3.3, pluripotency, epigenetics (referee 1); haematopoiesis (referee 2); and chromatin organisation, H3 variants (referee 3). As you will see from their comments (attached below) they find this work of potential interest, but have raised substantial concerns, which in our view would need to be addressed with considerable revisions before we can consider publication in Nature Cell Biology.

Nature Cell Biology editors discuss the referee reports in detail within the editorial team, including the chief editor, to identify key referee points that should be addressed with priority, and requests that are overruled as being beyond the scope of the current study. To guide the scope of the revisions, I have listed these points below. We are committed to providing a fair and constructive peer-review process, so please feel free to contact me if you would like to discuss any of the referee comments further.

In particular, it would be essential to address the following points:

(A) The conclusions that Daxx regulates myeloid-to-lymphoid balance during haematopoiesis and its loss leads to PU.1 induction should be supported by additional experiments, as pointed out by all referees.

Referee 1 notes:

“However, the study seems to end up a little bit suddenly after the establishment of the Pu.1 and Daxx double knockout murine system.”

"1. As much of the study relies on the Mx1Cre line to achieve inducible Daxx knockout (including physiological characterization and the following molecular experiments), is there any reason for the group to initially use RosaCreERT2+/- line for the characterization of chromatin landscape upon loss of Daxx?

2. As Pu.1 knockout partially rescues the phenotypes seen in Daxx knockout mice, has the group done any investigation to check if the altered chromatin landscape due to Daxx knockout can be restored to some extent by Pu.1 knockout?"

Referee 2 notes:

"Although the study is well written, it is not clear how Daxx deletion specifically impacts stem cells and to what extent does the Daxx deletion in HSCs contribute to neutrophilia, systemic inflammation and B cell loss."

"1. Undoubtedly, Daxx plays an important role during hematopoiesis, however the data presented in this study doesn't support a role of Daxx in HSC function.
In Figure 1, the authors present ATAC-seq data of chromatin architecture performed on HSCs CMPs and GMPs. Arguing that Daxx deleted HSCs chromatin landscape resembled that of myeloid progenitors, which contributes to myeloproliferation and suppression of lymphopoiesis. However, it is unclear whether Daxx deletion in HSCs is driving the described phenotypes presented for DaxxF/F/ Mx-1 Cre mice (myeloproliferation, neutrophil expansion and B cells loss).
In Extended Data Figure 1a, the authors describe that Daxx is highly expressed in HSCs, MEPs and B cells. However, the expression levels in other populations is similar. Thus, deletion of Daxx in LMPPs, CLPs and B220+ cells may contribute to poor B cell maintenance and survival, especially because in Extended Data Fig.2f authors report no statistically different levels of lymphoid progenitors (LMPP) upon Daxx deletion suggesting HSCs can give rise to lymphoid-prone progenitors.
As Mx1-Cre exhibits nonselective recombination in hematopoietic cells, the authors need to clarify whether the phenotypes attributed to DaxxF/F/ Mx1-Cre model is due to stem cell dysfunction or due to failure of more committed progenitors or terminally differentiated cells.

2. To prove HSC dysfunction following Daxx deletion, and differentiate the role of Daxx between HSCs and progenitors, the authors should show a more thorough analysis of HSCs and HSC function, such as analysis of quiescence and cell cycle status, self-renewal and repopulation potential following transplantation (HSC transplantation and see comment 8). To attribute the phenotypes reported following Daxx deletion to HSC dysfunction, the authors should validate their ATAC-seq and RNA-seq data in HSCs, by RT-PCR. Is the expression of myeloid and lymphoid lineage genes changed in HSCs?

3. The skin infection in the DaxxF/F/ Mx-1 Cre model raises the possibility that the abnormalities (myeloid shift in BM, splenomegaly, leukocytosis, changes in BM cytokines etc..) may be secondary to a deficit in leukocyte trafficking. The authors should evaluate the recruitment of leukocytes to a site of infection and they should also evaluate HSPC homing to the bone marrow to rule out an intrinsic adhesion/migration defect of leukocytes/HSPCs.

4. Throughout the manuscript, the authors report frequencies of different populations of blood cells including stem and progenitors. Since the bone marrow WBC cellularity is increased upon Daxx deletion (Extended Data Fig. 2d), the authors should report absolute numbers per femur. This is

especially critical when evaluating stem and progenitor populations. For example, in Extended Data Fig2f, HSC frequency doesn’t seem to change. However, ST-HSCs and LMPPs trend toward expansion, and given the increased WBC cellularity, absolute numbers of these populations may reach significance.

5. Flow cytometry plots presented in Extended Data Fig2f, suggest that LSK population is expanded upon Daxx deletion. Are there statistically different changes in LSK cells? Because the authors don’t report statistically different changes in HSCs, are any MPP population numbers changed? Given the strong myelopoiesis, one would expect changes in myeloid-biased MPPs (MPP2/MPP3).

6. The authors should justify the differences between DaxxF/F/ Mx1-Cre model and DaxxF/F / RosaCreERT2 model (Extended Data Fig.4). Does the mild phenotype in the RosaCreERT2 model stem from different recombination efficiencies in HSCs, progenitors and differentiated cells? Do elevated endogenous IFN-gamma levels (trend toward increase, Fig. 4e) affect the Mx-1 Cre model? This is important as ATAC-seq experiment presented in Fig. 1 was done using the RosaCreERT2 model and serves as basis for consequent studies, and the hematopoietic phenotype in these mice is mild, especially in regards to myeloid cell expansion. Is it possible that HSCs and progenitors would show different chromatin architecture, when Daxx is deleted using Mx1-driven Cre?

7. The authors don’t provide quantification for imaging data presented throughout the study. For example, in Fig. 3e, the authors describe that histology and immunohistochemistry analysis of the spleen revealed expansion of CD11b+ cells while F4/80+ cells did not change. The authors must quantify their immunohistochemistry data. Representative images showing focused areas of the tissue (such as in Fig. 3e, 4f), rather than showing the entire tissue (or large area of tissue) makes it hard to interpret changes in cell density or amounts.

8. It is difficult to interpret the competitive bone marrow transplantation studies presented in Extended Data Fig. 8. It is not clear what is the time line of the experiments. The authors indicate that peripheral blood data was collected at two different time points given as a range: 4-5 weeks and 8-12 weeks. For terminal data collected (reconstitution of BM and spleen) the authors indicate the data was collected 12-17 weeks post transplantation (Extended Data Fig.8e,f,g). How was the data collected? Were data points pooled from bleeding and sacrificing mice at different time points in the range indicated? This may explain the high variability and lack of significance for donor derived LSK cells and GMPs. The authors should represent peripheral blood data in a clear time course (4, 8, 12 and 16 weeks) to make a clear distinction between short term reconstitution and long-term reconstitution (attributed to HSC function). Terminal data collection for bone marrow and spleen analysis should be done at one time point, preferably 16 weeks.

9. In Extended Fig 9b, the authors show lower expression of lymphoid-associated genes and higher expression of myeloid-associated gene (Extended Fig 9b) in DaxxF/F / Mx-1 Cre LSK cells (RNAseq analysis), concluding that lymphoid-committed LSK cells should be reduced upon Dax deletion. However, In Extended Fig 2f, the authors show no significant change in LMPPs (also MPP4), which have been described in the literature as lymphoid-prone multi potent progenitors. What’s more, the data point to a trend toward expansion of LMPP frequency. Based on their data the authors can’t make this conclusion.

10. The rescue data by Pu.1 appear incomplete as a group with Pu.1 deficiency alone is missing for data interpretation. In addition, the authors would need to show improvement of the HSPC

phenotypes. Since Pu.1 is critical for myeloid cell differentiation, there is concern that Pu.1 deletion may be masking the phenotype rather than rescuing it.

11. Could the authors confirm that H3.3 expression is ablated in Daxx-deficient HSPCs?"

"14. To support the finding of reduced erythropoiesis, expansion of WBCs (presented in Extended Data Fig. 2d) and expanded Megakaryocytes (Fig. 3b), the authors should perform blood counts and at least report absolute numbers of RBC, WBCs and Platelets.

15. The authors should increase the n for data presented in Extended Data Fig.4, n of 3 is not enough to reach significance, especially given the variability of data and lack of statistical significance that is reported."

"18. The authors should justify why they performed RNAseq on LSK cells derived from chimeric mice following BM transplantation and not directly from DaxxF/F/ Mx-1 Cre mice at steady state, as these mice exhibit strong phenotypes already. Transplantation stress and active HSPC proliferation following transplantation may exacerbate the changes in gene expression upon Daxx deletion.

19. To strengthen their conclusion that PU.1 deletion partially rescues neutrophilia and B cell loss in DaxxF/F/ Mx-1 Cre mice (Extended Data Fig. 10h-k) the authors should include bone marrow data."

Referee 3:

"4/ The authors found that the master regulator of hematopoiesis PU.1/Spi-1, plays a major role in changes upon DAXX loss due to an overall higher expression of its target genes. They showed that PU.1/Spi-1, in absence of DAXX, is upregulated in both multipotent progenitors KLS and granulocyte-monocyte progenitors (GMPs). However, it is not clear how PU.1/Spi-1 itself is upregulated in absence of DAXX. For example, what are the the data obtained by ATAC-seq at this gene and its regulatory elements ?

5/ Related to Figure 4: Approximately 15% of DAXX f/f mice developed skin lesions (this corresponds to how many mice?). Due to this low percentage, it is perhaps difficult to conclude that "DAXX loss leads to systemic inflammation and neutrophilic skin disease resembling human pyoderma gangrenosum (PG)"? Do the authors have additional arguments ?

6/ The authors argue that "loss of HIRA does not markedly affect haematopoietic cell composition". However, the percentage of cells for several cell types appeared significantly changed upon HIRA loss (Extended Data Fig. 6). Perhaps these changes are less striking than upon DAXX loss, but still significant. This needs to be commented."

"8/ A cartoon summarizing the current model for the role of DAXX in the hematopoietic lineage and what happens upon loss of DAXX would help.

9/ The authors have to discuss their results in view of the recent published data from Chen et al., 2020 about the role of the HIRA chaperone in hematopoiesis."

(B) The underlying epigenetic mechanisms should be further explored, including a possible chaperone

activity of Daxx and its effects on ERVs and chromatin architecture, as noted by referees 1 and 3.

Referee 1 notes:

“3. Though the group has pointed out the altered expression profile of some ERVs, such as LTRs and TERRA IncRNA, in the Daxx knockout KLS cells, does the group have any evidence to suggest the functional significance of such altered ERV expression profile?

4. Has the group ever considered to check any histone epigenetic marks (e.g. H3K27me3) in both the Daxx knockout and Daxx/Pu.1 double knockout mice?”

Referee 3 notes:

“1/ DAXX is a H3.3 histone chaperone but no data are provided to determine whether its role in hematopoiesis is actually directly linked to its chaperone activity. It would have been important to explore this aspect. The authors found that enhancers (overlapping or not with ERVs) exhibit higher accessibility upon DAXX loss in HSCs (by ATAC-seq). Are these enhancers known to be enriched in H3.3 in HSCs?

2/ As the general repression of ERVs has been linked to H3K9me3 mark in ESCs, it would have been useful to examine whether higher accessible ERVs in DAXXf/f HSCs correlate or not with a decrease of H3K9me3. Do the enhancers non-overlapping with ERVs and displaying changes in chromatin accessibility upon DAXX loss correlate with a change in H3 post-translational modifications ?

3/ Upon DAXX loss, it is not clear to which extent changes in ATAC-seq data (Figure 1) correlate with changes in transcriptomic data (Figure 5). For example, the authors pointed out two master regulators of myelopoiesis, Myb and Cebpa, displaying higher opening of chromatin at enhancer elements in DAXX KO HSCs (Fig. 1k). However, it is unclear whether this is a general observation and if the other upregulated regulators involved in blood differentiation (shown in Extended Data Fig.9) also exhibit an opening of chromatin at enhancer elements upon DAXX loss?”

“7/ When a Western blot for DAXX is provided, H3.3 should be also shown, as the amounts of a histone variant and its chaperone have been previously reported to be co-regulated. Western blot for HIRA should be shown for HIRA KO.”

(C) All other referee concerns pertaining to strengthening existing data, providing controls, methodological details, clarifications and textual changes should also be addressed.

(D) Finally please pay close attention to our guidelines on statistical and methodological reporting (listed below) as failure to do so may delay the reconsideration of the revised manuscript. In particular please provide:

- a Supplementary Figure including unprocessed images of all gels/blots in the form of a multi-page pdf file. Please ensure that blots/gels are labeled and the sections presented in the figures are clearly indicated.

- a Supplementary Table including all numerical source data in Excel format, with data for different figures provided as different sheets within a single Excel file. The file should include source data giving rise to graphical representations and statistical descriptions in the paper and for all instances where the figures present representative experiments of multiple independent repeats, the source data of all repeats should be provided.

We would be happy to consider a revised manuscript that would satisfactorily address these points, unless a similar paper is published elsewhere, or is accepted for publication in Nature Cell Biology in the meantime.

When revising the manuscript please:

- ensure that it conforms to our format instructions and publication policies (see below and www.nature.com/nature/authors/).

- provide a point-by-point rebuttal to the full referee reports verbatim, as provided at the end of this letter.

- provide the completed Reporting Summary (found here https://www.nature.com/documents/nr-reporting-summary.pdf). This is essential for reconsideration of the manuscript will be available to editors and referees in the event of peer review. For more information see http://www.nature.com/authors/policies/availability.html or contact me.

Nature Cell Biology is committed to improving transparency in authorship. As part of our efforts in this direction, we are now requesting that all authors identified as 'corresponding author' on published papers create and link their Open Researcher and Contributor Identifier (ORCID) with their account on the Manuscript Tracking System (MTS), prior to acceptance. ORCID helps the scientific community achieve unambiguous attribution of all scholarly contributions. You can create and link your ORCID from the home page of the MTS by clicking on 'Modify my Springer Nature account'. For more information please visit please visit www.springernature.com/orcid.

This journal strongly supports public availability of data. Please place the data used in your paper into a public data repository, or alternatively, present the data as Supplementary Information. If data can only be shared on request, please explain why in your Data Availability Statement, and also in the correspondence with your editor. Please note that for some data types, deposition in a public repository is mandatory - more information on our data deposition policies and available repositories appears below.

Please submit the revised manuscript files and the point-by-point rebuttal to the referee comments using this link:

[REDACTED]

*This url links to your confidential home page and associated information about manuscripts you may have submitted or be reviewing for us. If you wish to forward this email to co-authors, please delete the link to your homepage.

We would like to receive a revised submission within six months. We would be happy to consider a

revision even after this timeframe, however if the resubmission deadline is missed and the paper is eventually published, the submission date will be the date when the revised manuscript was received.

Please do let us know if you expect extensive delays or are currently unable to perform experimental work due to the pandemic. We fully appreciate that many labs are still closed and will be able to adjust deadlines as required.

We hope that you will find our referees' comments, and editorial guidance helpful. Please do not hesitate to contact me if there is anything you would like to discuss.

Best wishes,

Christine.

Christine Weber, PhD
Senior Editor
Nature Cell Biology
E-mail: christine.weber@nature.com
Phone: +44 (0)207 843 4924

Reviewers' Comments:

Reviewer #1:
Remarks to the Author:
In this study, Hofmann et al. has demonstrated the role of Daxx in hematopoietic homeostasis maintenance. The group has extensively characterized relevant phenotypes in Daxx knockout mice. Physiologically, loss of Daxx led to a series of distortion to hematopoietic homeostasis. The key observation was the skewing towards myeloid lineage at the expense of B-cell production. Other prominent defects included spleen pathology and systemic inflammation. Interestingly, the group has investigated the loss of another H3.3 chaperone namely Hira, in which neutrophilia and inflammation phenotypes were not observed. In addition, molecular studies regarding the loss of Daxx in hematopoietic cells (e.g. hematopoietic stem cells, KLS cells) further uncovered the potential mechanisms underlying Daxx knockout phenotypes. In terms of chromatin landscape, loss of Daxx has induced a broad range of changes, such as the overall accessibility in progenitors (e.g. CMPs and GMPs) and increased opening of ERVs-overlapped enhancers. RNA sequencing evidence further suggested the shift of transcriptomic signature of progenitors towards that of the myeloid lineage upon Daxx knockout. Besides, the anti-inflammation pathways also seemed to be downregulated. After a series of extensive characterization of transcriptomic alterations (e.g. IFN signalling pathway changes, ERV-related changes), the group has narrowed down their attention to one hematopoietic master regulator, namely Pu.1. As some of its downstream targets related to myeloid differentiation have shown upregulation upon Daxx knockout, the group investigated whether loss of Pu.1 in addition of Daxx knockout could rescue the phenotypes. Indeed, the double knockout mice has shown improved physiological conditions, including reduced neutrophilia and partially restored B cell production. Together, these evidences have supported an essential role of Daxx in maintaining the homeostasis of blood lineage, in which its disruption could lead to severe physiological consequences.

Overall, this is a comprehensive study with both substantial bioinformatic analysis as well as corresponding experimental data to support. However, the study seems to end up a little bit suddenly after the establishment of the Pu.1 and Daxx double knockout murine system. Nevertheless, the reviewer still appreciates the group's efforts in uncovering the importance of Daxx in regulating hematopoietic homeostasis. At the same time, the reviewer would also like to clarify the following points:

1. As much of the study relies on the Mx1Cre line to achieve inducible Daxx knockout (including physiological characterization and the following molecular experiments), is there any reason for the group to initially use RosaCreERT2+/- line for the characterization of chromatin landscape upon loss of Daxx?

2. As Pu.1 knockout partially rescues the phenotypes seen in Daxx knockout mice, has the group done any investigation to check if the altered chromatin landscape due to Daxx knockout can be restored to some extent by Pu.1 knockout?

3. Though the group has pointed out the altered expression profile of some ERVs, such as LTRs and TERRA IncRNA, in the Daxx knockout KLS cells, does the group have any evidence to suggest the functional significance of such altered ERV expression profile?

4. Has the group ever considered to check any histone epigenetic marks (e.g. H3K27me3) in both the Daxx knockout and Daxx/Pu.1 double knockout mice?

Reviewer #2:
Remarks to the Author:
In their manuscript, Hofmann JP et al. describe a novel role for DNA methyltransferase Daxx in regulating hematopoiesis, further highlighting the role for chromatin modifiers as major regulator of HSC and progenitor function. Their results show that deletion of Daxx in hematopoietic cells enhances myelopoiesis, causing neutrophilia and increased systemic inflammation. This finding is interesting, as it may implicate Daxx as an important player in myeloproliferative disorders and/or systemic inflammatory diseases. Deletion of Daxx also suppressed B cell production, which suggests that Daxx potentially preserves chromatin integrity in stem cell, hematopoietic progenitors and terminally differentiated blood cells (all of which express Daxx). Although the study is well written, it is not clear how Daxx deletion specifically impacts stem cells and to what extent does the Daxx deletion in HSCs contribute to neutrophilia, systemic inflammation and B cell loss. The authors should address the following points to clarify this point and improve data presentation

Major points:

1. Undoubtedly, Daxx plays an important role during hematopoiesis, however the data presented in this study doesn't support a role of Daxx in HSC function.
In Figure 1, the authors present ATAC-seq data of chromatin architecture performed on HSCs CMPs and GMPs. Arguing that Daxx deleted HSCs chromatin landscape resembled that of myeloid progenitors, which contributes to myeloproliferation and suppression of lymphopoiesis. However, it is

unclear whether Daxx deletion in HSCs is driving the described phenotypes presented for DaxxF/F/ Mx-1 Cre mice (myeloproliferation, neutrophil expansion and B cells loss).
In Extended Data Figure 1a, the authors describe that Daxx is highly expressed in HSCs, MEPs and B cells. However, the expression levels in other populations is similar. Thus, deletion of Daxx in LMPPs, CLPs and B220+ cells may contribute to poor B cell maintenance and survival, especially because in Extended Data Fig.2f authors report no statistically different levels of lymphoid progenitors (LMPP) upon Daxx deletion suggesting HSCs can give rise to lymphoid-prone progenitors.
As Mx1-Cre exhibits nonselective recombination in hematopoietic cells, the authors need to clarify whether the phenotypes attributed to DaxxF/F/ Mx1-Cre model is due to stem cell dysfunction or due to failure of more committed progenitors or terminally differentiated cells.

2. To prove HSC dysfunction following Daxx deletion, and differentiate the role of Daxx between HSCs and progenitors, the authors should show a more thorough analysis of HSCs and HSC function, such as analysis of quiescence and cell cycle status, self-renewal and repopulation potential following transplantation (HSC transplantation and see comment 8). To attribute the phenotypes reported following Daxx deletion to HSC dysfunction, the authors should validate their ATAC-seq and RNA-seq data in HSCs, by RT-PCR. Is the expression of myeloid and lymphoid lineage genes changed in HSCs?

3. The skin infection in the DaxxF/F/ Mx-1 Cre model raises the possibility that the abnormalities (myeloid shift in BM, splenomegaly, leukocytosis, changes in BM cytokines etc..) may be secondary to a deficit in leukocyte trafficking. The authors should evaluate the recruitment of leukocytes to a site of infection and they should also evaluate HSPC homing to the bone marrow to rule out an intrinsic adhesion/migration defect of leukocytes/HSPCs.

4. Throughout the manuscript, the authors report frequencies of different populations of blood cells including stem and progenitors. Since the bone marrow WBC cellularity is increased upon Daxx deletion (Extended Data Fig. 2d), the authors should report absolute numbers per femur. This is especially critical when evaluating stem and progenitor populations. For example, in Extended Data Fig2f, HSC frequency doesn’t seem to change. However, ST-HSCs and LMPPs trend toward expansion, and given the increased WBC cellularity, absolute numbers of these populations may reach significance.

5. Flow cytometry plots presented in Extended Data Fig2f, suggest that LSK population is expanded upon Daxx deletion. Are there statistically different changes in LSK cells? Because the authors don’t report statistically different changes in HSCs, are any MPP population numbers changed? Given the strong myelopoiesis, one would expect changes in myeloid-biased MPPs (MPP2/MPP3).

6. The authors should justify the differences between DaxxF/F/ Mx1-Cre model and DaxxF/F / RosaCreERT2 model (Extended Data Fig.4). Does the mild phenotype in the RosaCreERT2 model stem from different recombination efficiencies in HSCs, progenitors and differentiated cells? Do elevated endogenous IFN-gamma levels (trend toward increase, Fig. 4e) affect the Mx-1 Cre model? This is important as ATAC-seq experiment presented in Fig. 1 was done using the RosaCreERT2 model and serves as basis for consequent studies, and the hematopoietic phenotype in these mice is mild, especially in regards to myeloid cell expansion. Is it possible that HSCs and progenitors would show different chromatin architecture, when Daxx is deleted using Mx1-driven Cre?

7. The authors don’t provide quantification for imaging data presented throughout the study. For example, in Fig. 3e, the authors describe that histology and immunohistochemistry analysis of the

spleen revealed expansion of CD11b+ cells while F4/80+ cells did not change. The authors must quantify their immunohistochemistry data. Representative images showing focused areas of the tissue (such as in Fig. 3e, 4f), rather than showing the entire tissue (or large area of tissue) makes it hard to interpret changes in cell density or amounts.

8. It is difficult to interpret the competitive bone marrow transplantation studies presented in Extended Data Fig. 8. It is not clear what is the time line of the experiments. The authors indicate that peripheral blood data was collected at two different time points given as a range: 4-5 weeks and 8-12 weeks. For terminal data collected (reconstitution of BM and spleen) the authors indicate the data was collected 12-17 weeks post transplantation (Extended Data Fig.8e,f,g). How was the data collected? Were data points pooled from bleeding and sacrificing mice at different time points in the range indicated? This may explain the high variability and lack of significance for donor derived LSK cells and GMPs. The authors should represent peripheral blood data in a clear time course (4, 8, 12 and 16 weeks) to make a clear distinction between short term reconstitution and long-term reconstitution (attributed to HSC function). Terminal data collection for bone marrow and spleen analysis should be done at one time point, preferably 16 weeks.

9. In Extended Fig 9b, the authors show lower expression of lymphoid-associated genes and higher expression of myeloid-associated gene (Extended Fig 9b) in DaxxF/F / Mx-1 Cre LSK cells (RNAseq analysis), concluding that lymphoid-committed LSK cells should be reduced upon Dax deletion. However, In Extended Fig 2f, the authors show no significant change in LMPPs (also MPP4), which have been described in the literature as lymphoid-prone multi potent progenitors. What's more, the data point to a trend toward expansion of LMPP frequency. Based on their data the authors can't make this conclusion.

10. The rescue data by Pu.1 appear incomplete as a group with Pu.1 deficiency alone is missing for data interpretation. In addition, the authors would need to show improvement of the HSPC phenotypes. Since Pu.1 is critical for myeloid cell differentiation, there is concern that Pu.1 deletion may be masking the phenotype rather than rescuing it.

Minor points:

11. Could the authors confirm that H3.3 expression is ablated in Daxx-deficient HSPCs?

12. In Extended Data Fig.1a, for clarification, the authors should present quantified expression data instead of a heatmap, to directly compare expression levels between the different populations presented.

13. In Fig.2a the authors state that deletion of Daxx induces increase of mature and immature granulocytes in bone sections. The authors need to provide quantification of this data and support it using flow cytometry analysis of granulocyte populations.

14. To support the finding of reduced erythropoiesis, expansion of WBCs (presented in Extended Data Fig. 2d) and expanded Megakaryocytes (Fig. 3b), the authors should perform blood counts and at least report absolute numbers of RBC, WBCs and Platelets.

15. The authors should increase the n for data presented in Extended Data Fig.4, n of 3 is not enough

to reach significance, especially given the variability of data and lack of statistical significance that is reported.

16. The authors describe expansion of neutrophils and eosinophils in the spleen (Fig. 3d) but not significant increase in Ki-67+ cell in total spleen (Extended Data Fig. 5f). Ki-67 positivity should be differentiated between splenic populations and not measures as percent from total splenocytes.

17. In Fig. 3c and Extended Data Figs. 5e, 6j and 7e the authors should outline the gates of macrophage and monocyte populations, used to identify these populations.

18. The authors should justify why they performed RNAseq on LSK cells derived from chimeric mice following BM transplantation and not directly from DaxxF/F/ Mx-1 Cre mice at steady state, as these mice exhibit strong phenotypes already. Transplantation stress and active HSPC proliferation following transplantation may exacerbate the changes in gene expression upon Daxx deletion.

19. To strengthen their conclusion that PU.1 deletion partially rescues neutrophilia and B cell loss in DaxxF/F/ Mx-1 Cre mice (Extended Data Fig. 10h-k) the authors should include bone marrow data.

20. In the discussion the authors state: "Given that the association of master TFs with chromatin modifiers has been proposed to play a direct role in cell fate reprogramming, it is plausible that Daxx could oppose the function of Pu.1 in self-renewing HSCs, thus restricting entry into differentiation". This statement should be revised because the authors provide no concise data to support this conclusion, as RNAseq analysis was done on purified LSK cells, bulk of which are comprised of MPP3 myeloid prone progenitors and MPP4, lymphoid prone progenitors. And no HSC functional data is provided to support HSC dysfunction (See major comment 2). It is possible that Dax plays a more dominant role in multi potent and committed progenitors than HSCs.

Reviewer #3:
Remarks to the Author:
Manuscript NCB-S42550-T

Aberrant chromatin landscape upon loss of the H3.3 chaperone Daxx leads to Pu.1–mediated neutrophilia and inflammation
Julia P. Hofmann, Jenny Russ, Vijay Chandrasekar, Nina Offermann, Sarah Spear, Nicola Guzzi, Simona Maida, Natalia Izotova, Kristian Händler1, Preeta Datta, Jagath Kasturiarachchi, Peter Adams, Teresa Marafioti, Tariq Enver, Jörg Wenzel, Marc Beyer, Cristian Bellodi, Joachim L. Schultze, Melania Capasso, Rachael Nimmo, Paolo Salomoni

This manuscript describes the impact of the H3.3 DAXX chaperone knockout on adult hematopoiesis in vivo in mice. The authors employed mouse models with inducible deletion of DAXX. DAXX was previously described to repress specific murine endogenous retroviral elements (ERVs) in embryonic stem cells (ESCs). Here the authors showed that loss of DAXX causes adult hematopoietic stem cells (HSCs) to aberrantly open chromatin at myeloid-restricted enhancers enriched in ERVs and/or Ets transcription factor motifs. They implicate DAXX in protection against inflammatory disease.
While the findings in this manuscript are interesting, there are several concerns and important

controls listed below that should be addressed to firm up conclusions.

1/ DAXX is a H3.3 histone chaperone but no data are provided to determine whether its role in hematopoiesis is actually directly linked to its chaperone activity. It would have been important to explore this aspect. The authors found that enhancers (overlapping or not with ERVs) exhibit higher accessibility upon DAXX loss in HSCs (by ATAC-seq). Are these enhancers known to be enriched in H3.3 in HSCs?

2/ As the general repression of ERVs has been linked to H3K9me3 mark in ESCs, it would have been useful to examine whether higher accessible ERVs in DAXXf/f HSCs correlate or not with a decrease of H3K9me3. Do the enhancers non-overlapping with ERVs and displaying changes in chromatin accessibility upon DAXX loss correlate with a change in H3 post-translational modifications ?

3/ Upon DAXX loss, it is not clear to which extent changes in ATAC-seq data (Figure 1) correlate with changes in transcriptomic data (Figure 5). For example, the authors pointed out two master regulators of myelopoiesis, Myb and Cebpa, displaying higher opening of chromatin at enhancer elements in DAXX KO HSCs (Fig. 1k). However, it is unclear whether this is a general observation and if the other upregulated regulators involved in blood differentiation (shown in Extended Data Fig.9) also exhibit an opening of chromatin at enhancer elements upon DAXX loss?

4/ The authors found that the master regulator of hematopoiesis PU.1/Spi-1, plays a major role in changes upon DAXX loss due to an overall higher expression of its target genes. They showed that PU.1/Spi-1, in absence of DAXX, is upregulated in both multipotent progenitors KLS and granulocyte-monocyte progenitors (GMPs). However, it is not clear how PU.1/Spi-1 itself is upregulated in absence of DAXX. For example, what are the the data obtained by ATAC-seq at this gene and its regulatory elements ?

5/ Related to Figure 4: Approximately 15% of DAXX f/f mice developed skin lesions (this corresponds to how many mice?). Due to this low percentage, it is perhaps difficult to conclude that “DAXX loss leads to systemic inflammation and neutrophilic skin disease resembling human pyoderma gangrenosum (PG)”? Do the authors have additional arguments ?

6/ The authors argue that “loss of HIRA does not markedly affect haematopoietic cell composition”. However, the percentage of cells for several cell types appeared significantly changed upon HIRA loss (Extended Data Fig. 6). Perhaps these changes are less striking than upon DAXX loss, but still significant. This needs to be commented.

7/ When a Western blot for DAXX is provided, H3.3 should be also shown, as the amounts of a histone variant and its chaperone have been previously reported to be co-regulated. Western blot for HIRA should be shown for HIRA KO.

8/ A cartoon summarizing the current model for the role of DAXX in the hematopoietic lineage and what happens upon loss of DAXX would help.

9/ The authors have to discuss their results in view of the recent published data from Chen et al., 2020 about the role of the HIRA chaperone in hematopoiesis.

Minor points

To help readers that are not specialists of the hematopoietic lineage, a scheme showing the hematopoietic differentiation states and in particular including all the different cell types examined in the study should be provided.

Page 7, the authors should add references for the statement "Since several reports suggest a link between neutrophilia and inflammation…". Which reports ?

Figure 1, it would be easier for the reader to indicate in the figure (and not only in the legend) that in panel g it is the ATAC-seq coverage across all chromosomes and in panel h across sex chromosomes.

GUIDELINES FOR MANUSCRIPT SUBMISSION TO NATURE CELL BIOLOGY

READABILITY OF MANUSCRIPTS – Nature Cell Biology is read by cell biologists from diverse backgrounds, many of whom are not native English speakers. Authors should aim to communicate their findings clearly, explaining technical jargon that might be unfamiliar to non-specialists, and avoiding non-standard abbreviations. Titles and abstracts should concisely communicate the main findings of the study, and the background, rationale, results and conclusions should be clearly explained in the manuscript in a manner accessible to a broad cell biology audience. Nature Cell Biology uses British spelling.

MANUSCRIPT FORMAT – please follow the guidelines listed in our Guide to Authors regarding manuscript formats at Nature Cell Biology.

TITLE – should be no more than 100 characters including spaces, without punctuation and avoiding technical terms, abbreviations, and active verbs..

AUTHOR NAMES – should be given in full.

AUTHOR AFFILIATIONS – should be denoted with numerical superscripts (not symbols) preceding the names. Full addresses should be included, with US states in full and providing zip/post codes. The corresponding author is denoted by: "Correspondence should be addressed to [initials]."

ABSTRACT AND MAIN TEXT – please follow the guidelines that are specific to the format of your manuscript, as listed in our Guide to Authors (http://www.nature.com/ncb/pdf/ncb_gta.pdf) Briefly, Nature Cell Biology Articles, Resources and Technical Reports have 3500 words, including a 150 word abstract, and the main text is subdivided in Introduction, Results, and Discussion sections. Nature Cell Biology Letters have up to 2500 words, including a 180 word introductory paragraph (abstract), and the text is not subdivided in sections.

ACKNOWLEDGEMENTS – should be kept brief. Professional titles and affiliations are unnecessary. Grant numbers can be listed.

AUTHOR CONTRIBUTIONS – must be included after the Acknowledgements, detailing the contributions of each author to the paper (e.g. experimental work, project planning, data analysis etc.). Each author should be listed by his/her initials.

FINANCIAL AND NON-FINANCIAL COMPETING INTERESTS – the authors must include one of three declarations: (1) that they have no financial and non-financial competing interests; (2) that they have financial and non-financial competing interests; or (3) that they decline to respond, after the Author Contributions section. This statement will be published with the article, and in cases where financial and non-financial competing interests are declared, these will be itemized in a web supplement to the article. For further details please see https://www.nature.com/licenceforms/nrg/competing-interests.pdf.

REFERENCES – are limited to a total of 70 for Articles, Resources, Technical Reports; and 40 for Letters. This includes references in the main text and Methods combined. References must be numbered sequentially as they appear in the main text, tables and figure legends and Methods and must follow the precise style of Nature Cell Biology references. References only cited in the Methods should be numbered consecutively following the last reference cited in the main text. References only associated with Supplementary Information (e.g. in supplementary legends) do not count toward the total reference limit and do not need to be cited in numerical continuity with references in the main text. Only published papers can be cited, and each publication cited should be included in the numbered reference list, which should include the manuscript titles. Footnotes are not permitted.

METHODS – Nature Cell Biology publishes methods online. The methods section should be provided as a separate Word document, which will be copyedited and appended to the manuscript PDF, and incorporated within the HTML format of the paper.

Methods should be written concisely, but should contain all elements necessary to allow interpretation and replication of the results. As a guideline, Methods sections typically do not exceed 3,000 words. The Methods should be divided into subsections listing reagents and techniques. When citing previous methods, accurate references should be provided and any alterations should be noted. Information must be provided about: antibody dilutions, company names, catalogue numbers and clone numbers for monoclonal antibodies; sequences of RNAi and cDNA probes/primers or company names and catalogue numbers if reagents are commercial; cell line names, sources and information on cell line identity and authentication. Animal studies and experiments involving human subjects must be reported in detail, identifying the committees approving the protocols. For studies involving human subjects/samples, a statement must be included confirming that informed consent was obtained. Statistical analyses and information on the reproducibility of experimental results should be provided in a section titled "Statistics and Reproducibility".

All Nature Cell Biology manuscripts submitted on or after March 21 2016 must include a Data availability statement as a separate section after Methods but before references, under the heading "Data Availability". . For Springer Nature policies on data availability see http://www.nature.com/authors/policies/availability.html; for more information on this particular policy see http://www.nature.com/authors/policies/data/data-availability-statements-data-

citations.pdf. The Data availability statement should include:

• Accession codes for primary datasets (generated during the study under consideration and designated as "primary accessions") and secondary datasets (published datasets reanalysed during the study under consideration, designated as "referenced accessions"). For primary accessions data should be made public to coincide with publication of the manuscript. A list of data types for which submission to community-endorsed public repositories is mandated (including sequence, structure, microarray, deep sequencing data) can be found here http://www.nature.com/authors/policies/availability.html#data.

• Unique identifiers (accession codes, DOIs or other unique persistent identifier) and hyperlinks for datasets deposited in an approved repository, but for which data deposition is not mandated (see here for details http://www.nature.com/sdata/data-policies/repositories).

• At a minimum, please include a statement confirming that all relevant data are available from the authors, and/or are included with the manuscript (e.g. as source data or supplementary information), listing which data are included (e.g. by figure panels and data types) and mentioning any restrictions on availability.

• If a dataset has a Digital Object Identifier (DOI) as its unique identifier, we strongly encourage including this in the Reference list and citing the dataset in the Methods.

We recommend that you upload the step-by-step protocols used in this manuscript to the Protocol Exchange. More details can found at www.nature.com/protocolexchange/about.

DISPLAY ITEMS – main display items are limited to 6-8 main figures and/or main tables for Articles, Resources, Technical Reports; and 5 main figures and/or main tables for Letters. For Supplementary Information see below.

FIGURES – Colour figure publication costs $600 for the first, and $300 for each subsequent colour figure. All panels of a multi-panel figure must be logically connected and arranged as they would appear in the final version. Unnecessary figures and figure panels should be avoided (e.g. data presented in small tables could be stated briefly in the text instead).

All imaging data should be accompanied by scale bars, which should be defined in the legend. Cropped images of gels/blots are acceptable, but need to be accompanied by size markers, and to retain visible background signal within the linear range (i.e. should not be saturated). The boundaries of panels with low background have to be demarked with black lines. Splicing of panels should only be considered if unavoidable, and must be clearly marked on the figure, and noted in the legend with a statement on whether the samples were obtained and processed simultaneously. Quantitative comparisons between samples on different gels/blots are discouraged; if this is unavoidable, it should only be performed for samples derived from the same experiment with gels/blots were processed in parallel, which needs to be stated in the legend.

Figures should be provided at approximately the size that they are to be printed at (single column is 86 mm, double column is 170 mm) and should not exceed an A4 page (8.5 x 11"). Reduction to the scale that will be used on the page is not necessary, but multi-panel figures should be sized so that

the whole figure can be reduced by the same amount at the smallest size at which essential details in each panel are visible. In the interest of our colour-blind readers we ask that you avoid using red and green for contrast in figures. Replacing red with magenta and green with turquoise are two possible colour-safe alternatives. Lines with widths of less than 1 point should be avoided. Sans serif typefaces, such as Helvetica (preferred) or Arial should be used. All text that forms part of a figure should be rewritable and removable.

We accept files from the following graphics packages in either PC or Macintosh format:

- For line art, graphs, charts and schematics we prefer Adobe Illustrator (.AI), Encapsulated PostScript (.EPS) or Portable Document Format (.PDF). Files should be saved or exported as such directly from the application in which they were made, to allow us to restyle them according to our journal house style.

- We accept PowerPoint (.PPT) files if they are fully editable. However, please refrain from adding PowerPoint graphical effects to objects, as this results in them outputting poor quality raster art. Text used for PowerPoint figures should be Helvetica (preferred) or Arial.

- We do not recommend using Adobe Photoshop for designing figures, but we can accept Photoshop generated (.PSD or .TIFF) files only if each element included in the figure (text, labels, pictures, graphs, arrows and scale bars) are on separate layers. All text should be editable in 'type layers' and line-art such as graphs and other simple schematics should be preserved and embedded within 'vector smart objects' - not flattened raster/bitmap graphics.

- Some programs can generate Postscript by 'printing to file' (found in the Print dialogue). If using an application not listed above, save the file in PostScript format or email our Art Editor, Allen Beattie for advice (a.beattie@nature.com).

Regardless of format, all figures must be vector graphic compatible files, not supplied in a flattened raster/bitmap graphics format, but should be fully editable, allowing us to highlight/copy/paste all text and move individual parts of the figures (i.e. arrows, lines, x and y axes, graphs, tick marks, scale bars etc.). The only parts of the figure that should be in pixel raster/bitmap format are photographic images or 3D rendered graphics/complex technical illustrations.

All placed images (i.e. a photo incorporated into a figure) should be on a separate layer and independent from any superimposed scale bars or text. Individual photographic images must be a minimum of 300+ DPI (at actual size) or kept constant from the original picture acquisition and not decreased in resolution post image acquisition. All colour artwork should be RGB format.

FIGURE LEGENDS – must not exceed 350 words for each figure to allow fit on a single printed NCB page together with the figure. They must include a brief title for the whole figure, and short descriptions of each panel with definitions of the symbols used, but without detailing methodology.

TABLES – main tables should be provided as individual Word files, together with a brief title and legend. For supplementary tables see below.

SUPPLEMENTARY INFORMATION – Supplementary information is material directly relevant to the conclusion of a paper, but which cannot be included in the printed version in order to keep the manuscript concise and accessible to the general reader. Supplementary information is an integral part of a Nature Cell Biology publication and should be prepared and presented with as much care as the main display item, but it must not include non-essential data or text, which may be removed at the editor's discretion. All supplementary material is fully peer-reviewed and published online as part of the HTML version of the manuscript. Supplementary Figures and Supplementary Notes are appended at the end of the main PDF of the published manuscript.

Supplementary items should relate to a main text figure, wherever possible, and should be mentioned sequentially in the main manuscript, designated as Supplementary Figure, Table, Video, or Note, and numbered continuously (e.g. Supplementary Figure 1, Supplementary Figure 2, Supplementary Table 1, Supplementary Table 2 etc.).

Unprocessed scans of all key data generated through electrophoretic separation techniques need to be presented in a supplementary figure that should be labelled and numbered as the final supplementary figure, and should be mentioned in every relevant figure legend. This figure does not count towards the total number of figures and is the only figure that can be displayed over multiple pages, but should be provided as a single file, in PDF or TIFF format. Data in this figure can be displayed in a relatively informal style, but size markers and the figures panels corresponding to the presented data must be indicated.

The total number of Supplementary Figures (not including the "unprocessed scans" Supplementary Figure) should not exceed the number of main display items (figures and/or tables (see our Guide to Authors and March 2012 editorial http://www.nature.com/ncb/authors/submit/index.html#suppinfo; http://www.nature.com/ncb/journal/v14/n3/index.html#ed). No restrictions apply to Supplementary Tables or Videos, but we advise authors to be selective in including supplemental data.

Each Supplementary Figure should be provided as a single page and as an individual file in one of our accepted figure formats and should be presented according to our figure guidelines (see above). Supplementary Tables should be provided as individual Excel files. Supplementary Videos should be provided as .avi or .mov files up to 50 MB in size. Supplementary Figures, Tables and Videos much be accompanied by a separate Word document including titles and legends.

GUIDELINES FOR EXPERIMENTAL AND STATISTICAL REPORTING

REPORTING REQUIREMENTS – We are trying to improve the quality of methods and statistics reporting in our papers. To that end, we are now asking authors to complete a reporting summary that collects information on experimental design and reagents. The Reporting Summary can be found here https://www.nature.com/documents/nr-reporting-summary.pdf)If you would like to reference the guidance text as you complete the template, please access these flattened versions at http://www.nature.com/authors/policies/availability.html.

STATISTICS – Wherever statistics have been derived the legend needs to provide the n number (i.e. the sample size used to derive statistics) as a precise value (not a range), and define what this value represents. Error bars need to be defined in the legends (e.g. SD, SEM) together with a measure of centre (e.g. mean, median). Box plots need to be defined in terms of minima, maxima, centre, and

percentiles. Ranges are more appropriate than standard errors for small data sets. Wherever statistical significance has been derived, precise p values need to be provided and the statistical test used needs to be stated in the legend. Statistics such as error bars must not be derived from n<3. For sample sizes of n<5 please plot the individual data points rather than providing bar graphs. Deriving statistics from technical replicate samples, rather than biological replicates is strongly discouraged. Wherever statistical significance has been derived, precise p values need to be provided and the statistical test stated in the legend.

Information on how many times each experiment was repeated independently with similar results needs to be provided in the legends and/or Methods for all experiments, and in particular wherever representative experiments are shown.

We strongly recommend the presentation of source data for graphical and statistical analyses as a separate Supplementary Table, and request that source data for all independent repeats are provided when representative experiments of multiple independent repeats, or averages of two independent experiments are presented. This supplementary table should be in Excel format, with data for different figures provided as different sheets within a single Excel file. It should be labelled and numbered as one of the supplementary tables, titled “Statistics Source Data”, and mentioned in all relevant figure legends.

--------- Please don't hesitate to contact NCB@nature.com should you have queries about any of the above requirements ---------

Author Rebuttal to Initial comments

Point-by-Point Response to Reviewers’ Comments

We thank the Reviewers and the Editor for the important and constructive critique and suggestions that have helped us to further strengthen our manuscript. As detailed below in our point-by-point response to Reviewers’ comments, we have generated a large body of new data (highlighted in grey in the text) addressing the majority of the points raised.

Point-by-point response to Referee 1:

“However, the study seems to end up a little bit suddenly after the establishment of the Pu.1 and Daxx double knockout murine system.”

This important point has been addressed below. In particular, we have generated a large body of work based on in-depth molecular annotation of chromatin and phenotypic changes in the DKO mice. This has been extremely helpful in strengthening our conclusions and provided further evidence with respect to the interaction between the two pathways.

Comment 1. As much of the study relies on the Mx1Cre line to achieve inducible Daxx knockout (including physiological characterization and the following molecular experiments), is there any reason for the group to initially use RosaCreERT2+/- line for the characterization of chromatin landscape upon loss of Daxx?

Reply: We have started using this mouse line since our initial analysis aimed at investigating molecular changes in HSCs at an early time point following Daxx loss (i.e. shortly after end of induction) and wanted to avoid the use of treatments promoting IFN response, i.e. pI:pC in the case of Mx1Cre line. In this respect, in response to comments raised by Referee 2 we have now run transcriptomics on LT-HSCs in RosaCreERT2 mice that have been integrated with chromatin landscape data (**Figure 1**). Therefore we now have similar sets of genomics data for both ROSACreERT2 (inducible, ubiquitous) and Mx1Cre lines (inducible, hematopoiesisspecific). Furthermore, we now show that phenotypically the two models display similar changes in hematopoiesis (**Figure 2**).

Comment 2. As Pu.1 knockout partially rescues the phenotypes seen in Daxx knockout mice, has the group done any investigation to check if the altered chromatin landscape due to Daxx knockout can be restored to some extent by Pu.1 knockout?"

Reply: We have now performed ATAC-seq on *Daxx*$^{F/F}$, *Daxx*$^{WT/WT}$ and *Daxx*$^{F/F}$;*Pu.1*$^{F/F}$ Mx1Cre KLS cells. These new data show that changes in chromatin accessibility in Daxx KO KLS cells are substantially reverted upon concomitant Daxx and Pu.1 deletion (over 90% reversion, **Figure 8g,h; Figure 1 for Referee**). Interestingly, these changes also correlated with decreased transcription of neighboring genes in DKO cells (**Figure 8i**). Furthermore, as highlighted below Daxx loss markedly changed Pu.1 chromatin association genome-wide, further suggesting an interaction between the two pathways in regulation of hematopoiesis (**Figure 6a,c**). This is also supported by the observation that a large subset of transcriptional changes in DKO KLS cells (vs WT or single KOs) are unique and not found in Pu.1 KO and Daxx KO cells (**Figure 8a-d, Ext. Data Fig. 8a-g**), supporting an interaction **between the two pathways.**

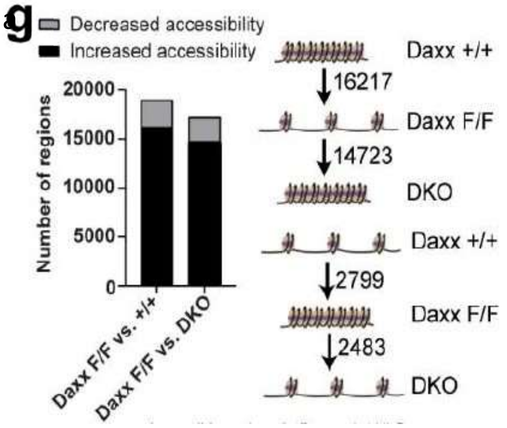

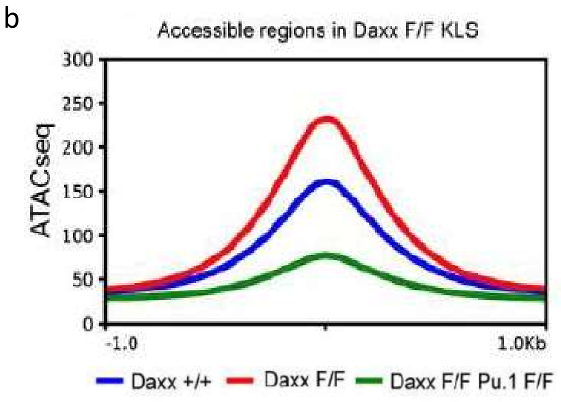

Figure 1 for Reviewer. Concomitant loss of Daxx and Pu.1 reverts most of the chromatin accessibility changes observed in Daxx-deficient animals (**a,b**). Panels can be found in **Figure 8** in our manuscript.

Comment 3. Though the group has pointed out the altered expression profile of some ERVs, such as LTRs and TERRA lncRNA, in the Daxx knockout KLS cells, does the group have any evidence to suggest the functional significance of such altered ERV expression profile?

Reply: We thank the reviewer for raising this point. One could hypothesize that ERV-mediated activation of the RNA-sensing machinery and resulting IFN type-I production and IFNR signaling could contribute to changes in hematopoietic differentiation observed in Daxx-deficient animals. Interestingly, inactivation of the Setdb1/Kap1 complex, a known Daxx interactor results in deregulation of ERVs and inhibition of B-cell differentiation[1]. Furthermore, recent work in Zebrafish implicates RTE expression and engagement of RNA-sensing receptors RIG-I and Mda5 in emergence of HSPCs during development, further suggesting a role for RTE-dependent signaling in hematopoiesis[2]. An important question is whether changes in RNA-sensing and/or IFN type-I signaling could be detected shortly after Daxx deletion, as this could provide some (circumstantial) evidence for a more direct role in regulation of hematopoietic differentiation. Our new data show that in LT-HSCs (on a ROSA background) acute loss of Daxx indeed results in clear upregulation of components of the IFN type-I response (IFN-stimulated genes, ISGs), such as *Irf7*, *Mx1* and the *Ifit* cluster (**Figure 1g,i,k** and **o,p; Figure 2 for Reviewer**), correlating with upregulation of selected ERVs (**Figure 1j**). IFN type-I signaling is normally repressed in HSCs, but when activated it promotes cell cycle entry of quiescent HSCs[3]. Indeed, upon acute Daxx loss we found an increased number of LT-HSCs, along with other progenitor types correlating with augmented production of neutrophils, in agreement with augmented ISG expression (**Figure 2a-g**). Finally, since it is presently unclear if stem and progenitor cells are able to produce IFNs, ISG induction could also be IFN-independent as reported in viral infection models[4-6]. Although IFNs could still be produced by Daxx-deficient mature cells, proliferating HSCs are known to undergo cell death upon IFN type-I receptor stimulation[7], something that would be difficult to reconcile with LT-HSC expansion upon acute Daxx loss.

It is likely that after a first wave of proliferation and differentiation, Daxx-deficient HSCs may return to a quiescence state, but chronic Daxx loss may still skew hematopoiesis towards myeloid differentiation. This resembles what is observed upon viral infections, where early HSC activation is followed by return to quiescence and an increased percentage of myeloid-biased HSCs[8], as it is also observed in aging[9].

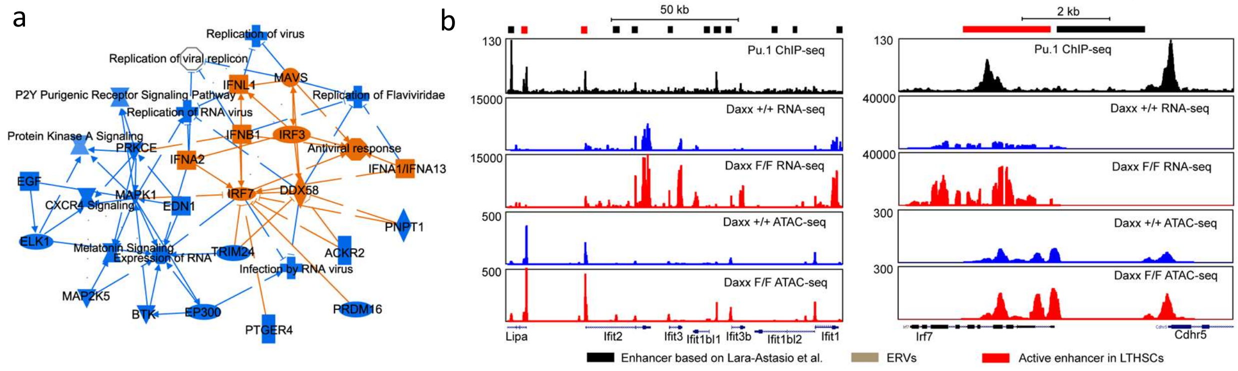

Figure 2 for Reviewer. Acute loss of Daxx in LT-HSCs promotes a IFN type-Iike response (**a**) with key ng Panels can be found in **Figure 1** in our manuscript. upregulates (**b**).

<u>Comment 4</u>. Has the group ever considered to check any histone epigenetic marks (e.g. H3K27me3) in both the Daxx knockout and Daxx/Pu.1 double knockout mice?"

Reply: We agree that this is another important point that will allow us to shed light on potential mechanisms contributing to chromatin changes and transcriptional alterations. In addition to H3K27me3, we decided to mostly focus on marks that have been linked to Daxx, such as H3K9me3 in regulation of ERV silencing[10, 11]. Given that ERVs can function as enhancers, we included H3K27Ac as active enhancer mark. Furthermore, due to the role of Pu.1 in mediating the response to Daxx loss and the function of Daxx as H3.3 chaperone, we added Pu.1 and H3.3 to our Cut&Tag pipeline, respectively. In order to obtain sufficient number of cells, we chose HSPCs as most suitable progenitor cells. Finally, these data were integrated with newly generated ATAC-seq data. These experiments are covered also below in our response to Referee 3 (see also **Summary Table** below for all genomics data generated during the course of this study; data produced during the revision in grey).

Mouse line	Genotype	Celltype	Time after last treatment	Number of mice	Genomics Assay	Antibody targets
ROSA-CRE-ERT2	Daxx +/+	LTHSC	3 days	2	RNA-seq	NA
ROSA-CRE-ERT2	Daxx +/+	LTHSC	3 days	2	ATAC-seq	NA
ROSA-CRE-ERT2	Daxx F/+	LTHSC	3 days	2	RNA-seq	NA
ROSA-CRE-ERT2	Daxx F/F	LTHSC	3 days	3	RNA-seq	NA
ROSA-CRE-ERT2	Daxx F/F	LTHSC	3 days	3	ATAC-seq	NA
ROSA-CRE-ERT2	Daxx +/+	LTHSC	3 wpi	2	ATAC-seq	NA
ROSA-CRE-ERT2	Daxx +/+	CMP	3 wpi	2	ATAC-seq	NA
ROSA-CRE-ERT2	Daxx +/+	GMP	3 wpi	2	ATAC-seq	NA
ROSA-CRE-ERT2	Daxx F/F	LTHSC	3 wpi	2	ATAC-seq	NA
ROSA-CRE-ERT2	Daxx F/F	CMP	3 wpi	2	ATAC-seq	NA
ROSA-CRE-ERT2	Daxx F/F	GMP	3 wpi	2	ATAC-seq	NA
Mx1-CRE	Daxx +/+	KLS	3 wpi	2	RNA-seq	NA
Mx1-CRE	Daxx +/+	KLS	3 wpi	2	ATAC-seq	NA
Mx1-CRE	Daxx F/F	KLS	3 wpi	2	RNA-seq	NA
Mx1-CRE	Daxx F/F	KLS	3 wpi	2	ATAC-seq	NA
Mx1-CRE	Daxx F/F Pu.1 F/F	KLS	3 wpi	2	RNA-seq	NA
Mx1-CRE	Daxx F/F Pu.1 F/F	KLS	3 wpi	2	ATAC-seq	NA
Mx1-CRE	Pu.1 F/F	KLS	3 wpi	2	RNA-seq	NA
Mx1-CRE	Daxx +/+	GMP	3 wpi	2	RNA-seq	NA
Mx1-CRE	Daxx F/F	GMP	3 wpi	2	RNA-seq	NA
Mx1-CRE	Daxx F/F Pu.1 F/F	GMP	3 wpi	2	RNA-seq	NA
Mx1-CRE	Pu.1 F/F	GMP	3 wpi	2	RNA-seq	NA
Mx1-CRE	Daxx +/+	MPP3	3 wpi	2	RNA-seq	NA
Mx1-CRE	Daxx F/F	MPP3	3 wpi	2	RNA-seq	NA
Mx1-CRE	Daxx +/+	MPP4	3 wpi	2	RNA-seq	NA
Mx1-CRE	Daxx F/F	MPP4	3 wpi	2	RNA-seq	NA
Mx1-CRE	Daxx +/+	KLS	24 wpi	2	RNA-seq	NA
Mx1-CRE	Daxx F/F	KLS	24 wpi	2	RNA-seq	NA
Mx1-CRE	Daxx F/F Pu.1 F/F	KLS	24 wpi	2	RNA-seq	NA
Mx1-CRE	Daxx +/+	GMP	24 wpi	2	RNA-seq	NA
Mx1-CRE	Daxx F/F	GMP	24 wpi	2	RNA-seq	NA
Mx1-CRE	Daxx F/F Pu.1 F/F	GMP	24 wpi	2	RNA-seq	NA
Mx1-CRE	Daxx +/+	KLS	Transplantation	2	RNA-seq	NA
Mx1-CRE	Daxx F/F	KLS	Transplantation	2	RNA-seq	NA
Mx1-CRE	Daxx +/+	GMP	Transplantation	2	RNA-seq	NA
Mx1-CRE	Daxx F/F	GMP	Transplantation	2	RNA-seq	NA
Mx1-CRE	Daxx +/+	HSPC	3 wpi	2	CUT&Tag	H3.3, Pu.1, H3K9me3, H3K27ac
Mx1-CRE	Daxx F/F	HSPC	3 wpi	2	CUT&Tag	H3.3, Pu.1, H3K9me3, H3K27ac
Mx1-CRE	Daxx +/+	HSPC	8 wpi	3	CUT&Tag	H3K9me3, H3K27me3
Mx1-CRE	Daxx F/F	HSPC	8 wpi	3	CUT&Tag	H3K9me3, H3K27me3
Mx1-CRE	Daxx F/F Pu.1 F/F	HSPC	8 wpi	3	CUT&Tag	H3K9me3, H3K27me3
Mx1-CRE	Daxx +/+	HSPC	24 wpi	2	CUT&Tag	H3.3, Pu.1, H3K9me3, H3K27ac, H3K27me3
Mx1-CRE	Daxx F/F	HSPC	24 wpi	2	CUT&Tag	H3.3, Pu.1, H3K9me3, H3K27ac, H3K27me3
Mx1-CRE	Daxx F/F Pu.1 F/F	HSPC	24 wpi	2	CUT&Tag	H3.3, H3K9me3, H3K27ac, H3K27me3
New datasets						

We have run H3K9me3 CUT&Tag in wildtype, single Daxx KO and Daxx/Pu.1 double knockout mice. As seen in **Figure 8j**, double KO mice display reversal of changes in H3K9me3 seen in single Daxx KO cells (depicted there are distal regions). In addition, we also ran H3.3 CUT&Tag for all three genotypes (at 24 wpi). Interestingly, H3.3 depletion at enhancers and ERVs in Daxx single KO cells is also reversed in Daxx/Pu.1 double KO mice, suggesting that other H3.3 chaperones, such as Hira can be engaged upon loss of Pu.1 and Daxx. Generally, H3.3 deposition is in general increased in double KO cells (**see Figure 8k**).

We would like to bring the *Fbp1* and *Fbp2* locus as example of changes at chromatin level in Daxx KO and DKO progenitors (**see Figure 8l**). This locus encodes for critical mediators of gluconeogenesis that have been suggested to compromise HSC/HSPC repopulation capacity when de-repressed upon loss of Setdb1-mediated H3K9 methylation[12].

Figure 3 for Reviewer. Genome browser coverage plot of the *Fbp1* and *Fbp2* locus.
Figure 8 in the manuscript.

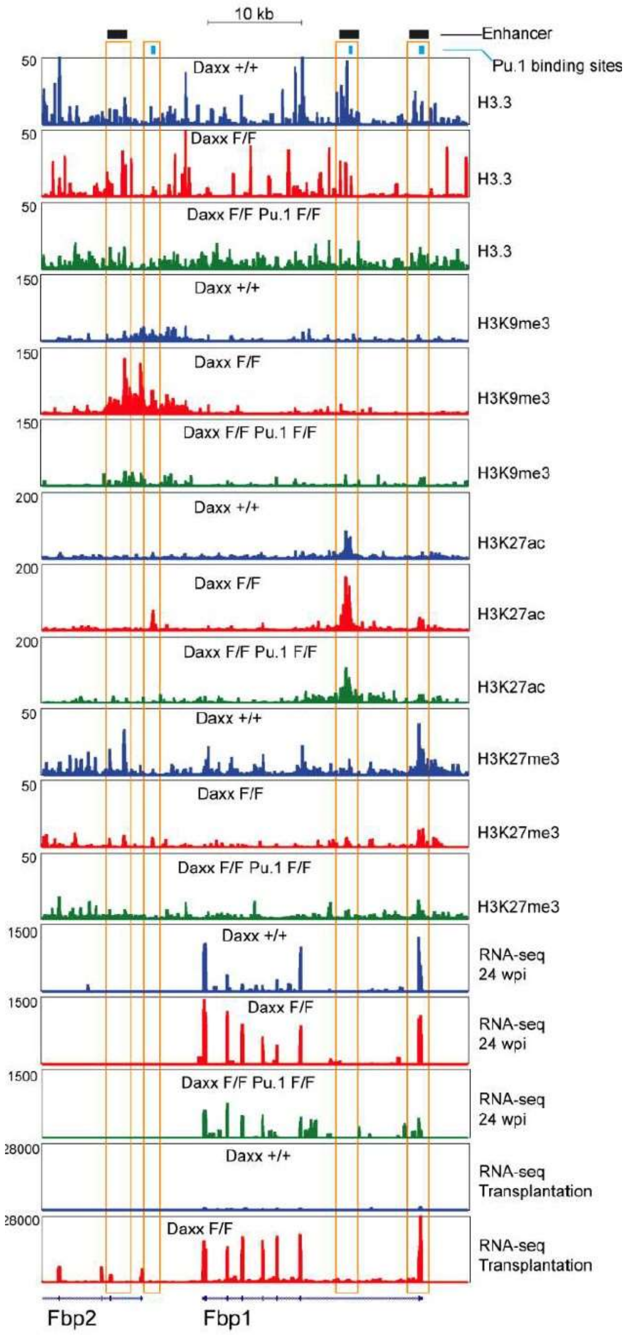

Our main findings are (coverage plots s shown in **Figure 3 for Reviewer**):

i) *Fbp1* is upregulated upon Daxx loss in KLS at 24wpi, while *Fbp2* remains silenced. *Fbp1* upregulation is even more marked in transplantation conditions. In DKO cells, *Fbp1* expression is restored to WT levels, suggesting that its upregulation relies on Pu.1.

ii) There are three main regions that are bound by Pu.1 in KLS cells at this locus, two of which are known enhancers. Upon Daxx loss, there is increase in H3K27Ac at all of these regions, a phenomenon that is reverted in DKO cells. While H3.3 is present at these regulatory regions, Daxx loss mostly abrogates H3.3 levels at the enhancer/Pu.1-binding site most proximal to *Fbp1*. Interestingly, H3.3 is restored at this region in DKO cells, suggesting that another H3.3 chaperone, like Hira may be recruited upon Pu.1 loss.

iii) The enhancer most proximal to *Fbp1* display limited H3K9me3 enrichment in WT cells, which is abrogated in Daxx KO cells and restored in DKO cells. Notably, there is a large block of H3K9 trimethylation over the *Fbp2* gene and its regulatory regions, which was previously reported to be abrogated in Setdb1-deficient cells and linked to *Fbp2* upregulation in HSPCs[12]. Interestingly, H3K9me3 is further enriched at this region in Daxx-deficient cells (but not in DKO cells), suggesting that the reported interaction between Daxx and the Setdb1/Kap1 complex[11] does mediate H3K9 trimethylation at this locus and it is even possible that Daxx inhibits it. In agreement with these findings, Daxx loss results in *Fbp1* upregulation only in conditions of steady state hematopoiesis (**Fig. 8l**). In contrast, Setdb1 loss mostly affects *Fbp2* expression[12]. In transplantation conditions, both *Fbp1* and *Fbp2* are completely silenced in WT cells, whilst in KO cells we observed some *Fbp2*

expression along with clear *Fbp1* upregulation, suggesting that both loci are engaged by Daxx loss during stress hematopoiesis.

iv) Finally, H3K27me3 is substantially reduced across the entire locus in Daxx KO and DKO KLS cells, suggesting that this epigenetic mark (not H3K9me3) could be involved in repression of the enhancer at the 5' of *Fbp1*.

<u>Point-by-point response to Referee 2:</u>

"Although the study is well written, it is not clear how Daxx deletion specifically impacts stem cells and to what extent does the Daxx deletion in HSCs contribute to neutrophilia, systemic inflammation and B cell loss."

Reply: This general comment is covered below as part to the individual response to specific points raised by the Reviewer.

<u>Comment 1</u>. Undoubtedly, Daxx plays an important role during hematopoiesis, however the data presented in this study doesn't support a role of Daxx in HSC function. In Figure 1, the authors present ATAC-seq data of chromatin architecture performed on HSCs CMPs and GMPs. Arguing that Daxx deleted HSCs chromatin landscape resembled that of myeloid progenitors, which contributes to myeloproliferation and suppression of lymphopoiesis. However, it is unclear whether Daxx deletion in HSCs is driving the described phenotypes presented for DaxxF/F/ Mx-1 Cre mice (myeloproliferation, neutrophil expansion and B cells loss).
In Extended Data Figure 1a, the authors describe that Daxx is highly expressed in HSCs, MEPs and B cells. However, the expression levels in other populations is similar. Thus, deletion of Daxx in LMPPs, CLPs and B220+ cells may contribute to poor B cell maintenance and survival, especially because in Extended Data Fig.2f authors report no statistically different levels of lymphoid progenitors (LMPP) upon Daxx deletion suggesting HSCs can give rise to lymphoid-prone progenitors.
As Mx1-Cre exhibits nonselective recombination in hematopoietic cells, the authors need to clarify whether the phenotypes attributed to DaxxF/F/ Mx1-Cre model is due to stem cell dysfunction or due to failure of more committed progenitors or terminally differentiated cells.

Reply: We thank the Reviewer for recognizing the important role of the H3.3 chaperone Daxx in hematopoiesis. We agree that in the earlier version of our manuscript it was unclear whether Daxx loss is or is not directly related to stem cell perturbations. We also agree the best way to address this point would be to run *in-vivo* experiments (e.g. study of HSC quiescence using serial transplantation and so on, as covered by another point) aimed at addressing the functionality of stem and progenitor cells upon loss of Daxx. Unfortunately, the current and past limitations of operations of our facilities (in particular the animal facility) in occasion of the spring and autumn/winter coronavirus surges have not allowed us to perform transplantation experiments, as they implied long-term follow-up that was not possible. Nonetheless, we produced a large body of work that we believe has strengthened the conclusions of our work.

Firstly, we considered that most of our previous data was based on analysis of transcriptome and biological phenotypes in the context of chronic Daxx deletion. Therefore, we reasoned that it would be quite insightful if acute Daxx loss was sufficient to i) alter the transcriptome and chromatin landscape of

LT-HSCs shortly after induction and ii) to promote early changes in hematopoietic differentiation that resembled those observed upon chronic Daxx loss (Mx1-Cre model).

These are our main findings:

i) **Acute Daxx loss affects both chromatin landscape and transcriptome in LT-HSCs and promotes induction of genes which are part of the IFN type-I response (Figure 1a,b for Reviewer).** We generated LT-HSC transcriptome data on a RosaCreER background, in order to monitor acute effects of Daxx loss without the potentially confounding effect of acute pI:pC response (as in Mx1Cre mice). With respect to RosaCreERT2 mice, we have now used a more effective tamoxifen route (i.p. instead of oral gavage) for Daxx deletion, which results in phenotypic changes very similar to those observed in the Mx1 model (see **Figure 2**, also addressing a point raised by the same Reviewer below). Interestingly, acute loss of Daxx indeed results in clear upregulation of components of the IFN type-I response (IFN-stimulated genes, ISGs), such as *Irf7*, *Mx1* and the *Ifit* cluster (**Figure 1g,i,k and o,p**), correlating with upregulation of selected RTEs (**Figure 1j**). Interestingly, recent work in Zebrafish implicates RTE expression and engagement of RNA-sensing receptors RIG-I and Mda5 in emergence of HSPCs during development, suggesting a role for RTE-dependent signaling in hematopoiesis[2]. Furthermore, expression of *Mki67* was enhanced along with suppression of *Egr1*, a key inhibitor of HSC quiescence and mobilization[13]. Hence, acute Daxx loss causes not only changes to chromatin landscapes in LT-HSCs but also alters their transcriptome, potentially reflecting stress response, activation and/or entry into cell cycle. IFN type-I signaling is normally repressed in HSCs, but when activated it promotes cell cycle entry of quiescent HSCs[3]. Indeed, upon acute Daxx loss we found an increased number of LT-HSCs, along with other progenitor types, correlating with augmented production of neutrophils, in agreement with augmented ISG expression (**Figure 2a-g; see also bullet point below**). Since it is presently unclear if stem and progenitor cells are able to produce IFNs, ISG induction could also be IFN-independent as reported in viral infection models[4-6]. Although IFNs could still be produced by Daxx-deficient mature hematopoietic cells, proliferating HSCs are known to undergo cell death upon IFN type-I receptor stimulation[7], a finding that would be difficult to reconcile with LT-HSC expansion upon acute Daxx loss. Finally, it is likely that after a first wave of proliferation and differentiation, Daxx-deficient HSCs may return to a quiescence state, but chronic Daxx loss may still skew hematopoiesis towards myeloid differentiation. This resembles what is observed upon viral infections, where early HSC activation is followed by return to quiescence and an increased percentage of myeloid-biased HSCs[8], as it is also observed in aging[9].

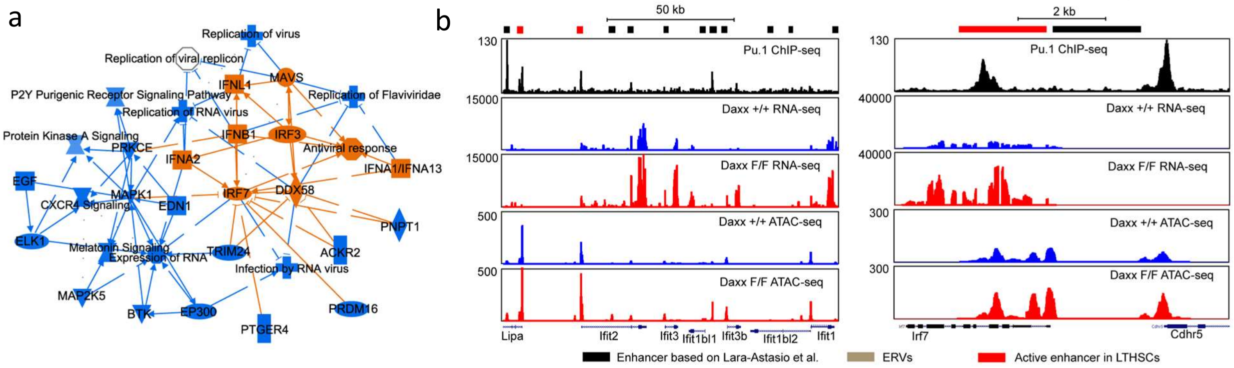

Figure 1 for Reviewer. Acute loss (3dpi) of Daxx in LT-HSCs promotes a IFN type-like response (**a,b**). Key ISGs such as the *Ifit* cluster and *Irf7* displayed chromatin opening at known enhancers and increased expression. Panels can be found in **Figure 1** in the manuscript.

ii) **Chromatin and transcriptional changes caused by acute Daxx loss correlate with early expansion of stem and progenitor cells and unbalanced differentiation (Figure 2 for Reviewer).** At 3dpi, we show a significant increase in both frequency and number of BM LTHSCs, KLS and MPP3 and GMPs (see **Figure 2a,b**) and significantly increased MPP4 and GMP frequency. Furthermore, IgM/B220 staining revealed a reduction in B-cell differentiation in the BM (**Figure 2d**). These early changes also correlated with a drop in overall B cell number and frequency in the BM, and increased frequency of neutrophils (**Figure 2c**). In the spleen, number and frequency of B cells were not affected (a small increase in B-cell death was detected but it was not significant, **Figure 2e,f**). In contrast, the number of neutrophils was significantly up and number of monocytes down. At 2wpi, the drop in B cell frequency and number becomes clear also in both BM (not shown) and spleen (**Figure 2g**).

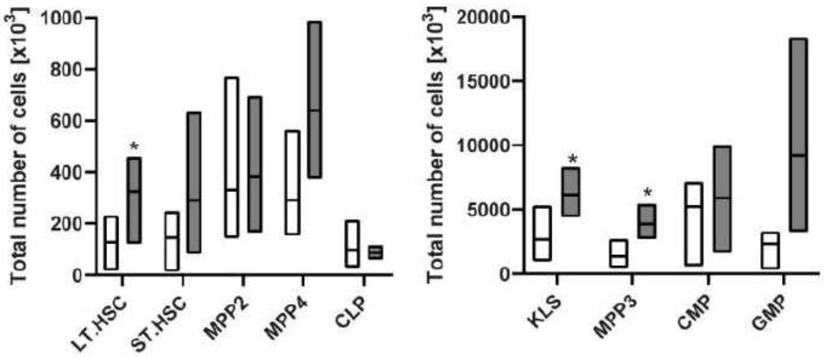

Figure 2 for Reviewer. Altered molecular identity of MPP4 cells in Daxx-deficient mice. MPP4 display higher myeloid gene expression (**a**) and reduced lymphoid gene expression (**b**) and cluster closer to WT MPP3 than WT MPP4 (**a,b**). Panels can be found in **Figure 5** in the manuscript.

iii) **Daxx KO MPPs display transcriptional alterations compatible with altered molecular identity.** Once it became clear that Daxx loss does not impinge on production of multipotent progenitors, we decided to investigate whether Daxx-deficient multipotent progenitors display transcriptional perturbations compatible with altered cell identity. We found that MPP3 displayed

higher expression of myeloid transcription factors. While Daxx KO MPP4 also showed increased expression of myeloid transcription factors, albeit less pronounced than KO MPP3, they displayed lower expression of lymphoid transcription factors, suggesting a potential myeloid potential of MPP4 cells in Daxx KO animals (**see Figure 5j**). Accordingly, the myeloid transcription factor heatmap shows clustering of WT MPP3 with KO MPP4 (**see Figure 5j; Figure 3 for Reviewer below**).

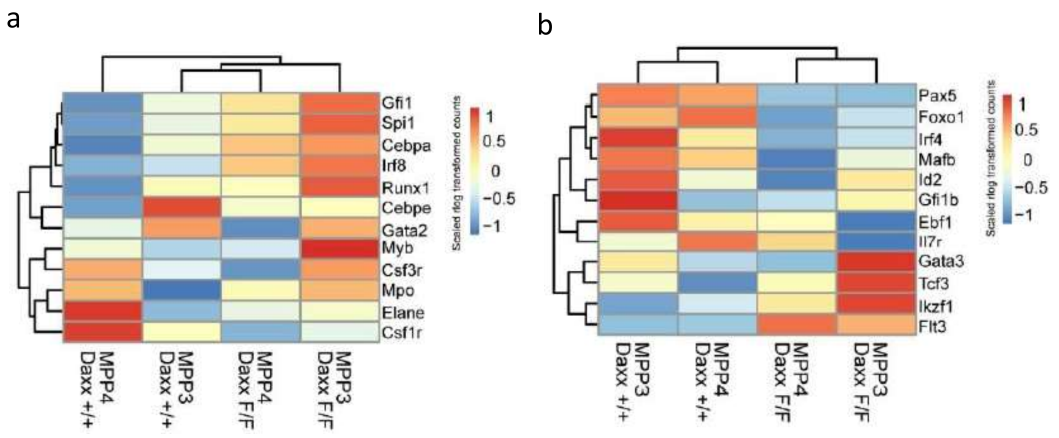

Figure 3 for Reviewer. MPP3 and MPP4 transcriptional changes upon Daxx loss suggest altered cell identity/fate.

Overall, the demonstration of early molecular perturbations of LT-HSCs co-occurring with changes in hematopoietic differentiation suggest that Daxx-mediated regulation of stem chromatin has implications for hematopoiesis. Molecular changes in multipotent progenitors can be the result of alterations at stem cell level and/or indicate a potential role of Daxx also in these cells. Further investigation aimed at determining the differentiation potential of stem cells and multipotent progenitors *in-vivo* would be important, but it is beyond our capabilities at the present time due to contingent situation.

<u>Comment 2a</u>. To prove HSC dysfunction following Daxx deletion, and differentiate the role of Daxx between HSCs and progenitors, the authors should show a more thorough analysis of HSCs and HSC function, such as analysis of quiescence and cell cycle status, self-renewal and repopulation potential following transplantation (HSC transplantation and see comment 8).

Reply: We have in part covered this point above. Despite the abovementioned considerations and logistic difficulties, we attempted transplantation of LT-HSCs, but unfortunately our first round of injections (attempted right at the time of submission) had to be suspended due to entry into lockdown

and research facilities minimum operating mode. Afterwards, due to unpredictable changes to operating mode of the animal facility, we did not manage to run these experiments despite all our efforts. It was very unfortunate, but we had no way to overcome this problem. It is still unclear when the current restrictions will be eased. For the moment, we cannot run any transplantation. Despite these shortcomings, our revised manuscript contains further insights into the potential role of Daxx in regulation of hematopoietic stem and progenitor function (as also highlighted above).

Comment 2b. To attribute the phenotypes reported following Daxx deletion to HSC dysfunction, the authors should validate their ATAC-seq and RNA-seq data in HSCs, by RT-PCR. Is the expression of myeloid and lymphoid lineage genes changed in HSCs?

Reply: We have run transcriptomics and ATAC-seq of HSCs, as detailed above.

Comment 3. The skin infection in the DaxxF/F/ Mx-1 Cre model raises the possibility that the abnormalities (myeloid shift in BM, splenomegaly, leukocytosis, changes in BM cytokines etc..) may be secondary to a deficit in leukocyte trafficking. The authors should evaluate the recruitment of leukocytes to a site of infection and they should also evaluate HSPC homing to the bone marrow to rule out an intrinsic adhesion/migration defect of leukocytes/HSPCs.

Reply: We respectfully point out that the skin disease we observed is not an infection as confirmed by a dermatology expert at Uni-Bonn (Joerg Wenzel, coauthor in the manuscript), but it closely recapitulates a human auto-inflammatory disease that is associated with marked neutrophilia. Hence, our working hypothesis is indeed that peripheral accumulation of neutrophils is what causes the observed skin phenotype. Indeed, a number of inflammatory cytokines that are upregulated in Daxx-deficient mice are normalized upon concomitant deletion of Daxx and Pu.1, which also reduces neutrophilia.

With respect to leukocytes trafficking and homeostasis, we agree that this would be an exciting hypothesis. In this respect, it is worth highlighting that we found neutrophils in Daxx KO BM, spleen and peripheral blood to present reduced expression of Cxcr2 (**Figure 2p, 3f; Ext. Data Fig. 4b**), which is a differentiation marker but also a key regulator of neutrophil homeostasis. Interestingly, it has been implicated in driving diurnal changes in the migratory properties of neutrophils, referred to as neutrophil aging[14]. Loss of the clock gene *Arntl* reduces Cxcr2 levels and inhibits neutrophil aging, in turn causing neutrophils to acquire nighttime/"fresh" features, increasing migration into tissues in homeostatic conditions while protecting from vascular damage. In contrast, loss of Cxcr4 leads to constitutive Cxcr2-dependent aging (daytime feature) and opposite effects on migration, clearance and vascular damage. It is worth noting that *Arntl* expression is significantly reduced in Daxx KO KLS cells whilst Cxcr4 is up; another clock gene, *Per2* is down in Daxx KO RosaCreER LT-HSCs. It is also known that reduced Cxcr2 causes a feedback loop from the periphery back to the BM via production of IL-23, IL-17 and G-CSF for induction of granulopoiesis[15]. Indeed, both IL-17 and IL-23 are upregulated in Daxx KO mice, whilst concomitant deletion of Daxx and Pu.1 results in IL-23 normalization along with

inhibition of neutrophilia (a trend for IL-17; **Figure 7k; Figure 4 for Reviewer**). Our findings along with the abovementioned literature prompt future investigation aimed at assessing the role of Daxx in circadian regulation of neutrophil homeostasis and aging, their clearance/migration into tissues and the implications for vascular health.

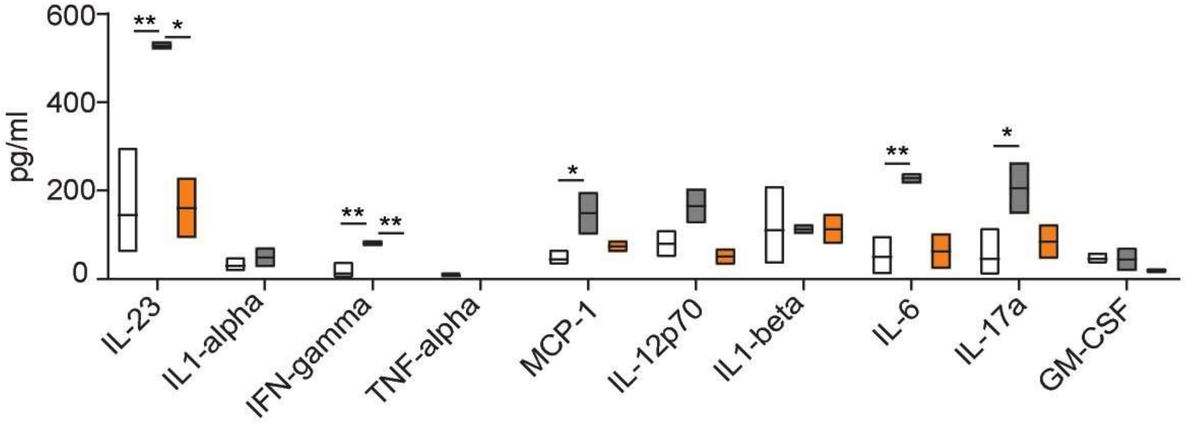

Figure 4 for Reviewer. IL-23 and IL-17a are increased in Daxx KO plasma, while their levels are reduced in DKO mice (significant for IL-23). Panels can be found in **Figure 7** in the manuscript

<u>Comment 4</u>. Throughout the manuscript, the authors report frequencies of different populations of blood cells including stem and progenitors. Since the bone marrow WBC cellularity is increased upon Daxx deletion (Extended Data Fig. 2d), the authors should report absolute numbers per femur. This is especially critical when evaluating stem and progenitor populations. For example, in Extended Data Fig2f, HSC frequency doesn't seem to change. However, ST-HSCs and LMPPs trend toward expansion, and given the increased WBC cellularity, absolute numbers of these populations may reach significance.

Reply: We have now extensively investigated changes in cell numbers (also discussed above). Overall, these data show that the variations in frequency in many cases reflect changes in overall cell numbers. This has been covered in part above with respect to RosaCreERT2 mice (see response to point 1). We now show that also in Mx1-Cre mice, numbers of several progenitor and mature cell subtypes are altered. For instance, GMPs are increased both frequency- and number-wise (**Figure 2l**) in the BM, as shown for neutrophils (**Figure 2n,o**). Interestingly, LT-HSC and ST-HSC numbers were down, along with KLS cells, suggesting that chronic Daxx deletion could lead to reduction in stem/progenitor subpopulations (**Ext Data Fig. 2i**). However, we decided against running acute Daxx loss experiments in Mx1-Cre mice, as we reasoned that pI:pC treatment would be confounding shortly after induction, given that Daxx loss itself would also promote IFN signaling (as shown in Figure 1).

Comment 5. Flow cytometry plots presented in Extended Data Fig2f, suggest that LSK population is expanded upon Daxx deletion. Are there statistically different changes in LSK cells? Because the authors don't report statistically different changes in HSCs, are any MPP population numbers changed? Given the strong myelopoiesis, one would expect changes in myeloid-biased MPPs (MPP2/MPP3).

Reply: This important point has been covered above using acute deletion of Daxx in

RosaCreERT2 mice (**Figure 2**). Briefly, we indeed see increased frequency and numbers of MPP3 cells, while MPP4 cells are only increased frequency-wise. Interestingly, Daxx-deficient mice display altered transcriptional profiles of both MPP3 and MPP4 cells, as discussed above (**Figure 5j**).

Comment 6. The authors should justify the differences between DaxxF/F/ Mx1-Cre model and DaxxF/F / RosaCreERT2 model (Extended Data Fig.4). Does the mild phenotype in the RosaCreERT2 model stem from different recombination efficiencies in HSCs, progenitors and differentiated cells? Do elevated endogenous IFN-gamma levels (trend toward increase, Fig. 4e) affect the Mx-1 Cre model? This is important as ATAC-seq experiment presented in Fig. 1 was done using the RosaCreERT2 model and serves as basis for consequent studies, and the hematopoietic phenotype in these mice is mild, especially in regards to myeloid cell expansion. Is it possible that HSCs and progenitors would show different chromatin architecture, when Daxx is deleted using Mx1-driven Cre?

Reply: As mentioned above, we have now repeated experiments in the RosaCreERT2 mice using IP injections instead of oral gavage and we see very efficient recombination and more marked myeloid expansion phenotype that arises upon acute Daxx deletion and closely resemble the one arising in Mx1Cre mice (discussed above, **Figure 1,2**). Hence, we are inclined to exclude that pI:pC treatment substantially contributes to the phenotypes arising in *Daxx*$^{F/F}$;Mx1-Cre mice.

Nonetheless, we have followed the reviewer's suggestion and performed ATAC-seq in Mx1Cre KLS cells. These new data suggest that in both models, Daxx loss causes opening at distal regions of the genome, including hematopoietic enhancers with some overlapping with ERVs and Pu.1 motifs (**Figure 5m,n**). Many more regions opened up in Mx1 KLS cells compared to Rosa LT-HSCs, but this is not surprising since KLS are a much more heterogeneous group of cells. Nonetheless, over 500 distal peaks (not shown) were common in between Daxx-deficient Mx1 KLS and Rosa LTHSCs. Overall, these data suggest that Daxx loss causes similar molecular and phenotypic changes during hematopoiesis in the two respective models.

Finally, we repeated quantification of inflammatory cytokines at 3wpi and 8wpi (**Figure 7k,l; see also above, Figure 4 for Reviewer**) IFN-gamma is significantly up at 3wpi but then it is not at 8wpi (the latter confirming the lack of significance at this time point). However, we would like to point out that pI:pC engages an IFN-type I response, implying that the transient increase in IFNgamma is unlikely the

result of pI:pC treatment. Conversely, IFN-beta was upregulated at 8wpi and unchanged at 3wpi, suggesting that this increase is not related to early pI:pC effects on IFN type-I (**Figure 7k,l**). On balance, based on the cytokine analysis and the similarities between the differentiation phenotypes arising in RosaCreERT2 and Mx1Cre models, we do not believe pI:pC treatment plays a substantial role in our experimental settings.

Comment 7. The authors don't provide quantification for imaging data presented throughout the study. For example, in Fig. 3e, the authors describe that histology and immunohistochemistry analysis of the spleen revealed expansion of CD11b+ cells while F4/80+ cells did not change. The authors must quantify their immunohistochemistry data. Representative images showing focused areas of the tissue (such as in Fig. 3e, 4f), rather than showing the entire tissue (or large area of tissue) makes it hard to interpret changes in cell density or amounts.

Reply: We indeed agreed that quantification of changes at tissue levels is important. We have now generated a whole new set of data that show indeed that changes in CD11b and F4/80 positivity are statistically significant upon Daxx loss in the spleen. We have also included images at both low and high magnification, as requested by the reviewer (**Figure 3i,j**). Data in **Figure 4f,g** suggest the autoinflammatory origin of these lesions, i.e. characterized by high myeloid cell infiltration and the presence of neutrophil extracellular traps, key markers of human *Pyoderma gangrenosum* (PG). Hence, we do not think quantification of these two markers in comparison to normal skin would be meaningful, as the disease was already categorized as closely resembling human PG by an expert of these autoinflammatory skin conditions.

Comment 8. It is difficult to interpret the competitive bone marrow transplantation studies presented in Extended Data Fig. 8. It is not clear what is the time line of the experiments. The authors indicate that peripheral blood data was collected at two different time points given as a range: 4-5 weeks and 8-12 weeks. For terminal data collected (reconstitution of BM and spleen) the authors indicate the data was collected 12-17 weeks post transplantation (Extended Data Fig.8e,f,g). How was the data collected? Were data points pooled from bleeding and sacrificing mice at different time points in the range indicated? This may explain the high variability and lack of significance for donor derived LSK cells and GMPs. The authors should represent peripheral blood data in a clear time course (4, 8, 12 and 16 weeks) to make a clear distinction between short term reconstitution and long-term reconstitution (attributed to HSC function). Terminal data collection for bone marrow and spleen analysis should be done at one time point, preferably 16 weeks.

Reply: We agree that the experiment shown in previous Ext. Data Fig 8 was not optimal. Unfortunately, due to restrictions in our animal facility and other core infrastructure, transplants had not been feasible as discussed above.

<u>Comment 9</u>. In Extended Fig 9b, the authors show lower expression of lymphoidassociated genes and higher expression of myeloid-associated gene (Extended Fig 9b) in DaxxF/F / Mx-1 Cre LSK cells (RNAseq analysis), concluding that lymphoid-committed LSK cells should be reduced upon Dax deletion. However, In Extended Fig 2f, the authors show no significant change in LMPPs (also MPP4), which have been described in the literature as lymphoid-prone multi potent progenitors. What's more, the data point to a trend toward expansion of LMPP frequency. Based on their data the authors can't make this conclusion.

Reply: This is an important point, which we have in part covered above. We agree with the reviewer that the transcriptional changes observed in our previous RNA-seq data run on KLS cells in transplantation conditions are unlikely due to changes in progenitor subpopulations, i.e. loss of lymphoid-committed progenitor cells. It is instead more likely that these changes are due to transcriptional changes within both myeloid- and lymphoid-committed progenitors. In this respect, MPP4 cells are still produced upon Daxx deletion (even with higher frequency upon acute Daxx loss in RosaCreERT2 mice; **Figure 2**) and at transcriptional level show a shift to a myeloid signature and repression of lymphoid-lineage driving TFs (**Figure 5j)**. Hence, we have revised our conclusions to reflect these new findings. We now suggest that lymphoid-prone multipotent progenitors are produced in Daxx-deficient mice but their potential may change towards myeloid commitment. The reduced differentiation towards B cells could be due to direct suppression of differentiation. In this respect, high Pu.1 expression has been suggested to promote myeloid over lymphoid differentiation in multipotent progenitors[16]. Pu.1 has also been shown to suppress B cell differentiation[17], again in agreement with the rescuing effect of Pu.1 loss in Daxx-deficient mice. Another (non-mutually exclusive) possibility is that mature B cells die or senesce upon Daxx loss, although we only saw marginal and not statistically significant changes in B-cell apoptosis at 3dpi (**Figure 2f**). It is also possible that lymphoid/B-cell committed progenitors could produce myeloid cells instead of B cells. In this respect, a number of studies have suggested that gain of myeloid TF expression, such as cEBPalpha can promote differentiation of lymphoid cells towards the myeloid lineage, as seminal work from Thomas Graf and others[18] has suggested. Finally, Daxx loss mostly affects follicular B cells, while marginal zone B cells remain mostly intact (**Figure 3h**), suggesting specific sensitivity of the former to Daxx inactivation. This and other aspects covered above will be an exciting avenue for future investigation.

<u>Comment 10</u>. The rescue data by Pu.1 appear incomplete as a group with Pu.1 deficiency alone is missing for data interpretation. In addition, the authors would need to show improvement of the HSPC phenotypes. Since Pu.1 is critical for myeloid cell differentiation, there is concern that Pu.1 deletion may be masking the phenotype rather than rescuing it.

Reply: We have used a comprehensive approach to address this important point. These are our main findings:

i) We ran transcriptomics of WT, Pu.1 KO, Daxx KO and DKO KLS cells and GMPs. These new sets of data show that in KLS cells, changes in transcriptome caused by Pu.1 loss only partially overlap with those caused by concomitant Daxx and Pu.1 loss (**Ext. Data Fig. 8a-g**), while the majority of changes differ, as reflected in the PCA plot and pathway analysis (**Figure 8a**; **Ext Data Fig. 8a-g**), suggesting an interaction between the two pathways. In GMPs, the number of changes unique to DKO is much smaller. See **Figure 5 for Reviewer below**.

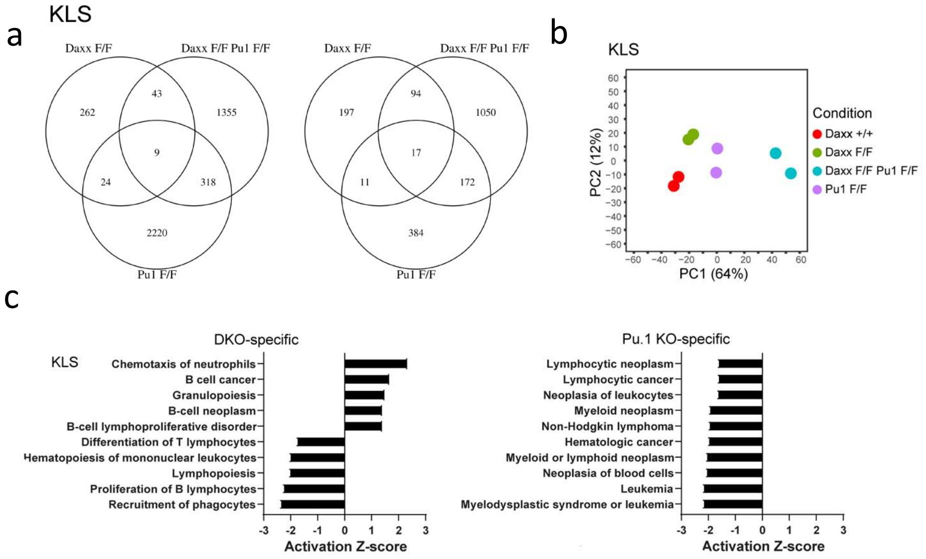

Figure 5 for Reviewer. Transcriptional changes unique to DKO cells suggest an interaction between the two pathways. **Panel a** shows upregulated (left) and downregulated genes (right) in the three different comparisons over WT KLS. **Panel b** shows PCA for WT, single KOs and DKO KLS cells. **Panel c** shows pathway analysis for DKO-specific and Pu.1 KO-specific pathways. Panels can be found in **Figure 8** and **Ext.Data Fig. 8** in the manuscript.

ii) We run CUT&Tag for H3.3 and Pu.1, along with a number of histone marks. The new data show that Daxx loss markedly affects both H3.3 and Pu.1 genome-wide distribution (**Figure 6**). Interestingly, our data suggest that i) there is no direct correlation between reduction in H3.3 chromatin association upon Daxx loss and gain of Pu.1 binding, but ii) actually Pu.1 chromatin binding directly correlates with H3.3 enrichment. This also suggests that in regions gaining both Pu.1 and H3.3 in Daxx-deficient cells H3.3 deposition is dependent on Hira or other H3.3 chaperones. For instance, enhancers losing H3.3 were close to genes that were more accessible, gained Pu.1 and H3.3 at their regulatory regions, changed their expression (**Figure 6c;** see below **Figure 6 for Reviewer**). Furthermore, there was altered Pu.1 binding at canonical Pu.1 target genes (**Figure 6e**), some of which were also upregulated in Daxx KO KLS cells (**Figure 5g-i**). These data suggest that i) Pu.1 targeting to chromatin may be dependent on H3.3, ii) that loss of H3.3 at enhancers may trigger Pu.1 binding and H3.3 loading at nearby promoters, and iii) gain of H3.3 at promoter regions in Daxx-deficient cells H3.3

deposition may be dependent on Hira (or other H3.3 chaperones). Finally, it is also interesting that there are many more distal regions in DKO HSPCs that gain H3K9me3 (7862) than those with significantly reduced H3K9me3 (1147) compared to both WT and Daxx KO HSPCs, again suggesting synergistic effects of concomitant loss of Daxx and Pu.1.

Figure 6 for Reviewer. H3K27ac, H3K9me3 and Pu.1 CUT&Tag read distribution across H3.3-depleted

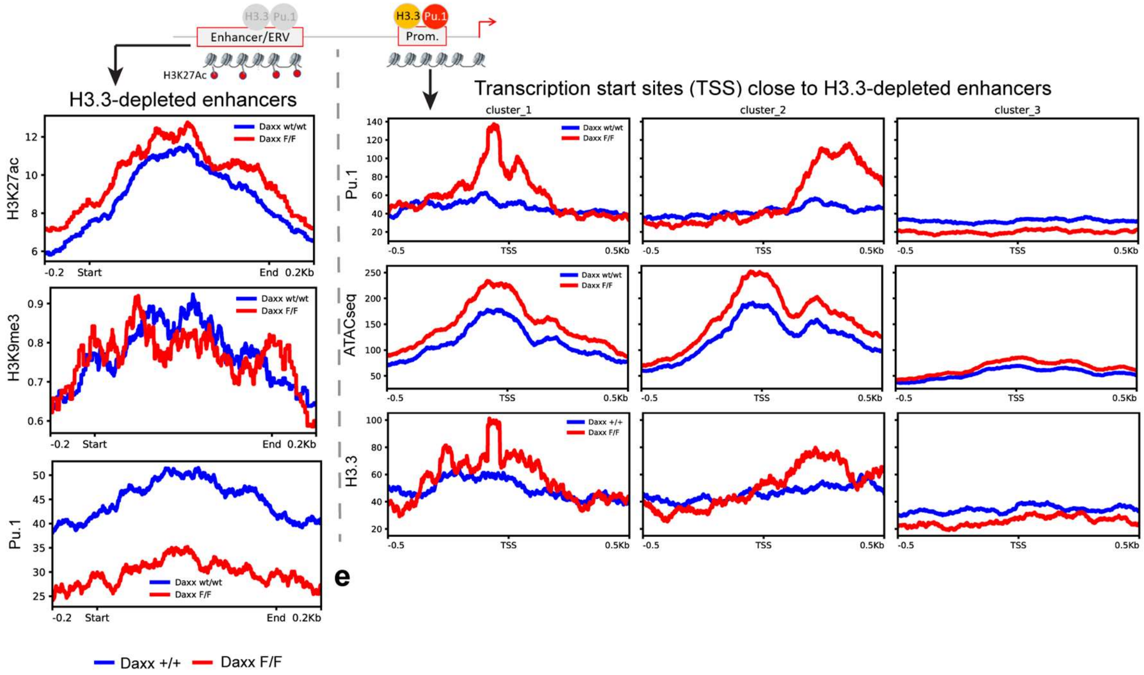

enhancers as well as Pu.1 (left), ATAC-seq and H3.3 coverage around TSS of genes close to H3.3-depleted enhancers (right). Pu.1 coverage were stratified into 3 clusters by k-means and ATAC-seq read distribution was plotted for the same clusters of TSS (right). Panels can be found in **Figure 6** in the manuscript

iii) We run FACS analysis on BM, spleen and PB from controls, Daxx KO, Pu.1 KO and DKO mice. These data show that while DKO cells displayed a significant decrease in neutrophil numbers compared to WT mice (**Figure 7a-i**), Pu.1 KO alone did not display changes in neutrophil numbers compared to WT (**Ext Data Fig. 7n,o**). B cells were not markedly altered in Pu.1 KO mice compared to WT. Previous work by Akashi, Tenen and coworkers reported substantial effects of acute Pu.1 deletion on hematopoiesis[16] with marked reduction in GMPs and granulocytes using the same Mx1-Cre model. However, in their study mice were injected with pI:pC two days after birth, while we only injected adult mice. It is therefore possible that adult myelopoiesis does not rely so much on Pu.1 at least at steady state compared to fetal and early postnatal hematopoiesis, unless in conditions of stress, such loss of Daxx and/or transplantation.

Overall, this whole new set of data suggest that the combination of Pu.1 and Daxx loss does not simply recapitulate changes seen in Pu.1 KO cells at both molecular and phenotypic level, suggesting that indeed Daxx- and Pu.1-dependent pathways functionally intersect for regulation of hematopoiesis.

Comment 11. Could the authors confirm that H3.3 expression is ablated in Daxx-deficient HSPCs?"

Reply: We kindly point out that while Daxx expression is downregulated upon H3.3 loss[11], little is known about whether H3.3 expression is affected by Daxx loss based on the existing literature. We have now performed WBs on Lineage- and Lineage+ WT and Daxx KO cells, which show clear loss of Daxx and no clear changes in H3.3 levels (**Ext. Data Fig. 2d**), as also observed in Daxx KO B220+ cells (**Ext. Data Fig. 3d**). However, given the role of Daxx as H3.3 chaperone, it was important to link its inactivation to changes in H3.3 deposition, a point also raised by Reviewer 3. To this end, we run CUT&Tag for H3.3 in WT and Daxx KO HSPCs and could show clear changes in H3.3 distribution, with many distal regions and enhancers displaying reduced H3.3 chromatin levels (**Figure 6a; see below Figure 7 for Reviewer**). Notably, concomitant deletion of Daxx and Pu.1 reverted the drop in H3.3 levels at enhancers and ERVs, to higher enrichment compared to WT cells (**Figure 8k**). This is further elaborated below in our response to Reviewer 3.

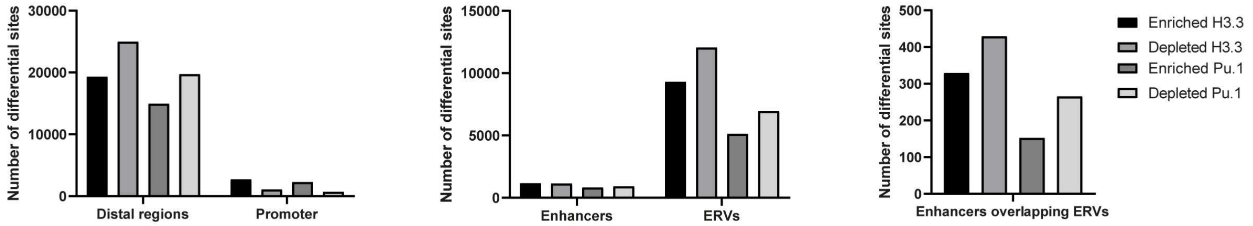

Figure 7 for Reviewer. Overview of altered H3.3 distribution and Pu.1 binding in Daxx single KO HSPCs determined by CUT&Tag assays. Panels can be found in **Figure 6** in the manuscript

Comment 13. In Fig.2a the authors state that deletion of Daxx induces increase of mature and immature granulocytes in bone sections. The authors need to provide quantification of this data and support it using flow cytometry analysis of granulocyte populations.

Reply: we have now shown relevant data on neutrophil mature and immature markers in Figure 2j. Flow cytometry analysis of neutrophils and neutrophil progenitors showed a significant increase of immature and mature neutrophil frequencies in relation to live cells. However, there is no difference in the frequency of immature or mature neutrophils over all neutrophil-like cells (Gr1+,Cd11b+ cells).

Comment 14. To support the finding of reduced erythropoiesis, expansion of WBCs (presented in Extended Data Fig. 2d) and expanded Megakaryocytes (Fig. 3b), the

authors should perform blood counts and at least report absolute numbers of RBC, WBCs and Platelets.

Reply: We have performed analysis of WBCs, RBCs and platelets using Sysmex (**Ext Data Fig. 4c**). We failed to record significant changes in RBCs, thus suggesting that extramedullary erythropoiesis observed in spleen is sufficient to sustain RBC levels in the PB. The increase in neutrophils might be balanced by loss of B cells, given that there is an increase in total WBCs but it does not reach significance. Finally, there is trend towards increased platelets in the Daxx KO PB but again it did not reach significance.

Comment 15. The authors should increase the n for data presented in Extended Data Fig.4, n of 3 is not enough to reach significance, especially given the variability of data and lack of statistical significance that is reported."

Reply: This is now discussed above and covered in **new Figure 2**.

Comment 18. The authors should justify why they performed RNAseq on LSK cells derived from chimeric mice following BM transplantation and not directly from DaxxF/F/ Mx-1 Cre mice at steady state, as these mice exhibit strong phenotypes already. Transplantation stress and active HSPC proliferation following transplantation may exacerbate the changes in gene expression upon Daxx deletion.

Reply: We have now run transcriptomics analysis on Daxx WT and KO KLS cells at 3wpi and 24wpi (steady state hematopoiesis) and compared these to the RNAseq data run on transplanted cells. As shown in **Figure 5**, pseudotime analysis shows that at 3wpi Daxx KO KLS cells display less marked changes in gene expression compared to 24wpi. Notably, 24wpi Daxx KO cells are closer to Daxx KO cells in transplantation settings, suggesting that there is an increasing bias of HSPCs towards the myeloid lineage through time and in stress conditions. More specifically, Pu.1 targets are increasingly altered going from 3wpi to 24wpi and transplantation settings, with an increasing bias towards myeloid targets (**Figure 5g-l; see below Figure 8 for Reviewer**). It is worth noting that at 3wpi also lymphoid Pu.1 targets are upregulated in KLS, suggesting that a general Pu.1 program is engaged upon Daxx loss, which then becomes more restricted due to other intervening mechanisms acting at later time points (24wpi) or in stress conditions (transplantation). The increased myeloid nature of the Pu.1 program engaged by Daxx is potentially related to Pu.1 upregulation, which is most evident in transplantation settings (**Figure 5n**). This is in agreement with seminal work by Dan Tenen and others on the critical role played by Pu.1 dosage in cell fate choices during hematopoiesis.

In summary, Daxx loss affects the transcriptome of hematopoietic stem cells (**Figure 1**) and progenitors (**Figure 5**) at steady state. However, as the reviewer's suggested, stress/activation exacerbate gene expression changes in Daxx-deficient progenitors. Time from Daxx loss is another factor at play, potentially via contribution of other chromatin-associated mechanisms and/or via age-dependent remodeling of intrinsic and extrinsic signaling. Finally, changes in transcriptome towards increased expression of myeloid genes between 3wpi and 24wpi correlate with an increased accumulation of neutrophils in the periphery (**Figure 4g**), again suggesting an exacerbation of the

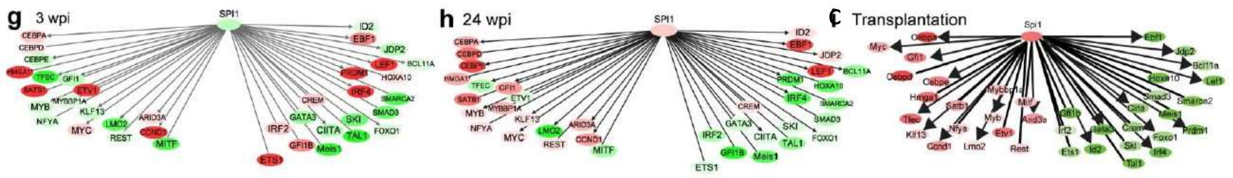

myeloid bias. a b

Figure 8 for Reviewer . IPA summaries showing progressive engagement of Pu.1-mediated myeloid program (left side of each summary) over lymphoid (right side), 3wpi (**a**), 24wpi (**b**) and transplantation settings (**c**). Panels can be found in **Figure 5** in the manuscript

Comment 19. To strengthen their conclusion that PU.1 deletion partially rescues neutrophilia and B cell loss in DaxxF/F/ Mx-1 Cre mice (Extended Data Fig. 10h-k) the authors should include bone marrow data."

Reply: We absolutely agree with the reviewer that this should be shown. We have now added BM data, which show significant reduction in neutrophil frequency and number in DKO mice compared to Daxx KO animals (**Figure 7c; see below Figure 9 for Reviewer**). Furthermore, this correlated with decreased GMP frequency but not number (**Figure 7b**). B cells are not significantly altered in DKO vs Daxx KO, either number- or frequency-wise in the BM (**Figure 7c**). In contrast, in the spleen we could detect a partial and significant rescue of B cell frequency and number (**Figure 7e**), which correlated to normalization of histological spleen appearance (**Fig. 7j**) and normalization of neutrophil frequency and number (**Figure 7e**).

BM Spleen

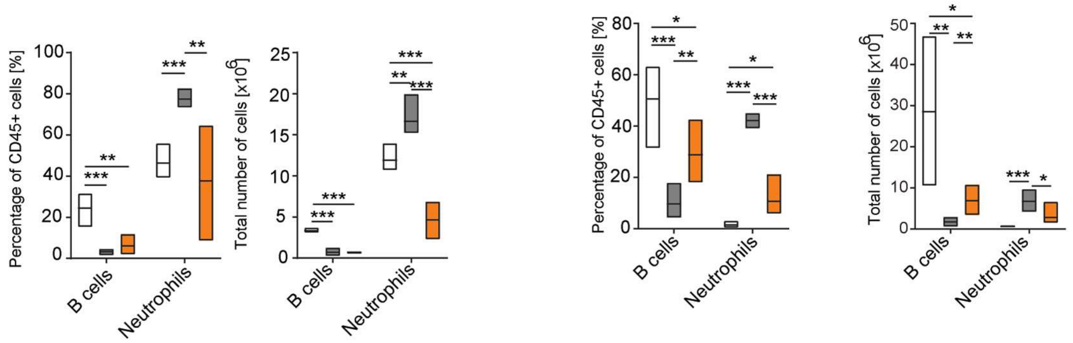

Figure 9 for Reviewer. Neutrophil frequency and number are normalized in DKO BM and spleen. While B cells are not significantly rescued in the BM of DKO mice, their frequency and number is in part rescued in the spleen. (**a**), 24wpi (**b**) and transplantation settings (**c**). Panels can be found in **Figure 7** in the manuscript

<u>Point-by-point response to Referee 3:</u>

<u>Comment 1.</u> DAXX is a H3.3 histone chaperone but no data are provided to determine whether its role in hematopoiesis is actually directly linked to its chaperone activity. It would have been important to explore this aspect. The authors found that enhancers (overlapping or not with ERVs) exhibit higher accessibility upon DAXX loss in HSCs (by ATAC-seq). Are these enhancers known to be enriched in H3.3 in HSCs?

Reply: We thank the reviewer for raising this point, which would allow us to link Daxx function in blood differentiation to H3.3 deposition. This is important, since previous work in the literature has suggested that Daxx silences ERVs via its binding to H3.3 but H3.3 loading may not be necessary[11]. To address this point, we have performed ATAC-seq along with CUT&Tag for H3.3, Pu.1 and selected histone marks in Mx1Cre animals. Given that number of LT-HSCs is a limiting factor, we studied distribution of H3.3, Pu.1 and histone marks in HSPCs cells (Lineagenegative/c-Kit-positive cells collected by MACS; see summary table for all genomics below: data produced during revision in grey).

Summary table for genomics

Mouse line	Genotype	Celltype	Time after last treatment	Number of mice	Genomics Assay	Antibody targets
ROSA-CRE-ERT2	Daxx +/+	LTHSC	3 days	2	RNA-seq	NA
ROSA-CRE-ERT2	Daxx +/+	LTHSC	3 days	2	ATAC-seq	NA
ROSA-CRE-ERT2	Daxx F/+	LTHSC	3 days	2	RNA-seq	NA
ROSA-CRE-ERT2	Daxx F/F	LTHSC	3 days	3	RNA-seq	NA
ROSA-CRE-ERT2	Daxx F/F	LTHSC	3 days	3	ATAC-seq	NA
ROSA-CRE-ERT2	Daxx +/+	LTHSC	3 wpi	2	ATAC-seq	NA
ROSA-CRE-ERT2	Daxx +/+	CMP	3 wpi	2	ATAC-seq	NA
ROSA-CRE-ERT2	Daxx +/+	GMP	3 wpi	2	ATAC-seq	NA
ROSA-CRE-ERT2	Daxx F/F	LTHSC	3 wpi	2	ATAC-seq	NA
ROSA-CRE-ERT2	Daxx F/F	CMP	3 wpi	2	ATAC-seq	NA
ROSA-CRE-ERT2	Daxx F/F	GMP	3 wpi	2	ATAC-seq	NA
Mx1-CRE	Daxx +/+	KLS	3 wpi	2	RNA-seq	NA
Mx1-CRE	Daxx +/+	KLS	3 wpi	2	ATAC-seq	NA
Mx1-CRE	Daxx F/F	KLS	3 wpi	2	RNA-seq	NA
Mx1-CRE	Daxx F/F	KLS	3 wpi	2	ATAC-seq	NA
Mx1-CRE	Daxx F/F Pu.1 F/F	KLS	3 wpi	2	RNA-seq	NA
Mx1-CRE	Daxx F/F Pu.1 F/F	KLS	3 wpi	2	ATAC-seq	NA
Mx1-CRE	Pu.1 F/F	KLS	3 wpi	2	RNA-seq	NA
Mx1-CRE	Daxx +/+	GMP	3 wpi	2	RNA-seq	NA
Mx1-CRE	Daxx F/F	GMP	3 wpi	2	RNA-seq	NA
Mx1-CRE	Daxx F/F Pu.1 F/F	GMP	3 wpi	2	RNA-seq	NA
Mx1-CRE	Pu.1 F/F	GMP	3 wpi	2	RNA-seq	NA
Mx1-CRE	Daxx +/+	MPP3	3 wpi	2	RNA-seq	NA
Mx1-CRE	Daxx F/F	MPP3	3 wpi	2	RNA-seq	NA
Mx1-CRE	Daxx +/+	MPP4	3 wpi	2	RNA-seq	NA
Mx1-CRE	Daxx F/F	MPP4	3 wpi	2	RNA-seq	NA
Mx1-CRE	Daxx +/+	KLS	24 wpi	2	RNA-seq	NA
Mx1-CRE	Daxx F/F	KLS	24 wpi	2	RNA-seq	NA
Mx1-CRE	Daxx F/F Pu.1 F/F	KLS	24 wpi	2	RNA-seq	NA
Mx1-CRE	Daxx +/+	GMP	24 wpi	2	RNA-seq	NA
Mx1-CRE	Daxx F/F	GMP	24 wpi	2	RNA-seq	NA
Mx1-CRE	Daxx F/F Pu.1 F/F	GMP	24 wpi	2	RNA-seq	NA
Mx1-CRE	Daxx +/+	KLS	Transplantation	2	RNA-seq	NA
Mx1-CRE	Daxx F/F	KLS	Transplantation	2	RNA-seq	NA
Mx1-CRE	Daxx +/+	GMP	Transplantation	2	RNA-seq	NA
Mx1-CRE	Daxx F/F	GMP	Transplantation	2	RNA-seq	NA
Mx1-CRE	Daxx +/+	HSPC	3 wpi	2	CUT&Tag	H3.3, Pu.1, H3K9me3, H3K27ac
Mx1-CRE	Daxx F/F	HSPC	3 wpi	2	CUT&Tag	H3.3, Pu.1, H3K9me3, H3K27ac
Mx1-CRE	Daxx +/+	HSPC	8 wpi	3	CUT&Tag	H3K9me3, H3K27me3
Mx1-CRE	Daxx F/F	HSPC	8 wpi	3	CUT&Tag	H3K9me3, H3K27me3
Mx1-CRE	Daxx F/F Pu.1 F/F	HSPC	8 wpi	3	CUT&Tag	H3K9me3, H3K27me3
Mx1-CRE	Daxx +/+	HSPC	24 wpi	2	CUT&Tag	H3.3, Pu.1, H3K9me3, H3K27ac, H3K27me3
Mx1-CRE	Daxx F/F	HSPC	24 wpi	2	CUT&Tag	H3.3, Pu.1, H3K9me3, H3K27ac, H3K27me3
Mx1-CRE	Daxx F/F Pu.1 F/F	HSPC	24 wpi	2	CUT&Tag	H3.3, H3K9me3, H3K27ac, H3K27me3
New datasets						

This large body of data has provided key insights into the effect of Daxx loss on chromatin accessibility, histone mark enrichment and H3.3 distribution:

i) Daxx loss indeed affects H3.3 distribution. Interestingly, distal regions can be divided in those that lose and those that acquire H3.3, with the former being more represented. Among them, ERVs and enhancers overlapping with ERVs tend to lose H3.3 (**Figure 6a**). In contrast, more promoters display increased H3.3 levels than those that show decreased enrichment.

ii) Daxx loss affects Pu.1 distribution too, with changes in a similar direction as changes to H3.3 distribution (**Figure 6a; see below Figure 1 for Reviewer**)

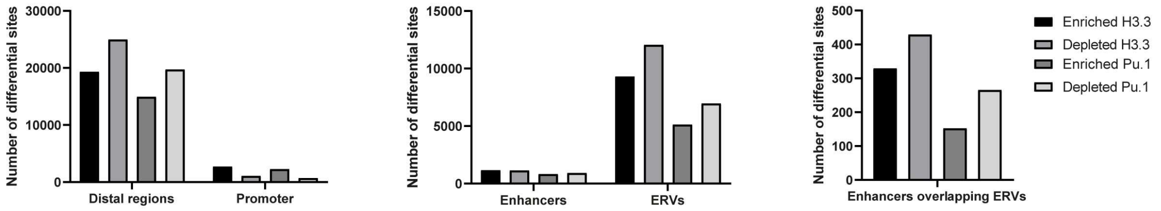

Figure 1 for Reviewer. Overview of altered H3.3 distribution and Pu.1 binding in Daxx single KO HSPCs determined by CUT&Tag assays. Panels can be found in **Figure 5** in the manuscript

iii) Enhancers overlapping ERVs and displaying decreased H3.3 levels are typified by increased chromatin accessibility and augmented H3K27Ac (**Figure 6b**).

iv) Enhancers showing reduced H3.3 also show reduced Pu.1 binding (**Figure 6c; see below Figure 2 for Reviewer**). However, neighboring genes display increased Pu.1 binding and increased accessibility, suggesting that reduced H3.3 levels at enhancers may promote enhancer/gene interactions resulting in increased chromatin opening and TF binding (**Figure 6c**). In general, this along with what shown in **Figure 6a** suggests that Pu.1 binding to chromatin may in part depend on H3.3 and that gaining of H3.3 at promoters that also gain

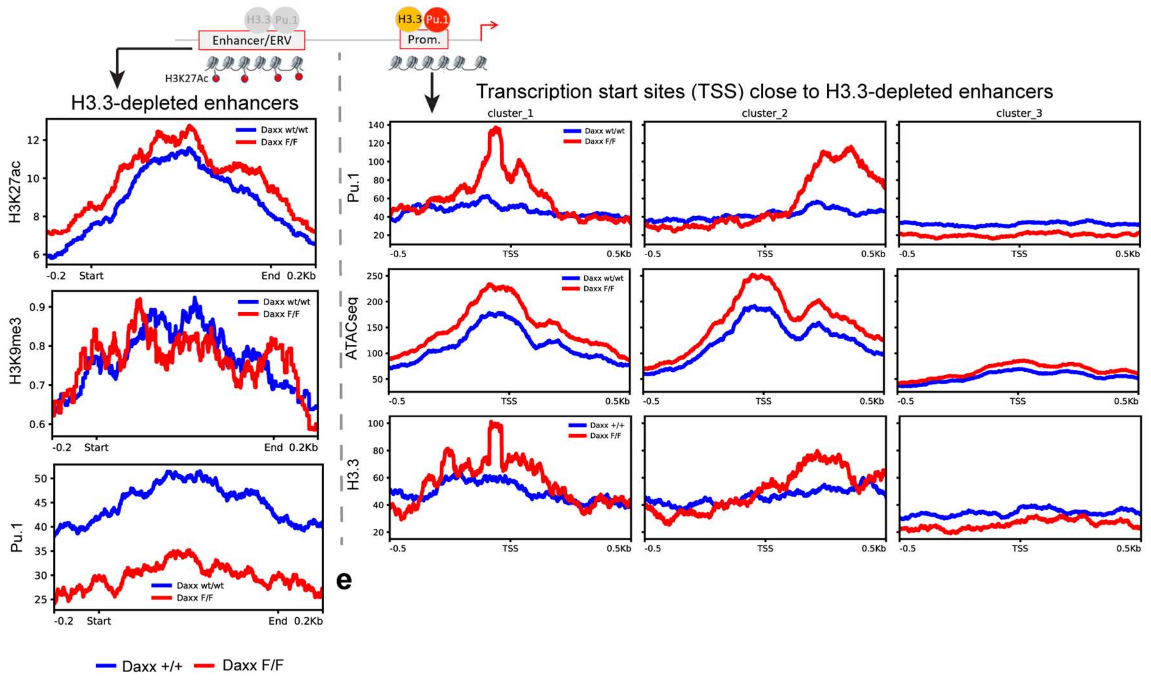

Figure 2 for Reviewer. H3K27ac, H3K9me3 and Pu.1 CUT&Tag read distribution across H3.3-depleted enhancers as well as Pu.1 (left), ATAC-seq and H3.3 coverage around TSS of genes close to H3.3-depleted [illegible]overage were stratified into 3 clusters by k-means and [illegible]ion was plotted for the same clusters of TSS (right). Panels [illegible] in the manuscript

Pu.1 in Daxx KO cells is dependent Hira or other chaperones.

v) There is also a subset of enhancers and ERVs that display increased H3K27ac without any concomitant depletion of H3.3 (**Figure 6h,i**). Interestingly, those regions display higher H3.3 levels than those that show increased H3K27ac and decrease in H3.3. In addition, enhancers and ERVs with both increase of H3K27ac and decrease of H3.3

reside in larger regions displaying H3.3 depletion.

Together, these new data suggest that indeed Daxx loss results in reduced H3.3 at a number of distal regions and enhancers. However, H3.3 is also gained at promoters, suggesting a previously underappreciated engagement of other H3.3 loading mechanisms in the absence of Daxx. Our work during revision also highlights an intriguing direct correlation between H3.3 levels and Pu.1 binding. Finally, our data suggest that Daxx deletion may also affect enhancer function independently of H3.3 loading, as suggested in the literature.

<u>Comment 2.</u> As the general repression of ERVs has been linked to H3K9me3 mark in ESCs, it would have been useful to examine whether higher accessible ERVs in DAXXf/f HSCs correlate or not with a decrease of H3K9me3. Do the enhancers non-overlapping with ERVs and displaying changes in chromatin accessibility upon DAXX loss correlate with a change in H3 post-translational modifications?

Reply: We have studied H3K9me3 distribution in relation to changes in H3.3 and chromatin accessibility using CUT&Tag (see also above). These are our main findings:

i) Daxx loss is associated with both gains and losses of H3K9me3 at distal regions.

ii) Zooming in on enhancers gaining accessibility, we found that enhancers overlapping with ERVs

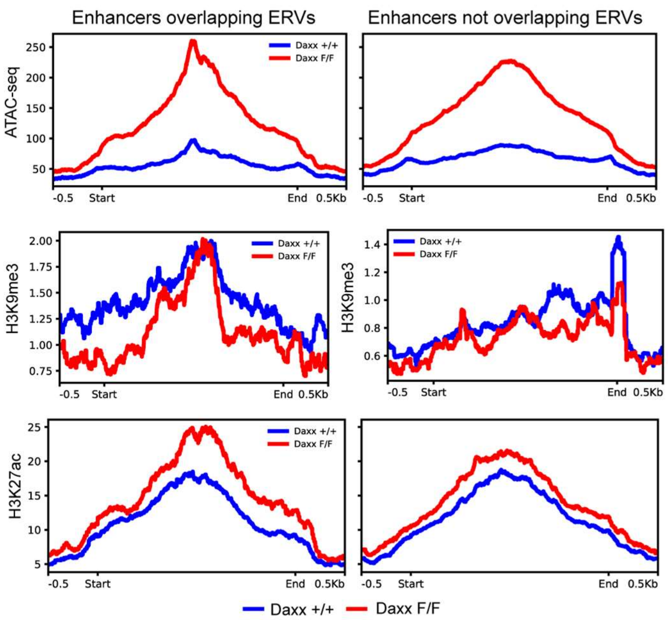

Figure 3 for Reviewer. Enrichment plots for ATAC -seq, H3K9me3 CUT&Tag and H3K27ac CUT&Tag at enhancers overlapping and not overlapping ERVs with increased accessibility. Panels can be found in **Figure 6** in the manuscript

show somewhat decreased H3K9me3, while there is not much change at enhancers not overlapping ERVs **(Figure 6f; see below Figure 3 for Reviewer)**. However, we also see a

stronger increase in H3K27Ac at enhancers overlapping ERVs than those not overlapping ERVs (**Figure 6f).** iii) Distal ERV regions with increased accessibility are associated with markedly increased H3K27ac and little or no changes in H3K9me3 **(Figure 6g)**.

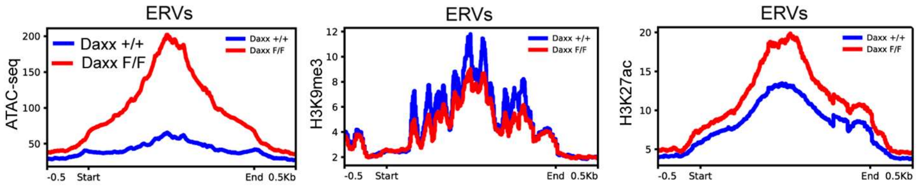

iv)

Reviewer.
for ATAC-

Figure 6

These findings suggest that the relationship between H3.3, H3K9me3 and enhancer marks is quite complex and cannot be reduced to a simple direct correlation. In this respect, we would like to bring the *Fbp1* and *Fbp2* locus as example of changes at chromatin level in Daxx KO and DKO progenitors (**see Figure 8l; see below Figure 5 for Reviewer**). This locus encodes for two critical regulators of gluconeogenesis that have been suggested to contribute to HSC/HSPC maintenance and are under the control of Setdb1-mediated H3K9 methylation[12]. Alterations of *Fbp* gene expression may results in changes in HSC/progenitor metabolism, which could have implications for HSC biology and differentiation.

Figure 5 for Reviewer. Genome browser coverage plot of the *Fbp1* and *Fbp2* locus. Panel can be found in **Figure 8** in the manuscript. Our main findings are:

i) *Fbp1* is upregulated upon Daxx loss in KLS at 24wpi, while *Fbp2* remains silenced. *Fbp1* upregulation is even more marked in transplantation conditions. In DKO cells, *Fbp1* expression is restored to WT levels, suggesting that its upregulation relies on Pu.1.

ii) There are three main regions that are bound by Pu.1 in KLS cells at this locus, two of which are known enhancers. Upon Daxx loss, there is increase in H3K27Ac at all of these regions, a phenomenon that is reverted in DKO cells. While H3.3 is present at these regulatory regions, Daxx

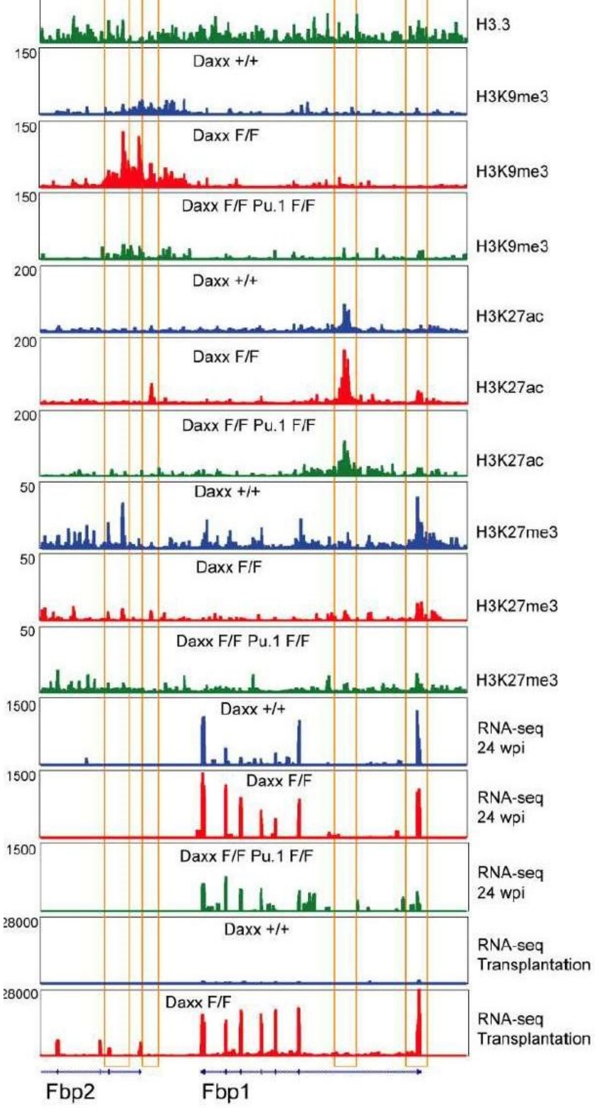

loss mostly abrogates H3.3 levels at the enhancer most proximal to *Fbp1*. Interestingly, H3.3 is restored at this region in DKO cells, suggesting that another H3.3 chaperone, like Hira may be recruited upon Pu.1 loss.

iii) The enhancer most proximal to *Fbp1* display almost undetectable H3K9me3 enrichment in WT cells, none in Daxx KO cells and a little in DKO cells. Notably, there is a large block of H3K9 trimethylation over the *Fbp2* gene and its regulatory regions, which was previously reported to be abrogated in Setdb1-deficient cells and linked to *Fbp2* upregulation in HSPCs[12]. Interestingly, H3K9me3 is further enriched at this region in Daxx-deficient cells (but not in DKO cells), suggesting that the reported interaction between Daxx and the Setdb1/Kap1 complex[11] does mediate H3K9 trimethylation at this locus and it is even possible that Daxx inhibits it. In agreement with these findings, Daxx loss results in *Fbp1* upregulation only. In contrast, Setdb1 loss mostly affects *Fbp2* expression[12].

iv) Finally, H3K27me3 is substantially reduced across the entire locus in Daxx KO and DKO KLS cells, suggesting that this epigenetic mark (not H3K9me3) could be involved in repression of the enhancer at the 5' of *Fbp1*.

<u>Comment 3.</u> Upon DAXX loss, it is not clear to which extent changes in ATAC-seq data (Figure 1) correlate with changes in transcriptomic data (Figure 5). For example, the authors pointed out two master regulators of myelopoiesis, Myb and Cebpa, displaying higher opening of chromatin at enhancer elements in DAXX KO HSCs (Fig. 1k). However, it is unclear whether this is a general observation and if the other upregulated regulators

involved in blood differentiation (shown in Extended Data Fig.9) also exhibit an opening of chromatin at enhancer elements upon DAXX loss?"

Reply: We thank the reviewer for raising these important points. To address the first point, we have chosen a comprehensive approach that is summarized below:

i) Transcriptomics and new ATAC-seq on LT-HSCs from RosaCreER mice upon acute Daxx deletion (3dpi). As shown in **Figure 1m**, cluster 1 shows increased transcription at genes displaying increased chromatin accessibility in Daxx-deficient LT-HSCs. Examples are *Mki67*, *Mx1*, *Irf7*, the *Ifit* cluster (**Figure 1o,p; Ext. Data Fig. 1o; see below Figure 6 for Reviewer**).

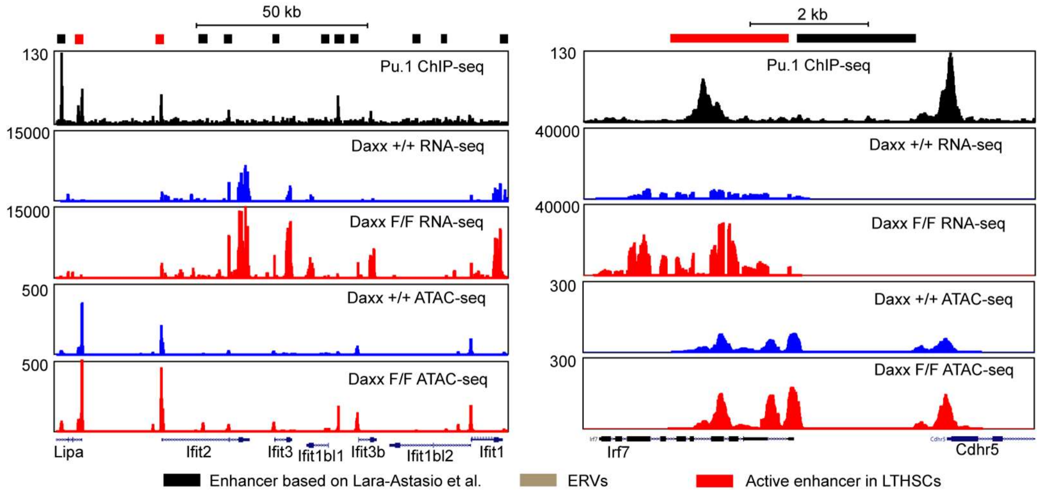

Figure 6 for Reviewer. Coverage plots for two of the genes displaying alterations in chromatin accessibility and expression. Panels can be found in **Figure 1** in the manuscript

ii) Transcriptomics and ATAC-seq data run on KLS cells from Mx1Cre mice show correspondence between enhancers that are opening up in Daxx KO cells and expression of neighboring genes (**Figure 8h,i**). Interestingly, both chromatin accessibility (**Figure 8g**) and gene expression are substantially reverted in DKO cells. As discussed above, we have also shown that there is increased Pu.1 and H3.3 levels at promoter regions displaying increased accessibility and close to enhancers that lose both H3.3 and Pu.1 (**Figure 6c; see above Figure 2 for Reviewer**). Many of the genes that acquire Pu.1 binding in Daxx KO cells also display altered gene expression (compare **Figure 6e** with **Figure 5g-i**).

<u>Comment 4.</u> The authors found that the master regulator of hematopoiesis PU.1/Spi-1, plays a major role in changes upon DAXX loss due to an overall higher expression of its target genes. They showed that PU.1/Spi-1, in absence of DAXX, is upregulated in both multipotent progenitors KLS and granulocyte-monocyte progenitors (GMPs). However, it

is not clear how PU.1/Spi-1 itself is upregulated in absence of DAXX. For example, what are the the data obtained by ATAC-seq at this gene and its regulatory elements?

Reply: We agree with the reviewer that this is an important point. We used ATAC-seq to study chromatin accessibility at the enhancer and promoter regions of Pu.1 (3wpi) in KLS cells. This was correlated to Pu.1 transcription at 3wpi, 24wpi and in transplantation settings (**Figure 5n**). Our main conclusions following analysis of these data are (see also **Figure 7 for Reviewer**):

i) At 3wpi, Pu.1 regulatory regions are clearly more accessible but its transcription is not yet increased (it is actually slightly reduced; **Figure 5n)** ii) Pu.1 induction is more marked in transplantation settings (**Figure 5n**) iii) Pu.1 transcriptional program is increasingly engaged through time (3wpi to 24wpi) and in stress conditions (i.e. transplantation; **Figure 5g-i**) and becomes more myeloid-biased.

In conclusion, we propose that the increase in Pu.1 transcription is not the main mechanism for the engagement of a Pu.1 program upon Daxx loss, but it is probably a mechanism for strengthening its myeloid fate-inducing role at later time points from induction in steady state hematopoiesis or in stress conditions (transplantation).

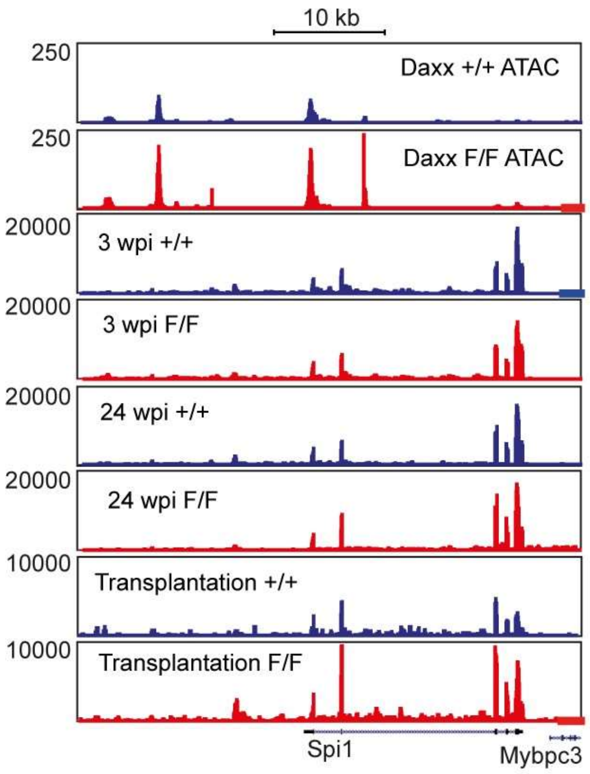

Figure 7 for Reviewer (ref. to Figure 5n). Coverage plot of the Spi1 locus at different time points from Daxx deletion and in transplantation settings (showing expression and chromatin landscape). Panel can be found in **Figure 5** in the manuscript

<u>Comment 5.</u> Related to Figure 4: Approximately 15% of DAXX f/f mice developed skin lesions (this corresponds to how many mice?). Due to this low percentage, it is perhaps difficult to conclude that "DAXX loss leads to systemic inflammation and neutrophilic skin disease resembling human pyoderma gangrenosum (PG)"? Do the authors have additional arguments ?

Reply: The incidence of the neutrophilic skin disease is not high (11 out of 66 mice, 7/32 females and 4/34 males, **see Figure 4b**), but we cannot exclude that systematic analysis of skin in Daxx KO animals could reveal changes in tissue homeostasis that are not evident at macroscopic level. We feel this is beyond the scope of our manuscript, especially in view of the fact that Daxx-deficient animals show a

systemic inflammation phenotype irrespective of the present of skin lesions based on analysis of pro-inflammatory cytokines, as also shown in the previous version of the manuscript. In order to provide more insights into the inflammatory state of Daxx-deficient mice, we have now extended analysis of proinflammatory cytokines at two time points (3wpi and 8wpi). As mentioned above in our response to Reviewer 2, we show significant increase in IFN-gamma, along with IL-23, MCP-1, IL-6, IL-17A. Notably, a number of these cytokines were normalized in Daxx/Pu.1 DKO mice, with significant reductions for IL-23 and IFN-gamma (**see Figure 7k,l**). Interestingly, in 8wpi Daxx KO mice we observed normalization of IFN-gamma accompanied by significant increase in IL-10, IL-12, IL-27, IFNbeta, with the latter two being normalized in DKO mice. Overall, these data suggest changes in inflammatory cytokine profiles through time from Daxx deletion. Finally, the observed normalization of the cytokine profiles in DKO animals, which display reduced peripheral neutrophils (**see Figure 7e,g**) further support the link between neutrophilia and inflammation.

Comment 6. The authors argue that "loss of HIRA does not markedly affect hematopoietic cell composition". However, the percentage of cells for several cell types appeared significantly changed upon HIRA loss (Extended Data Fig. 6). Perhaps these changes are less striking than upon DAXX loss, but still significant. This needs to be commented."

Reply: We thank the reviewer to point at the changes seen in HIRA-deficient mice. Notably, we were able to show increased levels of Daxx in Hira KO mice (see **Ext. Data Figure 5c; see below Figure 8 for Reviewer**), suggesting that Daxx may be able to compensate for some of the changes caused by Hira loss. These points have been covered in our discussion section.

Figure 8 for Reviewer. Daxx levels are increased in Hira-deficient hematopoietic progenitors. Panels can be found in **Ext Data Fig. 5** in the manuscript

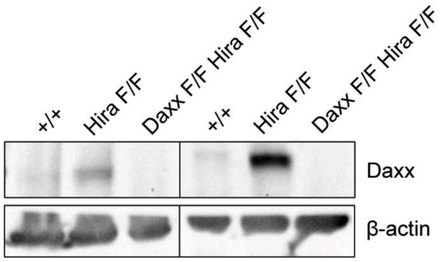

Comment 7. When a Western blot for DAXX is provided, H3.3 should be also shown, as the amounts of a histone variant and its chaperone have been previously reported to be co-regulated. Western blot for HIRA should be shown for HIRA KO.

Reply: It is indeed correct that Daxx expression is affected by H3.3 loss in other models (see work by Peter Lewis and collaborators[11]). However, little is known about how H3.3 expression is affected by Daxx loss. We have now performed WBs on Lin- WT and Daxx KO cells, which show clear loss of Daxx

and no substantial changes in H3.3 levels (**Ext. Data Fig. 2d**). Same for BM B220+ cells (**Ext. Data Fig. 3d**). However, we haven't managed to get meaningful data with both commercially available antibodies and those provided by Peter Adams, a co-author on the paper. Therefore, we can only rely on QPCR data (**Ext. Data Fig. 5b**).

<u>Comment 8.</u> A cartoon summarizing the current model for the role of DAXX in the hematopoietic lineage and what happens upon loss of DAXX would help.

Reply: We have now included a cartoon summarizing the current model for the role of Daxx in hematopoiesis (**Ext. Data Fig. 8h; see below Figure 9 for Reviewer**).

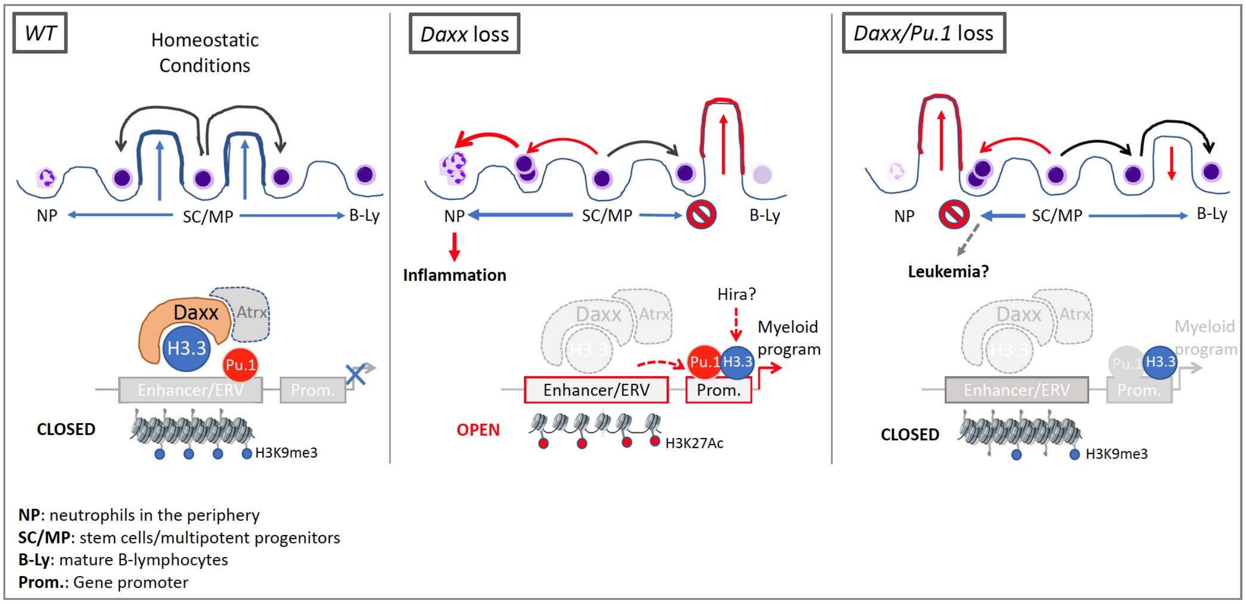

Figure 9 for Reviewer. Proposed model: Daxx contributes to epigenetic barriers restricting fate/identity of hematopoietic stem cells and progenitors thus contributing to balanced differentiation output. Upon Daxx loss, stem cells enter differentiation and produce both myeloid and lymphoid-biased progenitors. However, while myeloid differentiation towards neutrophils is enhanced leading to inflammation, generation of mature B cells is strongly impaired. This work implicates a previously unknown link between Daxx and the pioneer TF Pu.1 for regulation of myeloid vs lymphoid balance during hematopoiesis. Daxx loss leads to gain of H3K27Ac at enhancers. This is associated with reduction in both H3.3 and Pu.1 levels at the same enhancers, and their accumulation at neighboring genes, correlating with changes in gene expression. These data also suggest that H3.3 loading at promoters gaining Pu.1 binding in Daxx KO cells may be dependent on other H3.3 chaperones such as Hira. Upon inactivation of the pioneer TF Pu.1, the block of B-cell differentiation is partly relieved, while peripheral accumulation of neutrophils is hampered. Given that Pu.1/Daxx DKO progenitors display unique chromatin and transcriptome features and that both Daxx and Pu.1 inactivation/repression are linked to myeloid leukemia, we hypothesize that loss of both genes may make progenitor cells susceptible to neoplastic transformation. Panel can be found in **Ext. Data Fig. 8**.

<u>Comment 9.</u> The authors have to discuss their results in view of the recent published data from Chen et al., 2020 about the role of the HIRA chaperone in hematopoiesis."

Reply: We apologize for not having already included these related bibliographies into our manuscript, while preparing for submission. However, we have now carefully reviewed the *Cell Reports* paper by C Chen, and have summarized our comments on the shared and different findings between Chen et al and our two studies below:

1. As a more general comment, C. Chen et al. reach conclusions on the role of Hira in *adult* hematopoiesis using a model whereby Hira inactivation occurs *in utero* (Vav-Cre). Although fetal hematopoiesis is spared in Vav-cre;Hira$^{fl/fl}$ mice, global epigenetic changes might have already occurred which could underlie the more pronounced phenotypes at adult stage. In this respect, a defect in the repopulation potential of fetal liver HSCs was observed in the Chen et al study, suggesting an underlying developmental defect. Instead, our two studies employ two different models (RosaCreER and Mx1-Cre) for gene inactivation in *adult* hematopoiesis, thus more properly assessing the role of H3.3 and its loading machinery in *adult* tissues. A minor point is that the *Hira^{floxed}* mice used by *NCB-G42551-T* have loxp sites flanking exon 4[1], whereas the *Hira^{floxed}* line used by Chen et al have loxp sites flanking exon 7. As a result, we believe differences in the observed phenotypes may also be due to the different models employed.
2. While anemic, Vav-Cre;Hira$^{fl/fl}$ mice display a small decrease in RBCs numbers. In the range of phenotypes which is the focus within our manuscript, Vav-Cre;Hira$^{fl/fl}$ mice did not manifest a myeloid skewing towards neutrophilic differentiation.
3. Finally, it is important to highlight that Daxx protein levels are increased in Hira KO cells (**Ext. Data Fig. 5c; Figure 8 for Reviewer above**), suggesting a degree of redundancy between the two chaperones. It is conceivable that increased Daxx levels may compensate for potential effects of Hira loss on the myeloid lineage. This requires further investigation as part of future studies.

This work has now been cited and briefly discussed in the revised version of our manuscript (**Results section**).

Minor points

To help readers that are not specialists of the hematopoietic lineage, a scheme showing the hematopoietic differentiation states and in particular including all the different cell types examined in the study should be provided.

Reply: we have now introduced schematics in Figure 1 to help non-specialists to better appreciate the key transitions during hematopoiesis.

Page 7, the authors should add references for the statement "Since several reports suggest a link between neutrophilia and inflammation…". Which reports ?

Reply: We now added relevant references to the Results section

Figure 1, it would be easier for the reader to indicate in the figure (and not only in the legend) that in panel g it is the ATAC-seq coverage across all chromosomes and in panel h across sex chromosomes.

Reply: these panels have now been amended as suggested (**Ext. Data Fig. 1g,h**)

NUMERICAL DATA TABLE

Numerical data table has been included in the submission documents

REFERENCES

1. Collins, P.L., Kyle, K.E., Egawa, T., Shinkai, Y. & Oltz, E.M. The histone methyltransferase SETDB1 represses endogenous and exogenous retroviruses in B lymphocytes. *Proc Natl Acad Sci U S A* **112**, 8367-8372 (2015).
2. Lefkopoulos, S. *et al.* Repetitive Elements Trigger RIG-I-like Receptor Signaling that Regulates the Emergence of Hematopoietic Stem and Progenitor Cells. *Immunity* **53**, 934-951 e939 (2020).
3. Sato, T. *et al.* Interferon regulatory factor-2 protects quiescent hematopoietic stem cells from type I interferon-dependent exhaustion. *Nat Med* **15**, 696-700 (2009).
4. Ashley, C.L., Abendroth, A., McSharry, B.P. & Slobedman, B. Interferon-Independent Upregulation of Interferon-Stimulated Genes during Human Cytomegalovirus Infection is Dependent on IRF3 Expression. *Viruses* **11** (2019).
5. Ashley, C.L., Abendroth, A., McSharry, B.P. & Slobedman, B. Interferon-Independent Innate Responses to Cytomegalovirus. *Front Immunol* **10**, 2751 (2019).
6. Hertzog, P.J., O'Neill, L.A. & Hamilton, J.A. The interferon in TLR signaling: more than just antiviral. *Trends in immunology* **24**, 534-539 (2003).
7. Pietras, E.M. *et al.* Re-entry into quiescence protects hematopoietic stem cells from the killing effect of chronic exposure to type I interferons. *J Exp Med* **211**, 245-262 (2014). 8. Hirche, C. *et al.* Systemic Virus Infections Differentially Modulate Cell Cycle State and Functionality of Long-Term Hematopoietic Stem Cells In Vivo. *Cell Rep* **19**, 2345-2356 (2017).

9. Beerman, I. *et al.* Functionally distinct hematopoietic stem cells modulate hematopoietic lineage potential during aging by a mechanism of clonal expansion. *Proc Natl Acad Sci U S A* **107**, 5465-5470 (2010).
10. Elsasser, S.J., Noh, K.M., Diaz, N., Allis, C.D. & Banaszynski, L.A. Histone H3.3 is required for endogenous retroviral element silencing in embryonic stem cells. *Nature* **522**, 240-244 (2015).
11. Hoelper, D., Huang, H., Jain, A.Y., Patel, D.J. & Lewis, P.W. Structural and mechanistic insights into ATRX-dependent and -independent functions of the histone chaperone DAXX. *Nat Commun* **8**, 1193 (2017).
12. Koide, S. *et al.* Setdb1 maintains hematopoietic stem and progenitor cells by restricting the ectopic activation of nonhematopoietic genes. *Blood* **128**, 638-649 (2016).
13. Min, I.M. *et al.* The transcription factor EGR1 controls both the proliferation and localization of hematopoietic stem cells. *Cell Stem Cell* **2**, 380-391 (2008).
14. Adrover, J.M. *et al.* A Neutrophil Timer Coordinates Immune Defense and Vascular Protection. *Immunity* **51**, 966-967 (2019).
15. Cossio, I., Lucas, D. & Hidalgo, A. Neutrophils as regulators of the hematopoietic niche. *Blood* **133**, 2140-2148 (2019).
16. Iwasaki, H. *et al.* Distinctive and indispensable roles of PU.1 in maintenance of hematopoietic stem cells and their differentiation. *Blood* **106**, 1590-1600 (2005).
17. Vigorito, E. *et al.* microRNA-155 regulates the generation of immunoglobulin classswitched plasma cells. *Immunity* **27**, 847-859 (2007).
18. Cirovic, B. *et al.* C/EBP-Induced Transdifferentiation Reveals Granulocyte-Macrophage Precursor-like Plasticity of B Cells. *Stem Cell Reports* **8**, 346-359 (2017).

Decision Letter, first revision:

Dear Paolo,

Your manuscript, "Aberrant chromatin landscape upon loss of the H3.3 chaperone Daxx leads to Pu.1–mediated neutrophilia and inflammation", has now been seen by 2 of our original referees.

Please note that referee 2 was unfortunately unable to re-review this manuscript and the response to these comments has instead been evaluated by referee 4 (expert on haematopoiesis).

As you will see from the combined feedback (attached below), the referees find this work much improved in revision, but they have raised some minor, remaining points. Although we continue to be very interested in this study, we believe that their concerns should be addressed before we can consider publication in Nature Cell Biology.

In particular, it would be important to provide additional discussion, streamline the text for a general audience and improve the presentation of some of the Figures, as highlighted by referees 1 and 3.

We therefore invite you to take these points into account when revising the manuscript. In addition, when preparing the revision please:

- ensure that it conforms to our format instructions and publication policies (see below and www.nature.com/nature/authors/).

- provide a point-by-point rebuttal to the full referee reports verbatim, as provided at the end of this letter.

- provide and updated Editorial Policy Checklist (found here https://www.nature.com/authors/policies/Policy.pdf) and Reporting Summary (found here https://www.nature.com/authors/policies/ReportingSummary.pdf). This is essential for reconsideration of the manuscript and these documents will be available to editors and referees in the event of peer review. For more information see http://www.nature.com/authors/policies/availability.html or contact me.

Please submit the revised manuscript files and the point-by-point rebuttal to the referee comments using this link:

[REDACTED]

*This url links to your confidential home page and associated information about manuscripts you may have submitted or be reviewing for us. If you wish to forward this email to co-authors, please delete the link to your homepage.

We would like to receive the revision within four weeks. If submitted within this time period, reconsideration of the revised manuscript will not be affected by related studies published elsewhere, or accepted for publication in Nature Cell Biology in the meantime. We would be happy to consider a revision even after this timeframe, but in that case we will consider the published literature at the time of resubmission when assessing the file.

We hope that you will find our referees' comments, and editorial guidance helpful. Please do not hesitate to contact me if there is anything you would like to discuss.

Best wishes,

Christine.

Christine Weber, PhD
Senior Editor
Nature Cell Biology
E-mail: christine.weber@nature.com
Phone: +44 (0)207 843 4924

Reviewers' Comments:

Reviewer #1:
Remarks to the Author:
Overall, the revised manuscript is significantly improved. The authors have done a great job. While the authors have adequately addressed all the questions raised previously, I do have a couple of additional comments.

Major issues:
The authors have not discussed deeply the relevance of their TERRA lncRNA-related analysis to the rest of their studies. The authors only described that TERRA lncRNA accessibility and expression are altered upon Daxx KO, but it was not clear how does this link to the rest of the studies. Do the authors think TERRA lncRNA contributes to the hematopoiesis dysregulation they observed, and how? Will it be likely to be Pu.1 dependent? If so, did the authors investigate whether Daxx/Pu.1 DKO could reverse TERRA upregulation?

Minor issues:
Figure legend
1) In the legend for Figure 1, point j describes Figure 1k, while point k describes Figure 1j.
2) In Figure 7j, it would help the reader if the left, middle and right panel are labelled (e.g WT, Daxx KO, DKO).
3) In the Extended Data Fig. 5b, the legend color for Daxx+/+ Hira+/+ (white) does not correspond to the bar color (black bar)
4) In Extended Data Fig. 7h, it would help the reader if the upper, middle and lower panel are labelled (e.g 3 wpi, 24 wpi, transplantation)

Reviewer #3:
Remarks to the Author:
In the revised version by Gerber et al, series of experiments have been added and an important work has been carried out to address issues raised by the three reviewers concerning the first submitted manuscript. Significant work has been carried out considering most questions but the outcome and interpretations remain still extremely difficult at this point in time to get a clear-cut conclusion, at least for a reviewer that is not a specialist of the hematopoietic system.
While they did document the fact that aberrant chromatin landscapes arise upon the loss of Daxx (under their experimental conditions) and that there is a Pu.1-mediated neutrophilia and inflammation, unfortunately it is still unclear about what is actually happening and what is the underlying mechanism. The additional work further emphasizes the difficulty to draw clear conclusions. However, they did provide a lot of data, this should be acknowledged. Furthermore, the authors were very cautious and balanced in their interpretations. Should specialist feel satisfied with the relevance for the hematopietic system, I could support publication. I would still recommend to make a serious effort to help readers by trying to screen their manuscript to clarify as much as possible the messages to reach out to a general audience in cell biology.

Reviewer #4:

Remarks to the Author:
Gerber et al identify a clear role for Daxx in maintaining the chromatin landscape of LT-HSCs, and associate this with aberrant gene expression. They have not (been able to) perform transplantion studies, but I feel that these are not absolutely required to substantiate their conclusions. In their paper they do not make any claims on the role of Daxx for HSC-specific functionality, so I do not believe that classical HSC (serial) transplantion expeirments are essential for this paper.

GUIDELINES FOR SUBMISSION OF NATURE CELL BIOLOGY ARTICLES

READABILITY OF MANUSCRIPTS – Nature Cell Biology is read by cell biologists from diverse backgrounds, many of whom are not native English speakers. Authors should aim to communicate their findings clearly, explaining technical jargon that might be unfamiliar to non-specialists, and avoiding non-standard abbreviations. Titles and abstracts should concisely communicate the main findings of the study, and the background, rationale, results and conclusions should be clearly explained in the manuscript in a manner accessible to a broad cell biology audience. Nature Cell Biology uses British spelling.

ARTICLE FORMAT

TITLE – should be no more than 100 characters including spaces, without punctuation and avoiding technical terms, abbreviations, and active verbs..

AUTHOR NAMES – should be given in full.

AUTHOR AFFILIATIONS – should be denoted with numerical superscripts (not symbols) preceding the names. Full addresses should be included, with US states in full and providing zip/post codes. The corresponding author is denoted by: "Correspondence should be addressed to [initials]."

ABSTRACT – should not exceed 150 words and should be unreferenced. This paragraph is the most visible part of the paper and should briefly outline the background and rationale for the work, and accurately summarize the main results and conclusions. Key genes, proteins and organisms should be specified to ensure discoverability of the paper in online searches.

TEXT – the main text consists of the Introduction, Results, and Discussion sections and must not exceed 3500 words including the abstract. The Introduction should expand on the background relating to the work. The Results should be divided in subsections with subheadings, and should provide a concise and accurate description of the experimental findings. The Discussion should expand on the findings and their implications. All relevant primary literature should be cited, in particular when discussing the background and specific findings.

ACKNOWLEDGEMENTS – should be kept brief. Professional titles and affiliations are unnecessary. Grant numbers can be listed.

AUTHOR CONTRIBUTIONS – must be included after the Acknowledgements, detailing the contributions of each author to the paper (e.g. experimental work, project planning, data analysis etc.). Each author should be listed by his/her initials.

FINANCIAL AND NON-FINANCIAL COMPETING INTERESTS – the authors must include one of three declarations: (1) that they have no financial and non-financial competing interests; (2) that they have financial and non-financial competing interests; or (3) that they decline to respond, after the Author Contributions section. This statement will be published with the article, and in cases where financial and non-financial competing interests are declared, these will be itemized in a web supplement to the article. For further details please see https://www.nature.com/licenceforms/nrg/competing-interests.pdf.

REFERENCES – are limited to a total of 70 in the main text and Methods combined,. They must be numbered sequentially as they appear in the main text, tables and figure legends and Methods and must follow the precise style of Nature Cell Biology references. References only cited in the Methods should be numbered consecutively following the last reference cited in the main text. References only associated with Supplementary Information (e.g. in supplementary legends) do not count toward the total reference limit and do not need to be cited in numerical continuity with references in the main text. Only published papers can be cited, and each publication cited should be included in the numbered reference list, which should include the manuscript titles. Footnotes are not permitted.

METHODS – Nature Cell Biology publishes methods online. The methods section should be provided as a separate Word document, which will be copyedited and appended to the manuscript PDF, and incorporated within the HTML format of the paper.

Methods should be written concisely, but should contain all elements necessary to allow interpretation and replication of the results. As a guideline, Methods sections typically do not exceed 3,000 words. The Methods should be divided into subsections listing reagents and techniques. When citing previous methods, accurate references should be provided and any alterations should be noted. Information must be provided about: antibody dilutions, company names, catalogue numbers and clone numbers for monoclonal antibodies; sequences of RNAi and cDNA probes/primers or company names and catalogue numbers if reagents are commercial; cell line names, sources and information on cell line identity and authentication. Animal studies and experiments involving human subjects must be reported in detail, identifying the committees approving the protocols. For studies involving human subjects/samples, a statement must be included confirming that informed consent was obtained. Statistical analyses and information on the reproducibility of experimental results should be provided in a section titled "Statistics and Reproducibility".

All Nature Cell Biology manuscripts submitted on or after March 21 2016, must include a Data availability statement as a separate section after Methods but before references, under the heading "Data Availability". For Springer Nature policies on data availability see http://www.nature.com/authors/policies/availability.html; for more information on this particular policy see http://www.nature.com/authors/policies/data/data-availability-statements-data-citations.pdf. The Data availability statement should include:

• Accession codes for primary datasets (generated during the study under consideration and designated as "primary accessions") and secondary datasets (published datasets reanalysed during the study under consideration, designated as "referenced accessions"). For primary accessions data should be made public to coincide with publication of the manuscript. A list of data types for which submission to community-endorsed public repositories is mandated (including sequence, structure, microarray, deep sequencing data) can be found here http://www.nature.com/authors/policies/availability.html#data.

• Unique identifiers (accession codes, DOIs or other unique persistent identifier) and hyperlinks for datasets deposited in an approved repository, but for which data deposition is not mandated (see here for details http://www.nature.com/sdata/data-policies/repositories).

• At a minimum, please include a statement confirming that all relevant data are available from the authors, and/or are included with the manuscript (e.g. as source data or supplementary information), listing which data are included (e.g. by figure panels and data types) and mentioning any restrictions on availability.

• If a dataset has a Digital Object Identifier (DOI) as its unique identifier, we strongly encourage including this in the Reference list and citing the dataset in the Methods.

We recommend that you upload the step-by-step protocols used in this manuscript to the Protocol Exchange. More details can found at www.nature.com/protocolexchange/about.

DISPLAY ITEMS – main display items are limited to 6-8 main figures and/or main tables. For Supplementary Information see below.

FIGURES – Colour figure publication costs $395 per colour figure. All panels of a multi-panel figure must be logically connected and arranged as they would appear in the final version. Unnecessary figures and figure panels should be avoided (e.g. data presented in small tables could be stated briefly in the text instead).

All imaging data should be accompanied by scale bars, which should be defined in the legend. Cropped images of gels/blots are acceptable, but need to be accompanied by size markers, and to retain visible background signal within the linear range (i.e. should not be saturated). The boundaries of panels with low background have to be demarked with black lines. Splicing of panels should only be considered if unavoidable, and must be clearly marked on the figure, and noted in the legend with a statement on whether the samples were obtained and processed simultaneously. Quantitative comparisons between samples on different gels/blots are discouraged; if this is unavoidable, it has be performed for samples derived from the same experiment with gels/blots were processed in parallel, which needs to be stated in the legend.

Figures should be provided at approximately the size that they are to be printed at (single column is 86 mm, double column is 170 mm) and should not exceed an A4 page (8.5 x 11"). Reduction to the scale that will be used on the page is not necessary, but multi-panel figures should be sized so that the whole figure can be reduced by the same amount at the smallest size at which essential details in each panel are visible. In the interest of our colour-blind readers we ask that you avoid using red and

green for contrast in figures. Replacing red with magenta and green with turquoise are two possible colour-safe alternatives. Lines with widths of less than 1 point should be avoided. Sans serif typefaces, such as Helvetica (preferred) or Arial should be used. All text that forms part of a figure should be rewritable and removable.

We accept files from the following graphics packages in either PC or Macintosh format:

- For line art, graphs, charts and schematics we prefer Adobe Illustrator (.AI), Encapsulated PostScript (.EPS) or Portable Document Format (.PDF). Files should be saved or exported as such directly from the application in which they were made, to allow us to restyle them according to our journal house style.

- We accept PowerPoint (.PPT) files if they are fully editable. However, please refrain from adding PowerPoint graphical effects to objects, as this results in them outputting poor quality raster art. Text used for PowerPoint figures should be Helvetica (preferred) or Arial.

- We do not recommend using Adobe Photoshop for designing figures, but we can accept Photoshop generated (.PSD or .TIFF) files only if each element included in the figure (text, labels, pictures, graphs, arrows and scale bars) are on separate layers. All text should be editable in 'type layers' and line-art such as graphs and other simple schematics should be preserved and embedded within 'vector smart objects' - not flattened raster/bitmap graphics.

- Some programs can generate Postscript by 'printing to file' (found in the Print dialogue). If using an application not listed above, save the file in PostScript format or email our Art Editor, Allen Beattie for advice (a.beattie@nature.com).

Regardless of format, all figures must be vector graphic compatible files, not supplied in a flattened raster/bitmap graphics format, but should be fully editable, allowing us to highlight/copy/paste all text and move individual parts of the figures (i.e. arrows, lines, x and y axes, graphs, tick marks, scale bars etc). The only parts of the figure that should be in pixel raster/bitmap format are photographic images or 3D rendered graphics/complex technical illustrations.

All placed images (i.e. a photo incorporated into a figure) should be on a separate layer and independent from any superimposed scale bars or text. Individual photographic images must be a minimum of 300+ DPI (at actual size) or kept constant from the original picture acquisition and not decreased in resolution post image acquisition. All colour artwork should be RGB format.

FIGURE LEGENDS – must not exceed 350 words for each figure to allow fit on a single printed NCB page together with the figure. They must include a brief title for the whole figure, and short descriptions of each panel with definitions of the symbols used, but without detailing methodology.

TABLES – main tables should be provided as individual Word files, together with a brief title and legend. For supplementary tables see below.

SUPPLEMENTARY INFORMATION – Supplementary information is material directly relevant to the conclusion of a paper, but which cannot be included in the printed version in order to keep the

manuscript concise and accessible to the general reader. Supplementary information is an integral part of a Nature Cell Biology publication and should be prepared and presented with as much care as the main display item, but it must not include non-essential data or text, which may be removed at the editor's discretion. All supplementary material is fully peer-reviewed and published online as part of the HTML version of the manuscript. Supplementary Figures and Supplementary Notes are appended at the end of the main PDF of the published manuscript.

Supplementary items should relate to a main text figure, wherever possible, and should be mentioned sequentially in the main manuscript, designated as Supplementary Figure, Table, Video, or Note, and numbered continuously (e.g. Supplementary Figure 1, Supplementary Figure 2, Supplementary Table 1, Supplementary Table 2 etc.).

Unprocessed scans of all key data generated through electrophoretic separation techniques need to be presented in a supplementary figure that should be labeled and numbered as the final supplementary figure, and should be mentioned in every relevant figure legend. This figure does not count towards the total number of figures and is the only figure that can be displayed over multiple pages, but should be provided as a single file, in PDF or TIFF format. Data in this figure can be displayed in a relatively informal style, but size markers and the figures panels corresponding to the presented data must be indicated.

The total number of Supplementary Figures (not including the "unprocessed scans" Supplementary Figure) should not exceed the number of main display items (figures and/or tables (see our Guide to Authors and March 2012 editorial http://www.nature.com/ncb/authors/submit/index.html#suppinfo; http://www.nature.com/ncb/journal/v14/n3/index.html#ed). No restrictions apply to Supplementary Tables or Videos, but we advise authors to be selective in including supplemental data.

Each Supplementary Figure should be provided as a single page and as an individual file in one of our accepted figure formats and should be presented according to our figure guidelines (see above). Supplementary Tables should be provided as individual Excel files. Supplementary Videos should be provided as .avi or .mov files up to 50 MB in size. Supplementary Figures, Tables and Videos much be accompanied by a separate Word document including titles and legends.

GUIDELINES FOR EXPERIMENTAL AND STATISTICAL REPORTING

REPORTING REQUIREMENTS – To improve the quality of methods and statistics reporting in our papers we have recently revised the reporting checklist we introduced in 2013. We are now asking all life sciences authors to complete two items: an Editorial Policy Checklist (found here https://www.nature.com/authors/policies/Policy.pdf) that verifies compliance with all required editorial policies and a Reporting Summary (found here https://www.nature.com/authors/policies/ReportingSummary.pdf) that collects information on experimental design and reagents. These documents are available to referees to aid the evaluation of the manuscript. Please note that these forms are dynamic 'smart pdfs' and must therefore be downloaded and completed in Adobe Reader. We will then flatten them for ease of use by the reviewers. If you would like to reference the guidance text as you complete the template, please access these flattened versions at http://www.nature.com/authors/policies/availability.html.

STATISTICS – Wherever statistics have been derived the legend needs to provide the n number (i.e.

the sample size used to derive statistics) as a precise value (not a range), and define what this value represents. Error bars need to be defined in the legends (e.g. SD, SEM) together with a measure of centre (e.g. mean, median). Box plots need to be defined in terms of minima, maxima, centre, and percentiles. Ranges are more appropriate than standard errors for small data sets. Wherever statistical significance has been derived, precise p values need to be provided and the statistical test used needs to be stated in the legend. Statistics such as error bars must not be derived from n<3. For sample sizes of n<5 please plot the individual data points rather than providing bar graphs. Deriving statistics from technical replicate samples, rather than biological replicates is strongly discouraged. Wherever statistical significance has been derived, precise p values need to be provided and the statistical test stated in the legend.

Information on how many times each experiment was repeated independently with similar results needs to be provided in the legends and/or Methods for all experiments, and in particular wherever representative experiments are shown.

We strongly recommend the presentation of source data for graphical and statistical analyses as a separate Supplementary Table, and request that source data for all independent repeats are provided when representative experiments of multiple independent repeats, or averages of two independent experiments are presented. This supplementary table should be in Excel format, with data for different figures provided as different sheets within a single Excel file. It should be labelled and numbered as one of the supplementary tables, titled “Statistics Source Data”, and mentioned in all relevant figure legends.

--------- Please don't hesitate to contact NCB@nature.com should you have queries about any of the above requirements ---------

Author Rebuttal, first revision:

Point-by-Point Response to Reviewers’ Comments

We thank the Reviewers and the Editor for recognizing the efforts made in the revision process. As detailed below in our point-by-point response, we have addressed the remaining points raised by the Referees and now hope the manuscript could be accepted for publication.

Point-by-point response to Referee 1:

Overall, the revised manuscript is significantly improved. The authors have done a great job. While the authors have adequately addressed all the questions raised previously, I do have a couple of additional comments.

Major issues:

The authors have not discussed deeply the relevance of their TERRA lncRNA-related analysis to the rest of their studies. The authors only described that TERRA lncRNA accessibility and expression are altered upon Daxx KO, but it was not clear how does this link to the rest of the studies. Do the authors think TERRA lncRNA contributes to the hematopoiesis dysregulation they observed, and how? Will it be likely to be Pu.1 dependent? If so, did the authors investigate whether Daxx/Pu.1 DKO could reverse TERRA upregulation?

Reply: We thank the reviewer for the positive response to our revisions. We agree that the TERRA-related work seemed somewhat disconnected from the rest of the studies presented in our manuscript. We have therefore covered this in the discussion, while also adding further data based on the Reviewer's suggestions. Analysis of TERRA expression relies on Northern blotting, thus making it not possible in small subpopulations of progenitors, such as LT-HSCs or KLS cells. Other methods are unreliable and not extensively validated in the literature, so we decided to make best use of available ATAC- and RNA-seq datasets in the context of LT-HSCs and more committed progenitors. In particular, the **first set** of findings is summarized below:

i) As shown in **Ext. Fig. 1i,j**, opening of chromatin at TERRA-binding sites (TERRA-BS) is detected at both 3wpi (3 weeks post induction) and in acute KO settings (3dpi, 3 days post induction). This applies to all chromosomes.

ii) These changes correlate with downregulation of known TERRA targets such as the sex-chromosome-associated *Erdr1* and *Mid1* genes, as well as the autosomal *Wls* gene.

iii) Genes that are differentially expressed upon TERRA KD in part overlap with DEGs in Daxx KO GMPs, suggesting that indeed altered TERRA expression and/or function in Daxx-deficient cells may contribute to the observed phenotypes (these data were not included in previously revised version of the manuscript, but we thought they would be quite relevant and therefore they have now been re-added to the manuscript; **Ext Data Fig 7m**).

Interestingly, the effect of TERRA KD (Chu et al Cell 2017; PMID 28666128) has been reported to correlate with reduced expression of neighboring genes as well, thus suggesting that in the absence of Daxx, TERRA is unable to promote gene expression (even if upregulated). As KD of Atrx, a Daxx and TERRA interactor increases expression of TERRA targets, Atrx and Daxx may functionally antagonize each other for TERRA regulation.

The **second set of data** relates to the effect of concomitant Pu.1 and Daxx loss on chromatin landscape and transcription at TERRA-BS (**Ext. Data Fig. 8h,i**):

i) We showed that opening of TERRA-BS observed in Daxx KO KLS cells is reverted by concomitant Daxx and Pu.1 loss, but this effect is restricted to sex chromosomes.

ii) Changes in chromatin landscape correlate with rescued expression levels of selected TERRA targets.

These findings reinforce the possibility that TERRA may contribute to some of the phenotypes caused by Daxx loss. In particular, progenitors skewed towards a myeloid fate via Pu.1 engagement may require TERRA for these changes in lineage specification. Further work is required to assess the potential involvement of TERRA as mediator of myeloid vs lymphoid output in hematopoiesis.

Minor issues:
Figure legend
1) In the legend for Figure 1, point j describes Figure 1k, while point k describes Figure 1j.
2) In Figure 7j, it would help the reader if the left, middle and right panel are labelled (e.g WT, Daxx KO, DKO).
3) In the Extended Data Fig. 5b, the legend color for Daxx+/+ Hira+/+ (white) does not correspond to the bar color (black bar)
4) In Extended Data Fig. 7h, it would help the reader if the upper, middle and lower panel are labelled (e.g 3 wpi, 24 wpi, transplantation)

Reply: all these minor issues have been addressed.

Point-by-point response to Referee 3:

In the revised version by Gerber et al, series of experiments have been added and an important work has been carried out to address issues raised by the three reviewers concerning the first submitted manuscript. Significant work has been carried out considering most questions but the outcome and interpretations remain still extremely difficult at this point in time to get a clear-cut conclusion, at least for a reviewer that is not a specialist of the hematopoietic system.
While they did document the fact that aberrant chromatin landscapes arise upon the loss of Daxx (under their experimental conditions) and that there is a Pu.1-mediated neutrophilia and inflammation, unfortunately it is still unclear about what is actually happening and what is the underlying mechanism. The additional work further emphasizes the difficulty to draw clear conclusions. However, they did provide a lot of data, this should be acknowledged. Furthermore, the authors were very cautious and balanced in their interpretations. Should specialist feel satisfied with the relevance for the hematopoietic system, I could support publication. I would still recommend to make a serious effort to help readers by trying to screen their manuscript to clarify as much as possible the messages to reach out to a general audience in cell biology.

Reply: We are thankful that the Reviewer has recognized the important work carried out to address her/his comments and the ones raised by the other Referees. We have now worked extensively to make this study more concise and its conclusions clearer, also for the general audience in cell biology. We believe the main advance we have made during the revision process is highlighting a potential genetic interaction between Daxx, a key heterochromatin player and Pu.1, a master regulator of hematopoiesis. More work remains to investigate the precise underlying mechanisms, but we believe the current study proposes a novel concept implicating intrinsic immunity mechanisms repressing repeat elements in the control of lineage specification during hematopoiesis. This may have implications for our understanding of how heterochromatin perturbations may contribute to inflammation and neoplastic transformation within the hematopoietic system.

Point-by-point response to Referee 4:

Gerber et al identify a clear role for Daxx in maintaining the chromatin landscape of LT-HSCs, and associate this with aberrant gene expression. They have not (been able to) perform transplantation studies, but I feel that these are not absolutely required to substantiate their conclusions. In their paper they do not make any claims on the role of Daxx for HSC-specific functionality, so I do not believe that classical HSC (serial) transplantation experiments are essential for this paper.

Reply: We are pleased that the Reviewer agrees that our study clearly implicates Daxx in chromatin landscape maintenance in HSCs and that classical HSC experiments are not required for this paper.

Decision Letter, second revision:

Dear Paolo,

Thank you for submitting your revised manuscript "Aberrant chromatin landscape upon loss of the H3.3 chaperone Daxx leads to Pu.1–mediated neutrophilia and inflammation" (NCB-S42550B).

It has now been seen again by original referee 1 and their comments are below. The reviewer is now satisfied with the revisions and therefore we'll be happy in principle to publish it in Nature Cell Biology, pending minor revisions to comply with our editorial and formatting guidelines.

We are now performing detailed checks on your paper and will send you a checklist detailing our editorial and formatting requirements in about 1-2 weeks. Please do not upload the final materials and make any revisions until you receive this additional information from us.

Thank you again for your interest in Nature Cell Biology. Please do not hesitate to contact me if you have any questions.

With best wishes,

Christine.

Christine Weber, PhD
Senior Editor
Nature Cell Biology
E-mail: christine.weber@nature.com
Phone: +44 (0)207 843 4924

Reviewer #1 (Remarks to the Author):

Authors have addressed all the questions raised. The relevance of TERRA lncRNA in Daxx/Pu.1 KO is now discussed better in their manuscript.
All the minor issues has been corrected as well.

Final Decision Letter:

Dear Paolo,

I am pleased to inform you that your manuscript, "Aberrant chromatin landscape upon loss of the H3.3 chaperone Daxx in hematopoietic precursors leads to Pu.1–mediated neutrophilia and inflammation", has now been accepted for publication in Nature Cell Biology.

Thank you for sending us the final manuscript files to be processed for print and online production, and for returning the manuscript checklists and other forms. Your manuscript will now be passed to our production team who will be in contact with you if there are any questions with the production quality of supplied figures and text.

In approximately 10 business days you will receive an email with a link to choose the appropriate publishing options for your paper and our Author Services team will be in touch regarding any additional information that may be required.

You will not receive your proofs until the publishing agreement has been received through our system.

If you have any questions about our publishing options, costs, Open Access requirements, or our legal forms, please contact ASJournals@springernature.com

Before the manuscript is sent to our printers, we will make changes in the text that may be necessary either to make it conform with house style or to make it intelligible to our wide readership. We look particularly carefully at the titles of all papers to ensure that indexing will be accurate and that they are not unreasonably long. We will ask your approval before the copy is finalized, and you will soon receive the edited proofs. Please check the text and figures carefully. Once your manuscript is typeset and you have completed the appropriate grant of rights, you will receive a link to your electronic proof via email with a request to make any corrections within 48 hours. If, when you receive your proof, you cannot meet this deadline, please inform us at rjsproduction@springernature.com immediately.

Once your paper has been scheduled for online publication, the Nature press office will be in touch to confirm the details. An online order form for reprints of your paper is available at https://www.nature.com/reprints/author-reprints.html. All co-authors, authors' institutions and authors' funding agencies can order reprints using the form appropriate to their geographical region.

Publication is conditional on the manuscript not being published elsewhere and on there being no

announcement of this work to any media outlet until the online publication date in Nature Cell Biology.

Please note that *Nature Cell Biology* is a Transformative Journal (TJ). Authors may publish their research with us through the traditional subscription access route or make their paper immediately open access through payment of an article-processing charge (APC). Authors will not be required to make a final decision about access to their article until it has been accepted. Find out more about Transformative Journals

Authors may need to take specific actions to achieve compliance with funder and institutional open access mandates. For submissions from January 2021, if your research is supported by a funder that requires immediate open access (e.g. according to Plan S principles) then you should select the gold OA route, and we will direct you to the compliant route where possible. For authors selecting the subscription publication route our standard licensing terms will need to be accepted, including our self-archiving policies. Those standard licensing terms will supersede any other terms that the author or any third party may assert apply to any version of the manuscript.

To assist our authors in disseminating their research to the broader community, our SharedIt initiative provides you with a unique shareable link that will allow anyone (with or without a subscription) to read the published article. Recipients of the link with a subscription will also be able to download and print the PDF.

If your paper includes color figures, please be aware that in order to help cover some of the additional cost of four-color reproduction, Nature Research charges our authors a fee for the printing of their color figures. Please contact our offices for exact pricing and details.

As soon as your article is published, you will receive an automated email with your shareable link.

If you have not already done so, we strongly recommend that you upload the step-by-step protocols used in this manuscript to the Protocol Exchange (www.nature.com/protocolexchange), an open online resource established by Nature Protocols that allows researchers to share their detailed experimental know-how. All uploaded protocols are made freely available, assigned DOIs for ease of citation and are fully searchable through nature.com. Protocols and the Nature and Nature research journal papers in which they are used can be linked to one another, and this link is clearly and prominently visible in the online versions of both papers. Authors who performed the specific experiments can act as primary authors for the Protocol as they will be best placed to share the methodology details, but the Corresponding Author of the present research paper should be included as one of the authors. By uploading your Protocols to Protocol Exchange, you are enabling researchers to more readily reproduce or adapt the methodology you use, as well as increasing the visibility of your protocols and papers. You can also establish a dedicated page to collect your lab Protocols. Further information can be found at www.nature.com/protocolexchange/about

You can use a single sign-on for all your accounts, view the status of all your manuscript submissions and reviews, access usage statistics for your published articles and download a record of your refereeing activity for the Nature journals.

Please feel free to contact us if you have any questions.

All the best,

natureresearch

Christina

Christina Kary, PhD
Chief Editor
Nature Cell Biology
1 New York Plaza
Tel: +44 (0) 207 843 4924

Click here if you would like to recommend Nature Cell Biology to your librarian
http://www.nature.com/subscriptions/recommend.html#forms

** Visit the Springer Nature Editorial and Publishing website at www.springernature.com/editorial-and-publishing-jobs for more information about our career opportunities. If you have any questions please click here.**
